# The MiR-135b–BMAL1–YY1 loop disturbs pancreatic clockwork to promote tumourigenesis and chemoresistance

**DOI:** 10.1038/s41419-017-0233-y

**Published:** 2018-02-02

**Authors:** Weiliang Jiang, Senlin Zhao, Jia Shen, Lihong Guo, Yi Sun, Yuntian Zhu, Zhixiong Ma, Xin Zhang, Yangyang Hu, Wenqin Xiao, Kai Li, Sisi Li, Li Zhou, Li Huang, Zhanjun Lu, Yun Feng, Junhua Xiao, Eric Erquan Zhang, Lijuan Yang, Rong Wan

**Affiliations:** 1 Department of Gastroenterology, Shanghai General Hospital, Shanghai Jiao Tong University School of Medicine, Shanghai, China; 2 Shanghai Key Laboratory of Pancreatic Disease, Institute of Pancreatic Disease, Shanghai Jiao Tong University School of Medicine, Shanghai, China; 3 Department of General Surgery, Shanghai General Hospital, Shanghai Jiao Tong University School of Medicine, Shanghai, China; 4 Tumour Initiation and Maintenance Program, NCI-designated Cancer Center, Sanford Burnham Prebys Medical Discovery Institute, La Jolla, CA USA; 5 Department of Gastroenterology, Central Hospital of Shengli Oil-field, Dongying, Shandong China; 6 Shanghai Tenth People’s Hospital, Tongji University School of Medicine, Shanghai, China; 7 Department of Anesthesiology, Zhejiang Cancer Hospital, Hangzhou, Zhejing China; 8 National Institute of Biological Sciences, Beijing, China; 9 Department of Pathology, Zhejiang Province People’s Hospital, Hangzhou, Zhejiang China; 10 Department of Gastroenterology, Shanghai East Hospital, Tongji University School of Medicine, Shanghai, China

## Abstract

Circadian disruption has been implicated in tumour development, but the underlying mechanism remains unclear. Here, we show that the molecular clockwork within malignant human pancreatic epithelium is disrupted and that this disruption is mediated by miR-135b-induced BMAL1 repression. miR-135b directly targets the BMAL1 3′-UTR and thereby disturbs the pancreatic oscillator, and the downregulation of miR-135b is essential for the realignment of the cellular clock. Asynchrony between miR-135b and BMAL1 expression impairs the local circadian gating control of tumour suppression and significantly promotes tumourigenesis and resistance to gemcitabine in pancreatic cancer (PC) cells, as demonstrated by bioinformatics analyses of public PC data sets and in vitro and in vivo functional studies. Moreover, we found that YY1 transcriptionally activated miR-135b and formed a ‘miR-135b–BMAL1–YY1’ loop, which holds significant predictive and prognostic value for patients with PC. Thus, our work has identified a novel signalling loop that mediates pancreatic clock disruption as an important mechanism of PC progression and chemoresistance.

## Introduction

Pancreatic cancer (PC) is one of the most prevalent digestive malignancies and is the fourth most common cause of cancer-related death^1^. The dismal prognosis of PC is attributed to its propensity for early local invasion and metastasis, its profound chemotherapy resistance, and the lack of effective diagnostic and therapeutic strategies^2^. Notably, PC morbidity and mortality is much higher in more developed regions and is quickly increasing in developing countries^3^, which suggests that changes in lifestyle and environment may strongly influence the development of this highly lethal disease.

Over the past several decades, circadian misalignment resulting from genetic changes and behavioural (e.g. shift work and late evening activities) or metabolic (e.g. obesity and diabetes) modifications has been linked to tumour pathologies^4–7^. As an endogenous adaptive system that oscillates over an ~24-h period, the strictly hierarchical mammalian circadian clock consists of a central pacemaker in the suprachiasmatic nucleus and multiple peripheral oscillators^8^. The pancreas, a well-characterised peripheral clock harbours highly self-autonomous time-keeping systems that regulate pancreatic biological processes at both the organic and cellular levels^9,10^. Molecularly, the pancreatic oscillator is operated by several interlocking transcription–translation feedback loops (TTFLs) of circadian genes including *BMAL1*, *CLOCK*, *PERs*, *CRYs*, *NR1D1* and *RORA*, which are present in almost all exocrine and endocrine cells^11^. Clock genes and their numerous downstream targets, known as clock-controlled genes (CCGs), maintain circadian homoeostasis and can become altered during tumour development^12^. However, whether the local clockwork in the pancreas is reprogrammed by oncogenic alterations and the role of a disrupted pancreatic clock in PC progression are still unclear.

*BMAL1* is a key circadian clock gene that regulates tissue homoeostasis, metabolism and ageing, and it is the only single clock gene knockout in which the experimental animals lose rhythmic behavioural activities^13^. The dysregulation of BMAL1 has been documented in various tumours, such as haematological malignancies^14^, lung cancer^15^, osteosarcoma^16^ and PC^17–19^. We previously demonstrated that decreased BMAL1 expression in PC suppresses the p53 pathway and significantly enhances tumour growth^19^. However, the mechanism underlying BMAL1 deregulation in tumourigenesis is still unknown.

MicroRNAs (miRNAs) are small non-coding RNAs that regulate gene expression by binding to complementary sequences on the 3′-untranslated region (3′-UTR) of target mRNAs. As potent post-transcriptional regulators, an increasing number of studies have implicated miRNAs in the modulation of oscillatory properties and time-keeping functions^20,21^. Importantly, several miRNAs (e.g. miR-219, miR-132, miR-142 and miR-92a^22–24^) also exhibit robust rhythms. Translational control via miRNAs may, therefore, represent an important regulatory mechanism of the circadian clock. In this study, we identified hsa-miR-135b as an oncogenic miRNA that promotes tumourigenesis and chemoresistance in human PC. By directly targeting the 3′-UTR, miR-135b suppressed BMAL1 expression and thereby disturbed the entire molecular clockwork inside the exocrine pancreas, leading to impaired circadian control of tumour suppression. Intriguingly, we observed that the transcription factor Yin Yang 1 (YY1) directly activated the promoter of miR-135b and formed a ‘miR-135b–BMAL1–YY1’ loop, whose expression was related to the clinicopathological factors, survival outcomes and chemoresponsiveness in patients with PC.

## Results

### The local clockwork is disrupted in PC

**Fig. 1:**
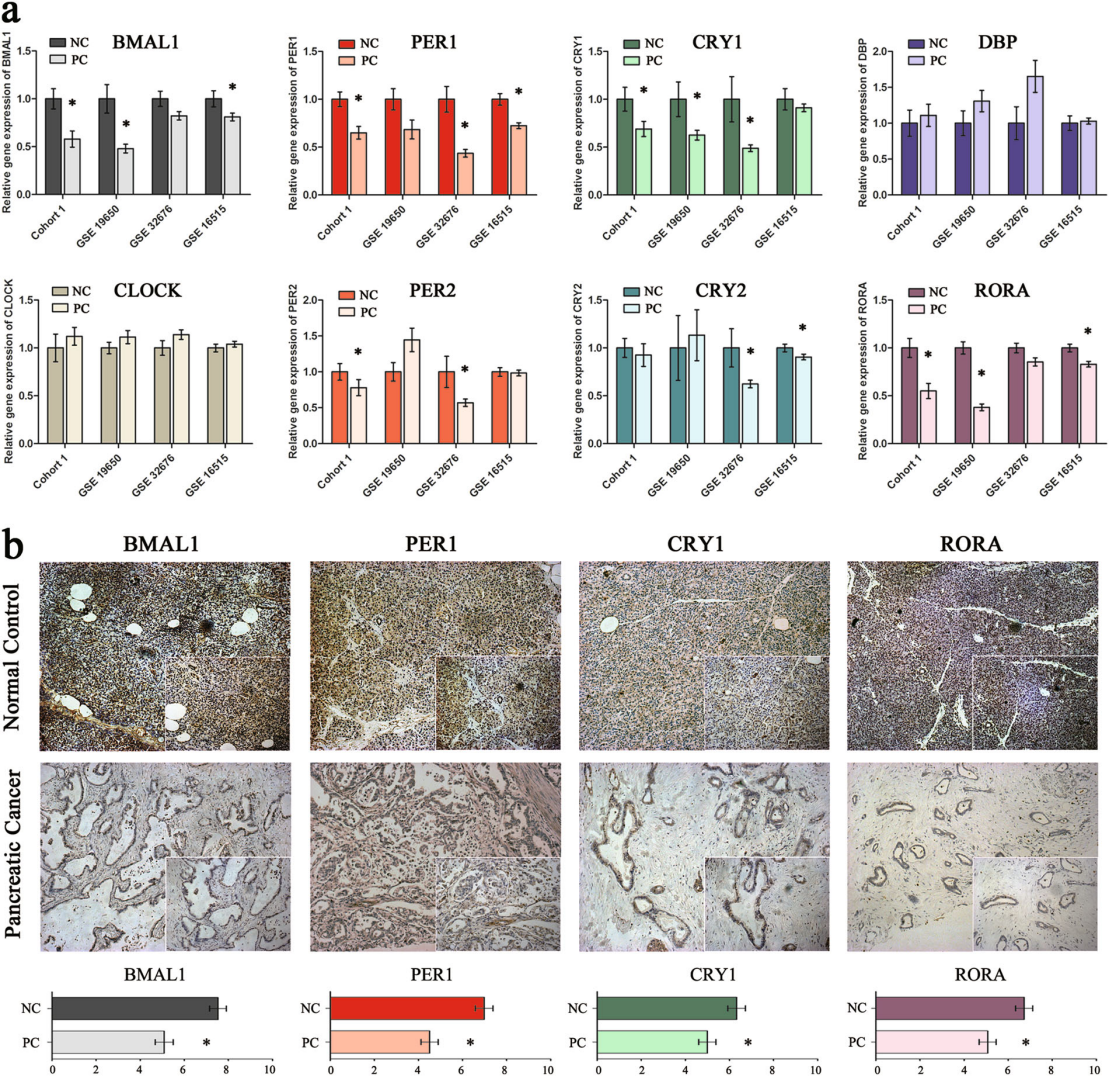
Unravelling a disrupted molecular clockwork in human PC tissues. **a** Expression of eight core clock genes in PC and normal pancreatic tissues (NC) in four independent PC cohorts (cohort 1, GSE19650, GSE32676 and GSE16515). Data are presented as the means ± SEM. *P < 0.05 as compared with the NC groups. **b** Representative immunochemistry analyses of four circadian proteins in PC and NC tissues from cohort 1 patients. Original magnification ×100; zoomed sections ×200. The IHC scores are shown in the bottom. *P < 0.05

We first investigated the expression of core circadian genes including *BMAL1*, *CLOCK*, *PER1/2*, *CRY1/2*, *DBP* and *RORA* in four individual PC cohorts. Cohort 1 included 55 pairs of PC and normal controls; the other three cohorts were public human genome arrays of PC and normal pancreatic tissue (GSE19650, GSE32676 and GSE16515) downloaded from the NCBI Gene Expression Omnibus (GEO). As a result, all eight genes involved in the central circadian TTFLs exhibited aberrant expression; among these, the expression levels of *BMAL1*, *PER1*, *CRY1* and *RORA* were significantly decreased in at least three cohorts (Fig. 1a; Supplementary Tables S1–S3). Immunohistochemistry (IHC) analysis was then performed in cohort 1 samples. We found that these four clock genes were mainly present in the nucleus and cytoplasm of normal cells but were not present in the malignant epithelium. IHC score quantification confirmed that the protein levels of BMAL1, PER1, CRY1 and RORA were markedly decreased in PC tissues. Of the 55 normal controls, 45, 41, 37 and 40 samples presented with strong positive staining for BMAL1, PER1, CRY1 and RORA, respectively. In contrast, only 26, 21, 28 and 28 of PC tissues had high-expression levels of BMAL1, PER1, CRY1 and RORA, respectively (Fig. 1b). Altogether, these results revealed an altered molecular clockwork profile in human PC.

### Identification of miR-135b as a negative regulator of BMAL1

**Fig. 2:**
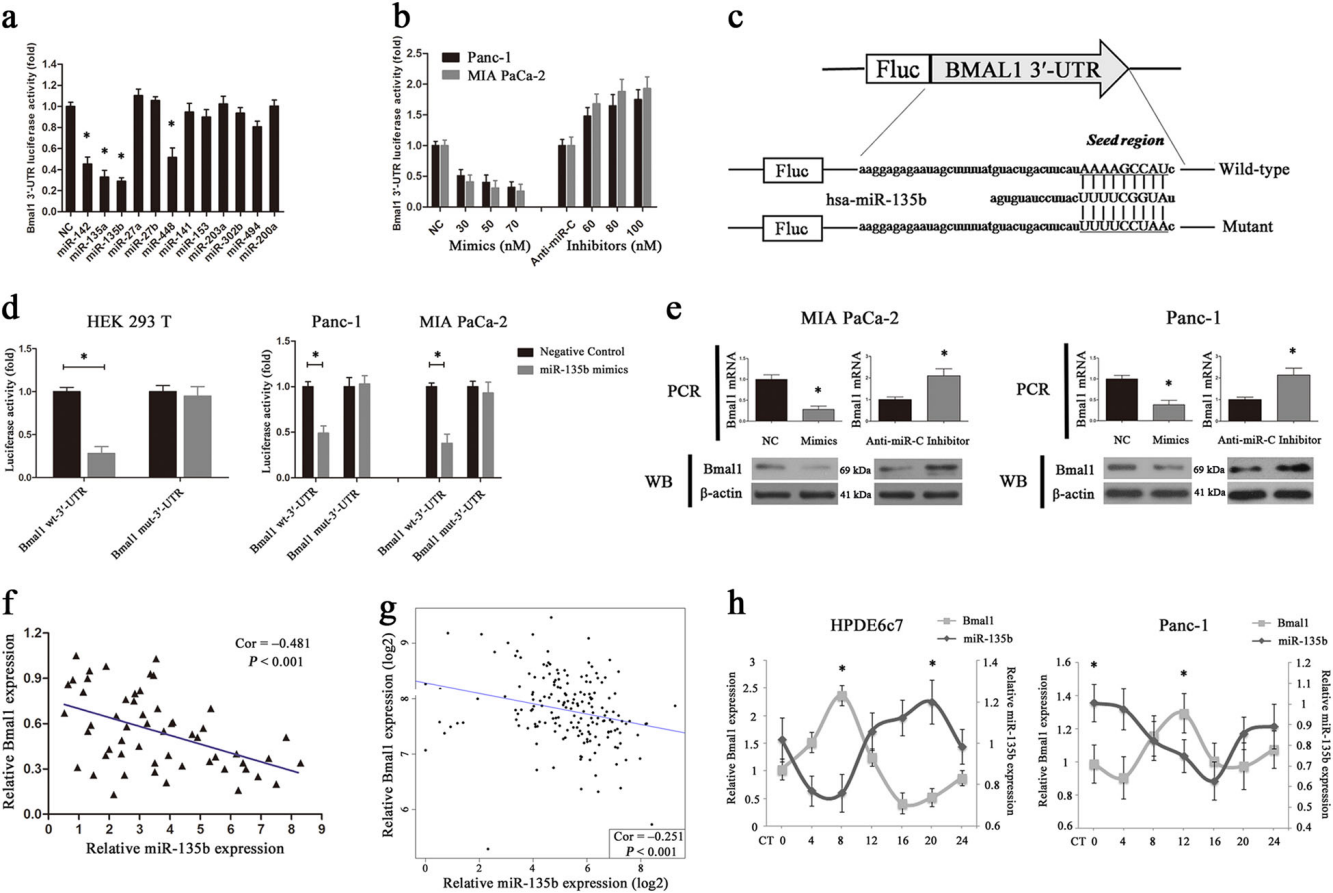
Identification of hsa-miR-135b as a negative regulator of BMAL1. **a** Relative luciferase activity of BMAL1 3′-UTR in HEK 293T cell transfected with predicted miRNAs (30 nM) or negative control (NC) as indicated. The results were normalised to the NC group, and the mean of the NCs was set to 1. *P < 0.05. **b** Luciferase activity of BMAL1 3′-UTR in Panc-1 or MIA PaCa-2 cells transfected with different doses of oligonucleotides as indicated. **c** A human BMAL1 3′-UTR fragment containing the wild-type or mutant miR-135b-binding sequence was cloned downstream to the luciferase reporter gene. **d** Relative luciferase activity of the wild-type or mutant BMAL1 3′-UTR in HEK 293T cells (left) and in human PC cells (right) transfected with miR-135b mimics or NCs. **e** The mRNA and protein levels of BMAL1 in miR-135b mimics (70 nM)/inhibitors (100 nM)- or their respective controls-transfected PC cells. **f**, **g** Correlation analysis of miR-135b and BMAL1 mRNA expressions in PC tissues from cohort 1 (55 cases, **f**) or TCGA cohort (178 cases, **g**). **h** Quantitative RT-PCR analysis of miR-135b and BMAL1 expressions in HPDE6c7 and Panc-1 cells at 4 h intervals over a 24-h period. *P < 0.05, peak vs. nadir. Data are shown as the means ± SEM for at least three independent experiments

Given the importance of BMAL1 in maintaining circadian operations, we sought to explore the possible post-transcriptional regulation of its downregulation in PC. Five miRNA-target prediction programmes (TargetScan, miRDB, miRanda, PicTar and MicroCosm) were used to identify potential miRNAs targeting the 3′-UTR of BMAL1. Only miRNAs shown to bind to the same region of the target 3′-UTR sequence in at least three programmes were selected. Here, we identified 12 miRNAs that had potential binding sites in the 3′-UTR of BMAL1 (Supplementary Table S4). Co-transfection of the predicted miRNA mimics with luciferase reporter constructs containing wild-type BMAL1 3′-UTR into HEK 293T cells revealed that miR-142, miR-448, miR-135a and miR-135b significantly reduced luciferase activity (Fig. 2a). Additional qRT-PCR analysis showed that miR-135b was the only upregulated miRNA in cohort 1 PC tissues (Supplementary Fig. S1). We then transfected miR-135b mimics/inhibitors with BMAL1 luciferase reporter constructs into MIA PaCa-2 and Panc-1 cells, which presented with the relatively lowest and highest levels of miR-135b, respectively, among the PC cell lines (Supplementary Fig. S2). As expected, miR-135b mimics significantly inhibited the luciferase activity of the BMAL1 3′-UTR in a dose-dependent manner, whereas miR-135b inhibitors induced a drastic increase in luciferase activity (Fig. 2b). Thereafter, a luciferase reporter construct containing a mutated putative target site in the BMAL1 3′-UTR was generated (Fig. 2c). We found that compared with a significant reduction in the activity of the wild-type controls, miR-135b upregulation in HEK 293T cells or PC cells did not affect the activity of mutant BMAL1 3′-UTR (Fig. 2d) that demonstrated the specificity of this inhibition.

Subsequently, RT-PCR and western blotting revealed that miR-135b mimics markedly reduced the mRNA and protein levels of BMAL1, whereas miR-135b inhibitors increased BMAL1 expression in PC cells (Fig. 2e). The expression levels of miR-135b and BMAL1 in cohort 1 were negatively correlated (55 cases, Pearson *r* = –0.481, *P* < 0.001; Fig. 2f), and this result was further validated by bioinformatics analysis using high throughput RNA-sequencing data from The Cancer Genome Atlas (TCGA) PC cohort (178 cases, Pearson *r* = –0.251, *P* < 0.001; Fig. 2g, Supplementary Table S5). As the *BMAL1* gene oscillates rhythmically in vivo, we explored whether miR-135b also harbours self-sustained oscillation by assessing its temporal pattern in HPDE6c7 and Panc-1 cells at 4-h intervals over a 24-h period. As shown in Fig. 2h, in both the synchronised normal and malignant pancreatic cells, the relative abundance of miR-135b was marked by rhythmic variations (*P* < 0.05) that were roughly in anti-phase with those of *BMAL1*. Collectively, these findings confirmed that miR-135b is a BMAL1-targeting miRNA in PC.

### miR-135b perturbs the clock machinery in pancreatic duct epithelial cells

**Fig. 3:**
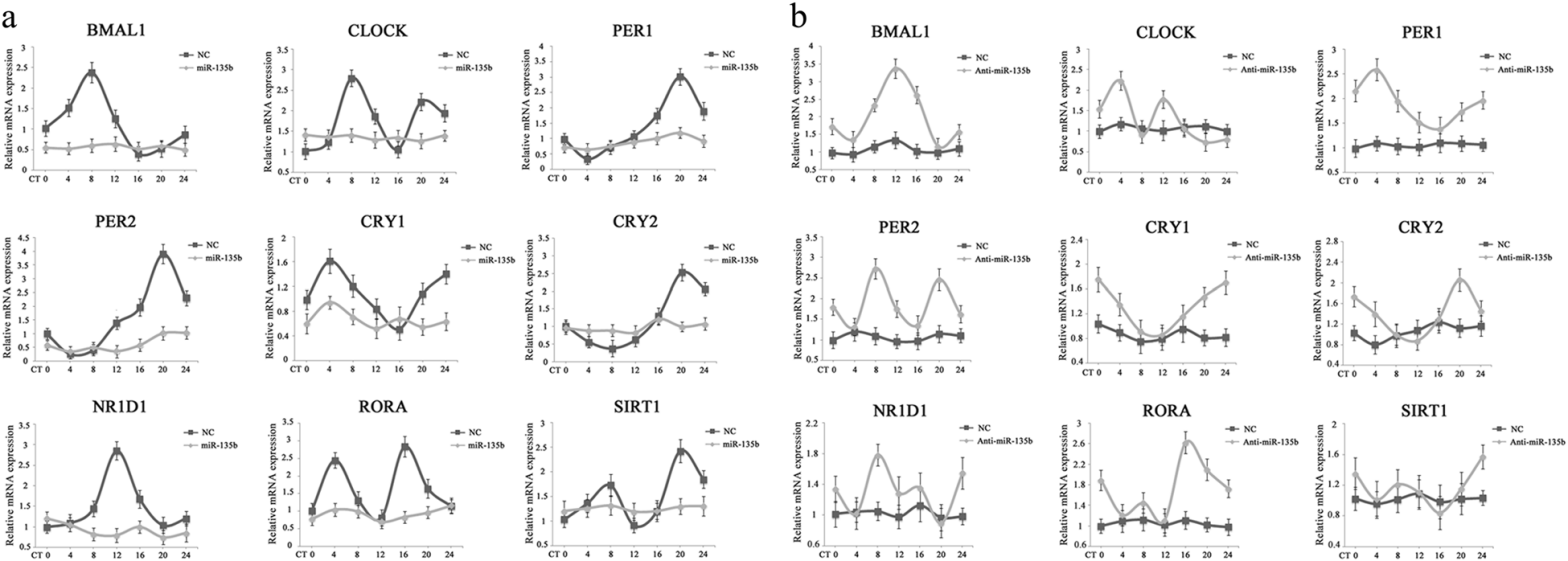
The role of miR-135b in regulating the pancreatic epithelial clockwork. **a**, **b** Temporal expression patterns of nine core circadian genes were determined by qRT-PCR in HPDE6c7 cells transfected with miR-135b expression vectors/empty vectors (NCs) (**a**) and in Panc-1 cells transfected with miR-135b knockdown vectors/NCs (**b**). Cells were serum-shocked before experiment. Cellular RNA samples were harvested every 4 h until 24 h. *GAPDH* was used as an internal standard. Error bars represent SEM. The circadian parameters including period, phase, and amplitude were analysed by the JTK_CYCLE algorithm and were shown in Supplementary Table S6

Having shown the direct regulation of BMAL1 by miR-135b, we next examined the influence of miR-135b on central circadian TTFLs within normal and malignant pancreatic epithelial cells. The 24-h expression profiles of clock genes were monitored by RT-PCR and were further analysed by the JTK_CYCLE algorithm^25^. As a result, robust oscillations (*P* < 0.05) of all nine genes, *BMAL1*, *CLOCK*, *PER1/2*, *CRY1/2*, *NR1D1*, *RORA* and *SIRT1*, were observed in serum-shocked HPDE6c7 cells; in contrast, the endogenous rhythm was disturbed in PC cells (Supplementary Table S6). We then manipulated the miR-135b level using expression or knockdown vectors and we found that overexpressing miR-135b in HPDE6c7 cells significantly dampened the rhythms of *PER1* and *PER2* by shortening their amplitudes, and caused arrhythmic expression of *BMAL1*, *CLOCK*, *CRY1/2*, *NR1D1*, *RORA* and *SIRT1* (Fig. 3a). Meanwhile, the downregulation of miR-135b in Panc-1 cells rescued the diurnal fluctuations of *BMAL1*, *PER1/2*, *CRY1/2* and *RORA* (Fig. 3b). These observations clearly suggested that miR-135b is an important regulator of the pancreatic time-keeping system.

### Dysregulation of the miR-135b–BMAL1 axis impairs clock-controlled tumour suppression

**Fig. 4:**
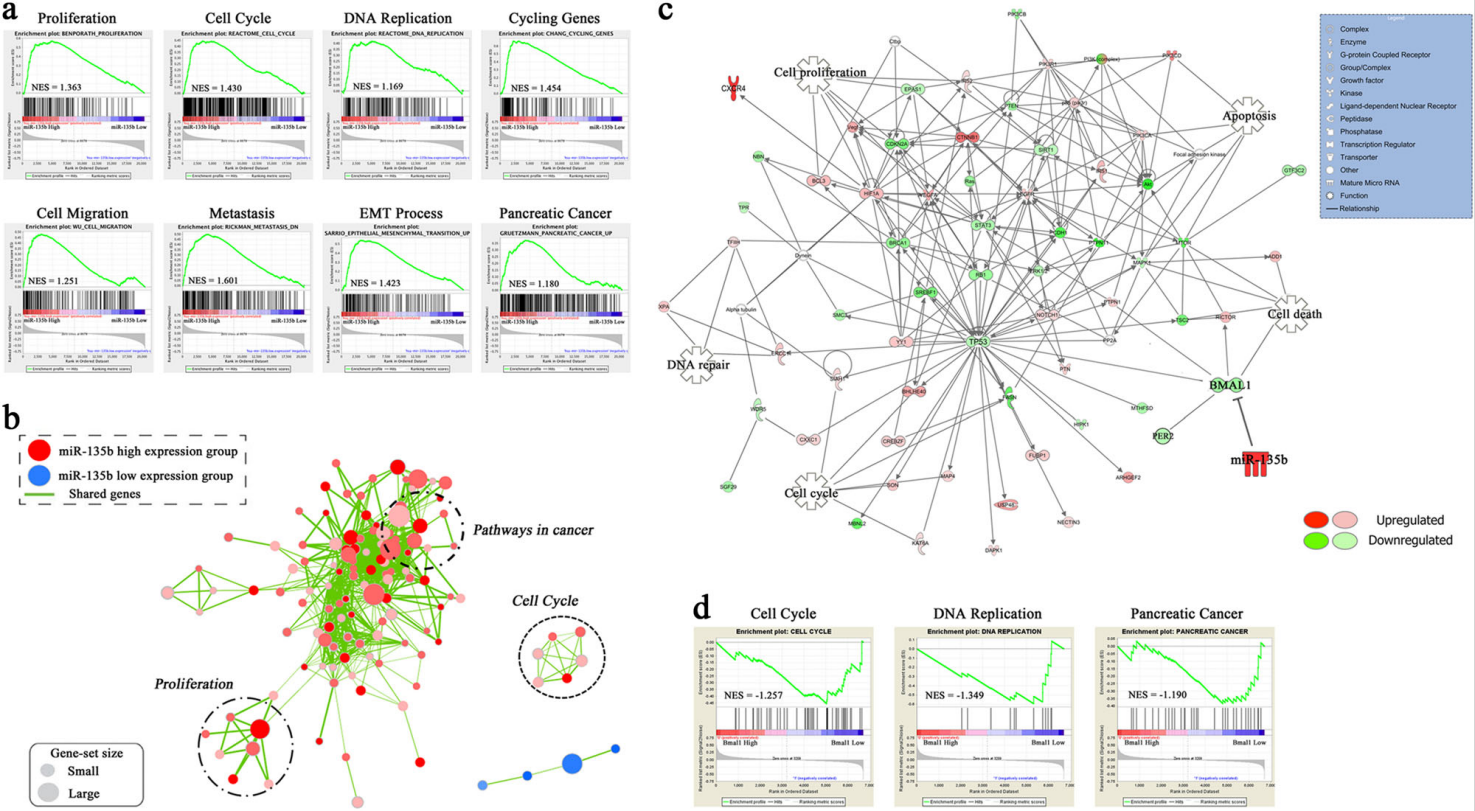
Biological implications of the miR-135b–BMAL1 axis in PC. **a**, **b** GSEA comparing PC patients with high miR-135b expression (red) against patients with low miR-135b expression (blue) in TCGA data set (median split, *n* = 178). Enrichment map and cytoscape were used for visualisation of the distinct biological processes and pathways between the two group patients. Enrichment results were mapped as a network of gene sets (nodes). Nodes represent enriched gene sets that are grouped and annotated by their similarity. Node size is proportional to the total number of genes within each gene set. Proportion of shared genes between gene sets is represented as the thickness of the green line between nodes. **c** IPA profiling putative molecular networks associated with BMAL1 downregulation based on microarray data from the GSE19650 PC cohort. Green icons indicate the genes that are downregulated by BMAL1 repression, whereas red icons refer to the upregulated ones. Detailed IPA legends are shown in the table by the right side. **d** GSEA used to identify the differential gene profiles between BMAL1 high-expression group and low-expression group of patients in the GSE19650 PC cohort

To probe the miR-135b–BMAL1 axis-associated biological pathways in an unbiased manner, we performed a gene set enrichment analysis (GSEA) in the TCGA PC cohort. Enrichment plots of GSEA showed that the gene signatures of cell proliferation, cell cycle, DNA replication and cycling genes, which are responsible for PC growth, and the gene signatures of cell migration, metastasis and epithelial–mesenchymal–transition (EMT), which are crucial for PC progression, were enriched in patients with high miR-135b expression. Importantly, the PC gene set was also correlated with high miR-135b-expressing patients (Fig. 4a, b). To gain further insights, we performed an ingenuity pathway analysis (IPA) in the GSE19650 PC cohort and found that BMAL1 repression resulted in a distinct genomic transition. The paramount tumour suppressors, including p53, p16, RB1, BRCA1 and PTEN, were markedly downregulated (Fig. 4c). GSEA of the GSE19650 data set revealed that the gene signatures of cell cycle, DNA replication and PC were more associated with patients with low BMAL1 expression (Fig. 4d). These bioinformatics results consistently demonstrated that the miR-135b–BMAL1 axis has an important role in PC pathogenesis.

As the circadian clock targets CCGs to regulate cellular processes, we next investigated the influence of miR-135b on cancer-related CCGs^12,26^. Here, we showed that miR-135b overexpression in MIA PaCa-2 cells significantly altered the expression of clock-controlled cell cycle checkpoints (Supplementary Fig. S3a), the clock-controlled DNA repair system (Supplementary Fig. S3b) and clock-controlled apoptosis (Supplementary Fig. S3c), leading to enhanced cell cycle progression and the inhibition of cell apoptosis; however, the restoration of BMAL1 expression largely reversed these changes by rescuing the local circadian gating control.

### miR-135b–BMAL1 deregulation promotes PC tumourigenesis and chemoresistance

**Fig. 5:**
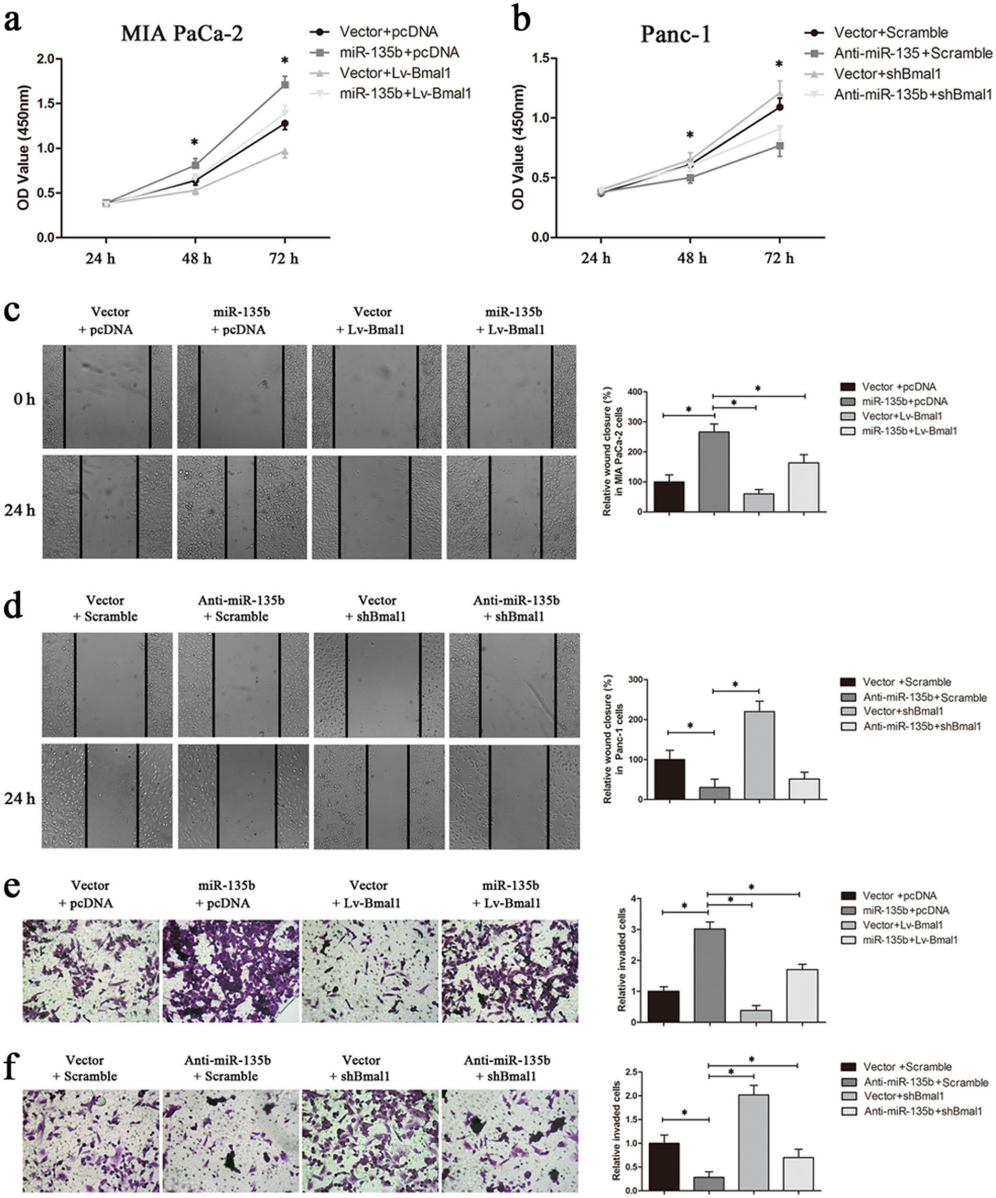
Effects of the miR-135b–BMAL1 axis on PC cell proliferation, migration and invasion. **a**, **b** The proliferation rates of MIA PaCa-2 and Panc-1 cells stably transfected with the indicated vectors were assessed by CCK-8 assays. The absorbance was measured at 450 nm. **c**–**f** In vitro wound-healing assays and transwell assays of MIA PaCa-2 cells (**c**, **e**) and Panc-1 cells (**d**, **f**) were performed for evaluating cell migration and invasion. Relative percent of wound closure and the number of cells invaded through the transwell membrane coated with matrigel were quantified after incubation for 24 h. Magnification ×200. Data are representative of at least three similar experiments. *P < 0.05

Further, MIA PaCa-2 and Panc-1 cells with manipulated miR-135b and/or BMAL1 expressions were used in in vitro gain-of-function and loss-of-function studies (Supplementary Fig. S4 shows the transfection efficiency). CCK-8 assays revealed that miR-135b overexpression increased the proliferation of MIA PaCa-2 cells, and this effect was remarkably antagonised by BMAL1 (Fig. 5a); however, knocking-down BMAL1 expression significantly increased the proliferation rate of Panc-1 cells, which was suppressed by miR-135b depletion (Fig. 5b). These observations were further confirmed by colony formation assays (Supplementary Fig. S5). Then, wound-healing and transwell assays were conducted, and we found that the ectopic expression of miR-135b in MIA PaCa-2 cells led to significantly faster wound closure and higher invasive activity than these parameters in the control cells; however, these enhancements were partially reversed after BMAL1 restoration (Fig. 5c, e). In contrast, silencing BMAL1 in Panc-1 cells markedly attenuated the inhibitory effect of miR-135b downregulation on cell migration and invasion (Fig. 5d, f). Collectively, these results validated that BMAL1 suppresses miR-135b-regulated PC progression.

**Fig. 6:**
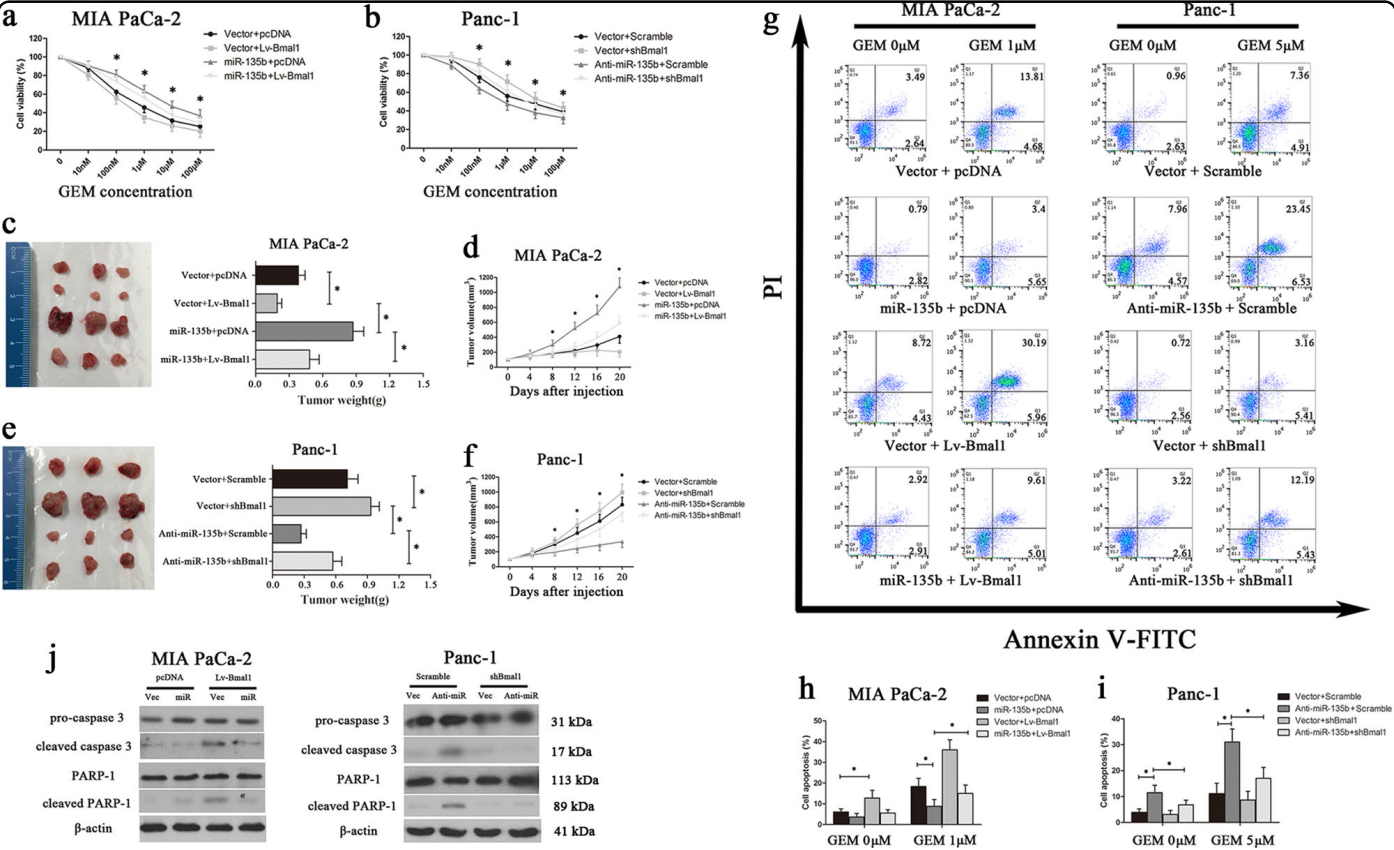
Effects of the miR-135b–BMAL1 axis on chemoresistance of PC cells. **a**, **b** Concentration-dependent inhibition of cell viability in response to gemcitabine (GEM) in MIA PaCa-2 or Panc-1 cells stably transfected with corresponding vectors. **c**–**f** Xenograft models were used to test the in vivo relevance of the miR-135b–BMAL1 axis and GEM resistance. Equal amounts of miR-135b and/or BMAL1-manipulated MIA PaCa-2 cells (**c**, **d**) or Panc-1 cells (**e**, **f**) were subcutaneously implanted on the back-side of each nude mouse. GEM treatment began at day 0. Tumour volume was monitored every 4 days (**d**,** f**). Mice were killed at the end of day 20 and xenografts were excised, photographed and weighed (**c**, **e**). **g** Representative dot-plots illustrating the apoptotic status of MIA PaCa-2 or Panc-1 cells using Annexin V-FITC/ PI method. Cells were treated with different doses of GEM (0, 1 or 5 μmol/L). The dot-plots in the right upper quadrant (Q2) and the right lower quadrant (Q3) were quantified as the percentage of apoptotic cells. Histograms indicated the proportion of cell apoptosis in MIA PaCa-2 (**h**) and Panc-1 cells (**i**). *P < 0.05. (**j**) Expression of the apoptosis-related proteins was detected by western blotting after a 72 h-exposure to GEM (1 μmol/L for MIA PaCa-2; 5 μmol/L for Panc-1). β-actin was used as internal control protein

As the major impediment for treating PC is the rapid development of chemoresistance to the current standard therapy, gemcitabine (GEM)^27^, we next investigated whether clock dysfunction affects the GEM response in PC cells. As shown in Fig. 6a, the IC_50_ values of MIA PaCa-2/Vector/pcDNA and MIA PaCa-2/miR-135b/pcDNA cells towards GEM were 1.11 and 9.79 μM, respectively, and were sharply reduced to 0.34 (MIA PaCa-2/Vector/Lv-BMAL1) and 3.05 (MIA PaCa-2/miR-135b/Lv-BMAL1) μM, respectively after BMAL1 restoration. Meanwhile, the IC_50_ values for GEM in Panc-1/Vector/Scramble, Panc-1/Vector/shBMAL1, Panc-1/Anti-miR-135b/Scramble and Panc-1/Anti-miR-135b/shBMAL1 cells were 8.61, 24.37, 2.12 and 8.56 μM, respectively (Fig. 6b). These data demonstrated that miR-135b-mediated BMAL1 suppression markedly facilitates GEM resistance in PC cells. Then, the in vivo relevance of miR-135b–BMAL1 to chemotherapy was tested using xenograft models. As anticipated, miR-135b overexpression promoted the growth of subcutaneous xenografts generated by MIA PaCa-2 cells, whereas BMAL1 reintroduction sensitised the cell response to GEM therapy and substantially reduced the sizes and weights of tumours (Fig. 6c, d). Similar results were also obtained from Panc-1 cell-derived tumours (Fig. 6e, f).

Then, we dissected the mechanism of GEM resistance. Flow cytometry analysis revealed that miR-135b significantly reduced the fraction of apoptotic MIA PaCa-2 cells during GEM treatment, whereas silencing miR-135b in Panc-1 cells led to a sharp increase in programmed cell death. Specifically, the modulation of BMAL1 expression was found to be effective against this miR-135b-mediated anti-apoptotic activity (Fig. 6g–i). We further measured the expression of apoptotic biochemical markers. After a 72 h-exposure to GEM, cleaved caspase-3 and PARP were significantly increased in BMAL1-upregulated MIA PaCa-2 cells and in miR-135b-downregulated Panc-1 cells, compared with their respective controls, which presented with mainly full-length and inactivated caspase-3 and PARP proteins (Fig. 6j). Overall, our findings demonstrated that dysregulation of the miR-135b–BMAL1 axis confers GEM resistance to PC cells.

### YY1 forms a regulatory loop with miR-135b and BMAL1

**Fig. 7:**
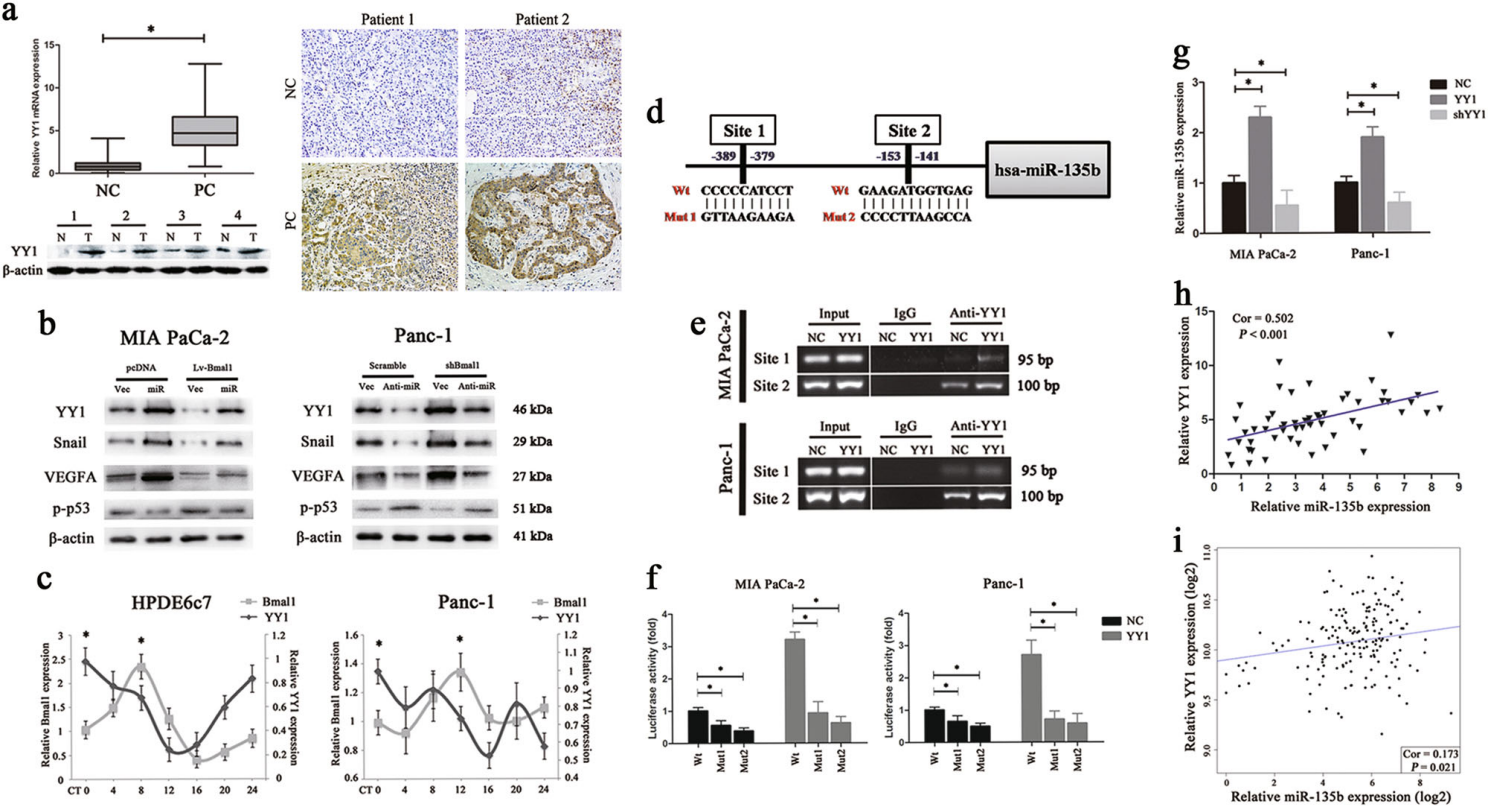
YY1 forms a feedback loop with miR-135b and BMAL1. **a** The mRNA and protein levels of YY1 in PC tissues and the paired normal pancreas (NC) from cohort 1 patients (left). The bars stand for minimums to maximums. IHC analysis of YY1 expression also confirmed a higher level of which in PC than in NC samples (right). Magnification ×200. **b** Western blot analysis showing the expressions of YY1 and its targets in MIA PaCa-2 or Panc-1 cells transfected with different vectors as indicated. **c** Quantitative RT-PCR analysis of YY1 and BMAL1 expressions in HPDE6c7 and Panc-1 cells at 4 h intervals over a 24-h period. *P < 0.05, peak vs. nadir. **d** Schematic diagram illustrating two putative YY1-binding sites and the respective mutants in the hsa-miR-135b promoter. **e** ChIP assay of MIA PaCa-2 and Panc-1 cells transfected with YY1 expression vectors or the controls (NCs). DNA samples collected from immunoprecipitates pull-downed by IgG or YY1 antibody were subjected to PCR analysis. The input was used as a positive control. **f** Relative luciferase activity of the wild-type or mutant putative binding sites of hsa-miR-135b promoter in MIA PaCa-2 and Panc-1 cells with (grey) or without (black) ectopic YY1 expression. **g** The level of miR-135b in PC cells transfected with YY1 expression vectors, shYY1 vectors or NCs was detected by qRT-PCR. U6 snRNA was used as an endogenous control. Data are presented as the means of at least three individual experiments ± SEM. *P < 0.05. **h**, **i** Correlation analysis of miR-135b and YY1 mRNA expressions in PC tissues from cohort 1 (55 cases, **h**) or TCGA cohort (178 cases, **i**)

We subsequently explored why miR-135b was aberrantly overexpressed in PC. On the basis of the IPA analysis, we observed that the transcription factor YY1, an important regulator of tumour initiation^28^, was negatively associated with BMAL1 expression. RT-PCR, western blotting and IHC assays in cohort 1 confirmed that YY1 expression was significantly higher in PC than in normal tissues (Fig. 7a). Moreover, the expression of YY1 and its targets Snail^29^ and VEGF^30^ aligned with that of miR-135b but was the opposite of BMAL1 expression. In contrast, p-p53^31^ demonstrated a different expression (Fig. 7b). Importantly, by referring to the CircaDB database (http://bioinf.itmat.upenn.edu/circa), we found that the *YY1* gene was rhythmically expressed in vivo. Temporal profiling of the *YY1* levels in normal and malignant pancreatic duct epithelial cells showed that the oscillation of *YY1* was marked by overt phase difference with that of *BMAL1* (Fig. 7c). These results suggested that YY1 may be a potential CCG.

On the other hand, genomic analysis identified two potential YY1-binding motifs inside the hsa-miR-135b promoter region (Fig. 7d). Therefore, we designed corresponding primer sets covering the two sites and performed a chromatin immunoprecipitation (ChIP) assay in PC cells with or without YY1 overexpression. As a result, YY1 protein was recruited to both binding sites, with the majority of YY1 bound to site 2 (Fig. 7e). Then, PGL3 vectors containing the wild-type or mutant putative binding sites of the miR-135b promoter were constructed and transfected into MIA PaCa-2 and Panc-1 cells. We found that ectopic YY1 expression significantly increased the luciferase activity of the wild-type miR-135b promoter; however, when mutations were present in binding site 1 or site 2, this enhancement was abrogated (Fig. 7f). These data indicated that YY1 activated miR-135b by directly binding to its promoter. Also, we found that the level of miR-135b was significantly upregulated by YY1 overexpression and was downregulated by YY1 silencing (Fig. 7g). In PC samples from two independent cohorts of patients, similar results were obtained showing that YY1 was positively correlated with miR-135b expression (cohort 1: 55 cases, Pearson *r* = 0.502, *P* < 0.001; Fig. 7h; TCGA cohort: 178 cases, Pearson *r* = 0.173, *P* = 0.021; Fig. 7i). Taken together, these findings suggested that YY1 transcriptionally activates miR-135b and forms a feedback loop with miR-135b–BMAL1 signalling.

### The miR-135b–BMAL1–YY1 loop holds predictive and prognostic values in patients with PC

**Fig. 8:**
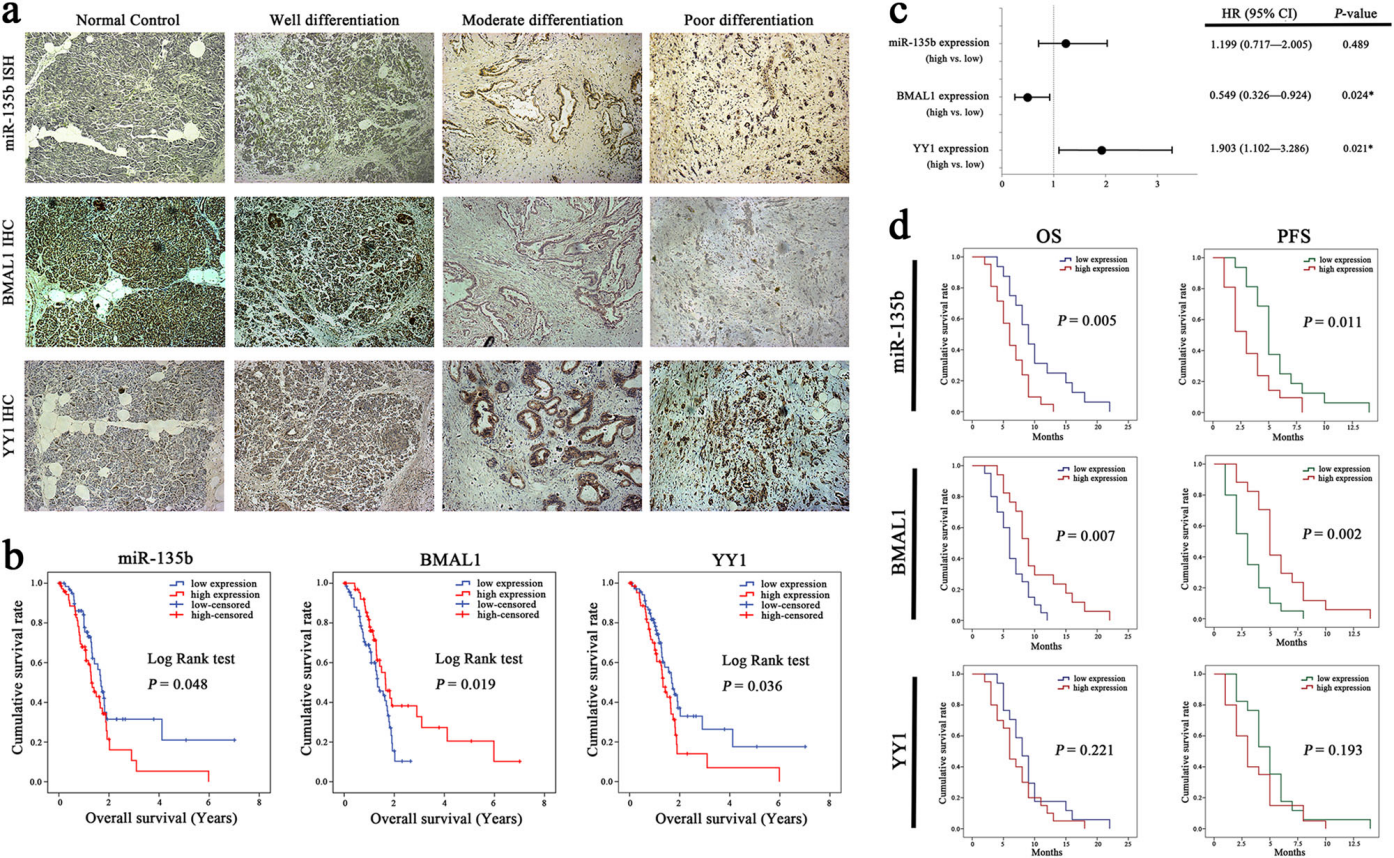
Clinical significance of the miR-135b–BMAL1–YY1 loop in human PC. **a** Representative ISH anaysis of miR-135b and IHC analyses of BMAL1 and YY1 in normal pancreas and in PC tissues with different histological grades. Magnification ×100. **b** Kaplan–Meier analysis of the correlation between miR-135b/BMAL1/YY1 expressions (mean split) and the OS times of 141 patients from TCGA cohort. **c** Multivariable analysis was performed in TCGA cohort. The bars correspond to 95% confidence interval (CI). **d** OS and progression-free survival (PFS) rates in 37 PC patients (cohort 2) received GEM or GEM-based regimens as first-line chemotherapy. Kaplan–Meier survival curves with log-rank test were used for prognostic evaluation in patients stratified by miR-135b/BMAL1/YY1 expression levels. *P < 0.05

We first evaluated the expression levels of miR-135b, BMAL1 and YY1 in the cohort 1 samples. In situ hybridisation (ISH) analysis showed that miR-135b staining was significantly stronger in PC tissues than in normal controls and was positively correlated with AJCC stage, T classification, histological grade and the level of CA19-9. Meanwhile, IHC analysis showed that BMAL1 was negatively associated with miR-135b expression and aggressive parameters of PC, whereas the YY1 level was positively related to pT stage and tumour differentiation (Fig. 8a; Supplementary Table S7). Next, we investigated the prognostic significance of miR-135b–BMAL1–YY1 using survival data from the TCGA PC cohort. The Kaplan–Meier analysis revealed that patients with high miR-135b/low BMAL1/ high YY1-expressing tumours had significantly shorter overall survival (OS) times (Fig. 8b; Supplementary Table S8). Multivariate Cox regression analysis adjusted for AJCC stage, T and N classifications, histological grade, tumour location, gender and age of patients consistently confirmed that the expression level of the miR-135b–BMAL1–YY1 loop was a prognostic indicator for OS in PC patients (Fig. 8c).

Next, we explored the relevance of miR-135b–BMAL1–YY1 expression and the tumour response to chemotherapy. Thirty-seven eligible patients (cohort 2) that had been diagnosed with advanced PC and that had received GEM or GEM-based regimens as first-line chemotherapy were enroled for investigation (Supplementary Table S9). The OS and progression-free survival (PFS) curves showed that high miR-135b and low BMAL1 expression were significantly correlated with poor survival outcomes (Fig. 8d). Moreover, GEM-based treatment resulted in an objective response rate (ORR) of 16.2% and a disease control rate (DCR) of 62.2%; high miR-135b/low BMAL1/high YY1 expression facilitated GEM resistance, whereas those patients with low miR-135b/high BMAL1/low YY1-expressing tumours had relatively higher chemosensitivity (Supplementary Table S10). Thus, on the basis of the above findings, we speculated that identifying miR-135b–BMAL1–YY1 signalling activity may be useful for predicting GEM responsiveness.

## Discussion

Although circadian disruption has been listed as an independent cancer risk factor^32^, its role and mechanism in spontaneous cancer initiation are not well understood. In this study, we unravelled a dysregulated pancreatic clockwork that is mediated by the miR-135b–BMAL1–YY1 loop during tumourigenesis and further we characterised the biological and clinical implications of this loop in PC.

First, our work revealed that the circadian network within malignant pancreatic duct epithelium is altered compared with that of normal pancreas, which suggested dysfunctional local circadian regulation in PC. As BMAL1 depletion is associated with uncontrolled cell division and proliferation^33^, we explored the mechanism by which BMAL1 is downregulated in PC, and we identified that hsa-miR-135b directly binds to the 3′-UTR of BMAL1. miR-135b serves as an oncomiR in various cancers;^34–37^ however, there is currently a lack of data on its role in PC. Here, we showed that miR-135b was significantly upregulated and was negatively correlated with BMAL1 expression in PC tissues. By targeting this core clock gene, miR-135b significantly enhanced PC cell proliferation and invasion. Notably, the pancreatic expression of miR-135b rhythmically oscillated and was roughly in anti-phase with that of *BMAL1*. Ectopic miR-135b expression impaired the operation of the pancreatic oscillator, and miR-135b downregulation is essential for cellular clock realignment. Thus, for the first time, we elucidated the role of a miRNA in the pancreatic oscillator. In addition, we identified YY1 as both an upstream activator of miR-135b and a downstream target of BMAL1. Thus, miR-135b, BMAL1 and YY1 form a feedback loop that modulates the pancreatic clockwork and, when desynchronized, may drive and exacerbate local circadian disturbance.

The circadian clock has been predicted as a pivotal tumour suppressor^38,39^, on major reason is that many cellular processes are closely intertwined with the central oscillator and are frequently out of circadian gating control in tumour cells^40–42^. Our bioinformatics analyses unravelled an altered network of intracellular biological pathways associated with miR-135b–BMAL1 misalignment. Strikingly, the gene signatures related to PC growth and metastasis were generally enriched in patients with high miR-135b and low BMAL1 expression. miR-135b upregulation markedly altered the expression of clock-controlled cell cycle checkpoints, DNA repair regulators and apoptotic mediators, resulting in tumourigenic transformation at both the molecular and cellular levels, whereas the restoration of BMAL1 partially reversed these changes. These observations clearly demonstrated the effects of miR-135b-mediated loss of circadian homoeostasis and gave convincing proof that a well-functioning time-keeping system is crucial for tumour suppression.

The extremely poor management of PC is mainly due to inadequate approach for early detection and a high degree of intrinsic and acquired resistance to chemotherapy^43^. As most patients are ineligible for initial resection, GEM-based regimens remain the cornerstone of PC treatment for the last two decades, but the rapid development of drug resistance within weeks severely limits the clinical benefit and survival extension in patients^27^. Therefore, the identification of targets that increase the potency of GEM is a major focus of ongoing research. Recent studies have proven the close involvement of the circadian timing system in the absorption, distribution, and metabolism of drugs; and drug efficiency and toxicity are changed when the host clock is altered^44,45^. BMAL1 has been reported to increase sensitivity to oxaliplatin and paclitaxel^33,46^. Here, we showed that overexpressing miR-135b significantly facilitated GEM resistance in PC cells, whereas BMAL1 upregulation restored GEM-induced apoptosis and sensitised the pancreatic xenograft tumours to GEM treatment. Our findings provide new insights into the mechanism of GEM resistance and suggest that genetic or pharmacological modulation of clock-related proteins may be a promising anti-tumour strategy.

Finally, we established the translational potential of the miR-135b–BMAL1–YY1 loop. High levels of miR-135b and YY1 are correlated with advanced TNM stage and poor histological differentiation, whereas low BMAL1 expression is linked to the aggressive features of PC. Patients with high miR-135b/low BMAL1/high YY1-expressing tumours presented with significantly shorter OS and PFS times and relatively unfavourable responses to GEM therapy. These findings pave the way for the future use of miR-135b–BMAL1–YY1 signalling as a predictive biomarker for PC inception and progression, as a prognostic factor for patient survival outcome, and as a therapeutic target for the modulation of GEM sensitivity.

In conclusion, this study identifies the miR-135b–BMAL1–YY1 loop as a determinant of pancreatic circadian homoeostasis, and we propose that targeting this signalling pathway may be useful for PC management.

## Materials and methods

### Cell culture, serum shock and cell transfection

Four human PC cell lines (MIA PaCa-2, AsPC-1, SW1990 and Panc-1) and HEK 293T cells were obtained from the American Type Culture Collection (ATCC). The immortalised human pancreatic duct epithelial cell line HPDE6c7 was acquired from Kyushu University, Japan. Cells were authenticated by short tandem repeat profiling and were cultured according to the manufacturer’s protocols. The cell lines were carefully checked for morphological consistency and for mycoplasma contamination using the Cycleave PCR Mycoplasma Detection Kit (TaKaRa). Prior to the experiments, the cells were serum-shocked with 50% horse serum (Gibco) for 2 h to achieve cellular synchronisation^47^. Cell transfection was performed with Lipofectamine 2000 reagent (Invitrogen) in Opti-MEM (Gibco), according to the manufacturer’s instructions. Oligonucleotides used in this study were all purchased from GenePharma (Shanghai, China).

### Vector construction and transduction

Luciferase constructs were generated by ligating oligonucleotides containing the wild-type or mutated putative target site of the BMAL1 3′-UTR into the pmirGLO vector (Promega) downstream of the luciferase gene; and the hsa-miR-135b promoter sequence with wild-type or mutated putative YY1-binding sites was amplified from human genomic DNA and cloned into the pGL3 vector (Promega). To generate miR-135b expression or knockdown (Anti-miR-135b) vectors, oligonucleotides encoding precursors or inhibitors of miR-135b were synthesised and subcloned into the *Bam*HI and *Xho*I restrictive sites of pPG/miR/EGFP/blasticidin plasmid or pGCMV/EGFP/miR/blasticidin plasmid (GenePharma), and then verified by DNA sequencing. Empty vectors were used as controls. The expression sequence of miR-135b was as follows: 5′-TATGGCTTTTCATTCCTATGTGA-3′. The silencing sequence of miR-135b was as follows: 5′-TCACATAGGAATGAAAAGCCATA-3′. Stably transfected cells were selected by blasticidin (Invitrogen), and the transfection efficiencies were examined by PCR. Lentiviral vectors encoding human BMAL1 (Lv-BMAL1) or YY1 or expressing short hairpin RNA against BMAL1 (shBMAL1) or YY1 and the pcDNA 3.1 empty vectors (pcDNA) and plasmids carrying scrambled shRNA (Scramble) were all constructed as previously described^19,29,48^.

### Luciferase reporter assay

Cells seeded in 96-well plates with a confluence of approximately 80% were transfected with luciferase reporter plasmids, pRL-TK-Renilla luciferase vectors (Promega), and the indicated RNA or YY1 expression constructs by Lipofectamine 2000 (Invitrogen). Forty-eight hours after transfection, firefly and Renilla luciferase activities were measured using a Dual-Luciferase Reporter Assay Kit (Promega).

### RNA isolation and quantitative real-time RT-PCR

Total RNA was extracted from cells or tissue specimens using TRIzol reagent (Invitrogen) and was then reverse-transcribed using a Transcriptor First Strand cDNA Synthesis Kit (Roche) for mRNAs or a One-Step Hairpin-it miRNAs qRT-PCR Quantification Kit (GenePharma) for miRNAs. The resulting cDNAs were used as templates for quantitative real-time PCR amplification. Relative RNA expression was evaluated using the comparative CT method and was normalised to U6 snRNA or *GAPDH*. Supplementary Table S11 shows the primer sequences used in this study.

### Western blotting

Cells were lysed in radioimmunoprecipitation assay (RIPA) buffer and proteins were then extracted and quantified using the bicinchoninic acid assay (BCA) kit (Beyotime Biotechnology, Jiangsu, China). Whole-cell lysates were separated by sodium dodecyl sulphate-polyacrylamide gel electrophoresis (SDS-PAGE) with 10% gels and were transferred to polyvinylidene difluoride membranes (Millipore). Membranes were incubated overnight with specific antibodies. Signals were visualised using ECL detection reagent (Beyotime). All antibodies were obtained from Abcam and Santa Cruz. β-actin was used as the internal control.

### Cell proliferation and viability assays

Cell proliferation was monitored using the Cell Counting Kit-8 (CCK-8) (Dojindo) according to the manufacturer’s instructions, and cell numbers were calculated based on relative absorbance at 450 nm. Colony formation assays were performed to assess long-term cell proliferation in vitro^19^.

To determine cell viability, cells were seeded in 96-well plates at an initial density of 3 × 10^3^ cells per well and incubated with gemcitabine (GEM) (Sigma-Aldrich, cat. no. 1288463) at various concentrations for 72 h. Then, 10 μL of CCK-8 solution was added into each well and incubated for 2 h before measurement. The IC_50_ value refers to the GEM concentration that produced 50% of the maximum cell death.

### Migration and invasion assays

In vitro wound-healing assays were performed to determine migration ability. Cells were cultured on 6-well plates forming a single cell layer, and then a straight scratch was made with a sterile pipette tip in the middle of the cell layer. Twenty-four hours later, cells that had migrated into the wound line were observed and the percent wound closure was calculated for five randomly chosen fields. Cell invasive ability was assessed by transwell assay in accordance with our previous report^19^.

### Flow cytometry

Cells seeded in 6-well culture plates were subjected to different doses of GEM treatment for 72 h. Then, cells were collected, washed with PBS, and stained with Annexin V-FITC/ PI (KeyGEN Biotech) in the dark for 15 min before assessment by flow cytometry (FACS Calibur, BD Biosciences).

### In vivo studies

Twenty-four male Balb/c nude mice (4–5-weeks old, 18–20 g) were purchased from the Laboratory Animal Center, Chinese Academy of Sciences (Shanghai, China). Mice were maintained under a 12/12 h light/dark cycles with lights turning on at 8:00 am and off at 8:00 pm. All animal-related procedures were performed according to the ethical guidelines of the Institutional Animal Care and Use Committee of Shanghai Jiao Tong University. Mice were randomly divided into eight groups (*n* = 3 per group). Equal amounts of the indicated cells (2.5 × 10^6^) were subcutaneously implanted into the right back-side of each mouse. GEM treatment began after the tumours reached a volume of ~100 mm^3^. GEM was intraperitoneally injected at a dose of 100 mg/kg at 9:00 am every 3 days for a total of six times. Tumour volume was monitored every 4 days. Mice were then killed, and the xenografts were excised and weighed.

### ISH and IHC analyses

ISH was performed on paraffin-embedded samples as previously described^49^. Sensitivity enhanced ISH kits (MK10301, Boster Biological Technology, Wuhan, China), and LNA-modified and DIG-labelled miR-135b probes (miRCURY LNA Detection probe, Exiqon) were used. The 5ʹ–3ʹ sequence was TCACATAGGAATGAAAAGCCATA, with digoxin labels at the 5ʹ and 3ʹ ends. Hybridisation, washing and scanning were carried out according to the manufacturer’s instructions. The intensity of miR-135b staining was scored according to the following standards: 0–1 (no staining), 1–2 (weak staining), 2–3 (medium staining) and 3–4 (strong staining). The percentage of miR-135b-expressing cells in three high-power fields of each individual sample was analysed. The expression scores were calculated as the intensity score × the percentage of positive cells. Individual samples were evaluated by two pathologists. The samples with expression scores greater than or equal to 2 were defined as high expression, and those with scores <2 had low expression. IHC analysis and the evaluation criteria were conducted according to previous methods^19^.

### ChIP assay

ChIP analysis was performed as described in our former report^19^. Briefly, MIA PaCa-2 and Panc-1 cells infected with YY1 expression vectors or the negative controls were cross-linked using 1% formaldehyde and lysed in SDS lysis buffer. Cell lysates were sonicated and then mixed with ChIP dilution buffer and protein A-Agarose/salmon sperm DNA (Millipore). After centrifugation, the supernatants were collected and divided into two parts: one part was for YY1 antibody detection (ab12132, Abcam), and the other part was for IgG reactivity. The next day, immune complexes were precipitated and rinsed. Immunoprecipitates were pelleted by centrifugation and incubated at 65 °C to reverse the protein-DNA cross-links. RNase A and proteinase K were then added, in turn, to recycle the DNA fragments. Purified DNA samples were dissolved in ddH_2_O and subjected to PCR analysis.

### Patients and samples

A total of 92 patients with pathologically diagnosed PC and who had undergone operations, laparoscopic biopsies, or EUS-FNAs at Shanghai General Hospital and Zhejiang Province People’s Hospital were enroled in this study. The patients were divided into two independent cohorts. Cohort 1 included 55 pairs of PC and control normal pancreatic (NC) tissues collected between 2014 and 2016, with both fresh and paraffin-embedded specimens; cohort 2 included 37 paraffin-embedded PC samples, which were obtained between 2009 and 2014 with full follow-up data. None of these patients had previously received anticancer therapy. The study protocol was approved by the ethics committee of Shanghai General Hospital and the ethics committee of Zhejiang Province People’s Hospital. All human materials were obtained with informed consent, and all research was carried out in accordance with the provisions of the Helsinki Declaration of 1975.

Follow-up and prognostic studies were conducted in cohort 2 PC patients who had been diagnosed with unresectable advanced PC and had received GEM or GEM-based regimens as first-line chemotherapy. A total of 37 patients were eligible for inclusion, and detailed information is provided in Supplementary Table S9. The criteria for enrolment were as follows: histologically confirmed advanced PC (AJCC Stage III/IV); no previous chemotherapy or other anti-tumour therapies; life expectancy ≥2 months; GEM-based regimens for at least two cycles; ECOG performance status <2; and adequate haematologic, hepatic, and renal function. Chemotherapy outcomes were evaluated by computed tomography or magnetic resonance imaging every two cycles. The objective tumour response for target lesions was classified as complete response (CR), partial response (PR), stable disease (SD) or progressive disease (PD), according to the Response Evaluation Criteria in Solid Tumour (RECIST) version 1.1. The endpoints included ORR, DCR, OS and PFS. The OS and PFS times were calculated from the date that first-line chemotherapy treatment was started to the date of mortality and disease progression, respectively.

### Bioinformatics analysis

Three independent public data sets of human genome arrays for PC and normal pancreatic tissues were downloaded from GEO (http://www.ncbi.nlm.nih.gov/geo/). The GSE19650 data set consisted of 15 PC and 7 NC, GSE32676 consisted of 25 PC and 7 NC, and GSE16515 consisted of 36 PC and 16 NC. The GEO-developed R-based interactive tool GEO_2_R (http://www.ncbi.nlm.nih.gov/geo/info/geo2r/) was used to compare expression profiles between samples. The expression values of specific genes were extracted from the original submitter-supplied files. The data were normalised to the average expression of the NC samples in the same probeset. TCGA RNA-seq data and corresponding clinical data from 178 PC patients were downloaded from the TCGA database (https://tcga-data.nci.nih.gov/tcga/) following approval of this project by the consortium. GSEA was performed to gain insight into the biological pathways involved in PC pathogenesis through the miR-135b–BMAL1 axis. Gene sets with FDRs of 0.25, a well-established cutoff value for the identification of biologically relevant genes, were considered to be enriched between the classes being compared. The gene sets collection (C2.CP: KEGG v5.2) from the Molecular Signatures Database-MsigDB (http://www.broad.mit.edu/gsea/msigdb/index.jsp) were used for enrichment analysis. Moreover, the profiling data from the GSE19650 cohort were uploaded (http://www.ingenuity.com) and used to conduct an ingenuity pathway analysis (IPA) to map the lists of BMAL1-associated differentially expressed genes between PC and normal pancreatic tissue. The ingenuity ‘core analysis’ typically generates canonical pathways and functional molecular networks pertaining to the data set in question based on the Ingenuity Pathway Knowledge Base (IPKB), which is derived from known functions and interactions of genes published in the literature.

### Statistical analysis

Statistical analyses were performed using the programme R (www.r-project.org) and SPSS version 19.0 software. Data are presented as the means ± SEM of at least three biological repeats. Comparisons between groups were analysed with Student’s *t*-test, one-way ANOVA, and *χ*^2^ tests. A *P*-value < 0.05 was considered to be statistically significant. Pearson Correlation analysis was used to determine the relationship between the expression levels of miR-135b, BMAL1 and YY1. OS and DFS curves were plotted according to the Kaplan–Meier method, and the log-rank test was used for comparison. Prognostic factors and survival data were further evaluated by the multivariate Cox regression analysis. Hazard ratios and 95% confidence intervals were derived from Cox’s proportional hazards model. The rhythms of clock-related genes and miR-135b were analysed using the JTK_CYCLE nonparametric algorithm, which is a highly efficient tool for characterising circadian oscillations and cycling variables^25^.

## Electronic supplementary material

Supplementary Information
