# Supplementary material for: The MiR-135b–BMAL1–YY1 loop disturbs pancreatic clockwork to promote tumourigenesis and chemoresistance
Source: Cell Death Dis. 2018 Feb 2;9(2):149. doi: 10.1038/s41419-017-0233-y (PMC5833454; doi:10.1038/s41419-017-0233-y)
Supplement: Supplementary file 1 — Supplementary Information [file 41419_2017_233_MOESM1_ESM.docx]

**Supplementary Table S1. Expression of the clock genes in the GSE19650 dataset**

| BMAL1 |  |  |  |
| --- | --- | --- | --- |
| Sample | Title | FC(209824_s_at) | FC(210971_s_at) |
| GSM490138 | normal main pancreatic duct 1 | 0.970513318 | 1.458004965 |
| GSM490139 | normal main pancreatic duct 2 | 1.436610339 | 1.437514084 |
| GSM490140 | normal main pancreatic duct 3 | 0.658521922 | 0.363844482 |
| GSM490141 | normal main pancreatic duct 4 | 0.394584969 | 1.668430546 |
| GSM490142 | normal main pancreatic duct 5 | 0.353521205 | 0.444231783 |
| GSM490143 | normal main pancreatic duct 6 | 1.913581749 | 1.281730851 |
| GSM490144 | normal main pancreatic duct 7 | 1.272665981 | 0.346243341 |
| GSM490145 | IPMA 1 | 0.220232283 | 0.211739099 |
| GSM490146 | IPMA 2 | 0.27641663 | 0.26874578 |
| GSM490147 | IPMA 3 | 0.118634413 | 0.937326435 |
| GSM490148 | IPMA 4 | 0.331306406 | 0.431884714 |
| GSM490149 | IPMA 5 | 0.464388189 | 0.67041958 |
| GSM490150 | IPMA 6 | 0.456827899 | 0.617616157 |
| GSM490151 | IPMC 1 | 0.376202073 | 0.141334535 |
| GSM490152 | IPMC 2 | 0.661732456 | 0.425842531 |
| GSM490153 | IPMC 3 | 0.478058849 | 0.439765822 |
| GSM490154 | IPMC 4 | 1.276808605 | 0.793627567 |
| GSM490155 | IPMC 5 | 0.554438485 | 0.473654586 |
| GSM490156 | IPMC 6 | 0.643297778 | 0.459993999 |
| GSM490157 | Invasive cancer originating in IPMN 1 | 0.825935728 | 0.42137657 |
| GSM490158 | Invasive cancer originating in IPMN 2 | 0.475366143 | 0.116903101 |
| GSM490159 | Invasive cancer originating in IPMN 3 | 0.576756874 | 0.360692039 |

| CLOCK |  |  |  |  |  |
| --- | --- | --- | --- | --- | --- |
| Sample | Title | FC(204980_at) | FC(217563_at) | FC(225856_at) | FC(227531_at) |
| GSM490138 | normal main pancreatic duct 1 | 1.224174981 | 0.331676309 | 1.181301277 | 1.335280659 |
| GSM490139 | normal main pancreatic duct 2 | 1.397637275 | 0.936541364 | 1.430629935 | 1.182868766 |
| GSM490140 | normal main pancreatic duct 3 | 0.205529378 | 1.009835905 | 1.242492957 | 0.947888488 |
| GSM490141 | normal main pancreatic duct 4 | 0.719915403 | 1.33781047 | 0.636612999 | 1.00219848 |
| GSM490142 | normal main pancreatic duct 5 | 1.018833129 | 1.459968039 | 0.744007143 | 0.733428502 |
| GSM490143 | normal main pancreatic duct 6 | 1.267868726 | 0.631517615 | 0.86724801 | 0.879162703 |
| GSM490144 | normal main pancreatic duct 7 | 1.166041671 | 1.292649187 | 0.89770665 | 0.919171542 |
| GSM490145 | IPMA 1 | 2.321206787 | 0.258751942 | 1.14988223 | 1.140251901 |
| GSM490146 | IPMA 2 | 1.466084558 | 0.890269557 | 1.013401458 | 0.804848292 |
| GSM490147 | IPMA 3 | 1.905272335 | 0.624114126 | 0.835966165 | 1.237952568 |
| GSM490148 | IPMA 4 | 1.534156789 | 0.580433541 | 0.777964409 | 0.868959876 |
| GSM490149 | IPMA 5 | 1.684553286 | 0.434954981 | 0.691184728 | 1.081700284 |
| GSM490150 | IPMA 6 | 1.758063793 | 0.640031628 | 1.211073911 | 1.715278648 |
| GSM490151 | IPMC 1 | 1.861391063 | 0.471602252 | 1.1842854 | 1.027648228 |
| GSM490152 | IPMC 2 | 1.851639669 | 0.652617559 | 1.056379696 | 1.25311351 |
| GSM490153 | IPMC 3 | 2.025101963 | 0.605605404 | 0.776283696 | 0.881942687 |
| GSM490154 | IPMC 4 | 1.9302134 | 0.279111537 | 1.427165608 | 1.483880823 |
| GSM490155 | IPMC 5 | 1.682115437 | 0.801057514 | 0.880762317 | 0.942414499 |
| GSM490156 | IPMC 6 | 1.103032665 | 0.862506473 | 0.822932062 | 0.958377911 |
| GSM490157 | Invasive cancer originating in IPMN 1 | 2.590682806 | 0.608196625 | 1.041973583 | 1.06662532 |
| GSM490158 | Invasive cancer originating in IPMN 2 | 2.407281591 | 0.497514464 | 1.001293463 | 0.842908276 |
| GSM490159 | Invasive cancer originating in IPMN 3 | 1.299560756 | 0.345372764 | 0.799470678 | 0.813761436 |

| PER1 |  |  |  |  |  |
| --- | --- | --- | --- | --- | --- |
| Sample | Title | FC(202861_at) | FC(244677_at) | FC(242832_at) | FC(36829_at) |
| GSM490138 | normal main pancreatic duct 1 | 0.331669457 | 0.204151953 | 0.247761194 | 0.228154397 |
| GSM490139 | normal main pancreatic duct 2 | 0.809969399 | 0.484787346 | 1.164179104 | 0.715460745 |
| GSM490140 | normal main pancreatic duct 3 | 0.788442952 | 1.00056029 | 0.635820896 | 0.664016531 |
| GSM490141 | normal main pancreatic duct 4 | 1.860636903 | 1.823835888 | 1.698507463 | 2.667479016 |
| GSM490142 | normal main pancreatic duct 5 | 1.671764453 | 1.168039414 | 0.749253731 | 1.249901301 |
| GSM490143 | normal main pancreatic duct 6 | 0.772814163 | 0.900739595 | 1.710447761 | 0.695565764 |
| GSM490144 | normal main pancreatic duct 7 | 0.764704885 | 1.417885319 | 0.794029851 | 0.779421211 |
| GSM490145 | IPMA 1 | 0.967436827 | 0.603826957 | 1.104477612 | 0.66291317 |
| GSM490146 | IPMA 2 | 0.705580879 | 0.428307267 | 0.256716418 | 0.396106652 |
| GSM490147 | IPMA 3 | 0.647194079 | 1.105872104 | 1.934328358 | 0.514545576 |
| GSM490148 | IPMA 4 | 0.303950471 | 0.562055232 | 0.731343284 | 0.337525071 |
| GSM490149 | IPMA 5 | 0.701378798 | 1.039390345 | 0.695522388 | 0.588539733 |
| GSM490150 | IPMA 6 | 0.266131749 | 0.596963058 | 1.110447761 | 0.493754114 |
| GSM490151 | IPMC 1 | 0.059861213 | 0.06079453 | 0.805970149 | 0.200639329 |
| GSM490152 | IPMC 2 | 0.060156096 | 0.292009853 | 4.704477612 | 0.235808965 |
| GSM490153 | IPMC 3 | 0.587922632 | 0.820922261 | 3.919402985 | 0.294873267 |
| GSM490154 | IPMC 4 | 0.729392667 | 0.491259021 | 0.674626866 | 0.735666046 |
| GSM490155 | IPMC 5 | 0.409002478 | 0.166106344 | 0.453731343 | 0.411691628 |
| GSM490156 | IPMC 6 | 0.261487345 | 0.645402571 | 1.089552239 | 0.386762563 |
| GSM490157 | Invasive cancer originating in IPMN 1 | 0.188798728 | 0.266515373 | 0.468656716 | 0.214810624 |
| GSM490158 | Invasive cancer originating in IPMN 2 | 0.17538156 | 0.250630351 | 0.958208955 | 0.296011108 |
| GSM490159 | Invasive cancer originating in IPMN 3 | 0.387623473 | 1.192553337 | 0.829850746 | 0.572713396 |

| PER2 |  |  |  |
| --- | --- | --- | --- |
| Sample | Title | FC(208518_s_at) | FC(205251_at) |
| GSM490138 | normal main pancreatic duct 1 | 0.698027713 | 0.823760182 |
| GSM490139 | normal main pancreatic duct 2 | 2.225560962 | 1.13465185 |
| GSM490140 | normal main pancreatic duct 3 | 1.272346748 | 1.148007578 |
| GSM490141 | normal main pancreatic duct 4 | 0.591052576 | 0.763316952 |
| GSM490142 | normal main pancreatic duct 5 | 0.721932772 | 1.108924951 |
| GSM490143 | normal main pancreatic duct 6 | 1.382907644 | 1.21582785 |
| GSM490144 | normal main pancreatic duct 7 | 0.10817039 | 0.805510208 |
| GSM490145 | IPMA 1 | 2.175957965 | 1.820459919 |
| GSM490146 | IPMA 2 | 2.85844739 | 1.918758648 |
| GSM490147 | IPMA 3 | 4.246136046 | 2.351121803 |
| GSM490148 | IPMA 4 | 1.127123516 | 1.235333492 |
| GSM490149 | IPMA 5 | 2.126952595 | 0.816040686 |
| GSM490150 | IPMA 6 | 1.311790094 | 0.765528638 |
| GSM490151 | IPMC 1 | 1.633313134 | 0.90619185 |
| GSM490152 | IPMC 2 | 0.71715176 | 0.982188225 |
| GSM490153 | IPMC 3 | 0.515154014 | 0.618744051 |
| GSM490154 | IPMC 4 | 2.162212557 | 0.740015773 |
| GSM490155 | IPMC 5 | 2.198667771 | 0.989137199 |
| GSM490156 | IPMC 6 | 2.471783066 | 1.278340077 |
| GSM490157 | Invasive cancer originating in IPMN 1 | 0.705199231 | 1.68568978 |
| GSM490158 | Invasive cancer originating in IPMN 2 | 0.284470198 | 0.549211506 |
| GSM490159 | Invasive cancer originating in IPMN 3 | 0.155382881 | 2.015402179 |

| CRY1 |  |  |
| --- | --- | --- |
| Sample | Title | FC(209674_at) |
| GSM490138 | normal main pancreatic duct 1 | 1.24192583 |
| GSM490139 | normal main pancreatic duct 2 | 1.630360114 |
| GSM490140 | normal main pancreatic duct 3 | 0.716559525 |
| GSM490141 | normal main pancreatic duct 4 | 0.260396152 |
| GSM490142 | normal main pancreatic duct 5 | 0.933621258 |
| GSM490143 | normal main pancreatic duct 6 | 1.464882942 |
| GSM490144 | normal main pancreatic duct 7 | 0.752253669 |
| GSM490145 | IPMA 1 | 0.382369498 |
| GSM490146 | IPMA 2 | 0.595772031 |
| GSM490147 | IPMA 3 | 0.411626161 |
| GSM490148 | IPMA 4 | 0.656335185 |
| GSM490149 | IPMA 5 | 0.918510116 |
| GSM490150 | IPMA 6 | 0.371646008 |
| GSM490151 | IPMC 1 | 0.666143875 |
| GSM490152 | IPMC 2 | 0.705632746 |
| GSM490153 | IPMC 3 | 0.464481277 |
| GSM490154 | IPMC 4 | 0.949918252 |
| GSM490155 | IPMC 5 | 0.351164651 |
| GSM490156 | IPMC 6 | 0.644425844 |
| GSM490157 | Invasive cancer originating in IPMN 1 | 0.792826748 |
| GSM490158 | Invasive cancer originating in IPMN 2 | 0.711274013 |
| GSM490159 | Invasive cancer originating in IPMN 3 | 0.743884943 |

| CRY2 |  |  |
| --- | --- | --- |
| Sample | Title | FC(212695_at) |
| GSM490138 | normal main pancreatic duct 1 | 0.772509877 |
| GSM490139 | normal main pancreatic duct 2 | 0.152214598 |
| GSM490140 | normal main pancreatic duct 3 | 0.216677064 |
| GSM490141 | normal main pancreatic duct 4 | 2.493033895 |
| GSM490142 | normal main pancreatic duct 5 | 1.359950094 |
| GSM490143 | normal main pancreatic duct 6 | 0.26783115 |
| GSM490144 | normal main pancreatic duct 7 | 1.737783323 |
| GSM490145 | IPMA 1 | 3.145560408 |
| GSM490146 | IPMA 2 | 2.424620503 |
| GSM490147 | IPMA 3 | 2.546475359 |
| GSM490148 | IPMA 4 | 0.530047827 |
| GSM490149 | IPMA 5 | 1.54294032 |
| GSM490150 | IPMA 6 | 0.625493866 |
| GSM490151 | IPMC 1 | 0.545227698 |
| GSM490152 | IPMC 2 | 0.209399043 |
| GSM490153 | IPMC 3 | 0.170097733 |
| GSM490154 | IPMC 4 | 1.011852776 |
| GSM490155 | IPMC 5 | 2.522769807 |
| GSM490156 | IPMC 6 | 0.116240383 |
| GSM490157 | Invasive cancer originating in IPMN 1 | 0.521938033 |
| GSM490158 | Invasive cancer originating in IPMN 2 | 0.22249948 |
| GSM490159 | Invasive cancer originating in IPMN 3 | 0.863589104 |

| DBP |  |  |  |
| --- | --- | --- | --- |
| Sample | Title | FC(209782_s_at) | FC(209783_at) |
| GSM490138 | normal main pancreatic duct 1 | 0.675085512 | 0.868537469 |
| GSM490139 | normal main pancreatic duct 2 | 0.439356575 | 1.329848579 |
| GSM490140 | normal main pancreatic duct 3 | 0.925188164 | 0.17179862 |
| GSM490141 | normal main pancreatic duct 4 | 1.448391414 | 0.497102489 |
| GSM490142 | normal main pancreatic duct 5 | 2.176659673 | 2.049652983 |
| GSM490143 | normal main pancreatic duct 6 | 0.143258033 | 1.549369038 |
| GSM490144 | normal main pancreatic duct 7 | 1.192060151 | 0.533689232 |
| GSM490145 | IPMA 1 | 2.387953293 | 1.247926088 |
| GSM490146 | IPMA 2 | 1.014784325 | 1.177138694 |
| GSM490147 | IPMA 3 | 1.480971836 | 3.970457 |
| GSM490148 | IPMA 4 | 1.486721322 | 1.611407428 |
| GSM490149 | IPMA 5 | 0.485352465 | 1.768094133 |
| GSM490150 | IPMA 6 | 0.593155332 | 0.413589271 |
| GSM490151 | IPMC 1 | 0.261601625 | 1.756959037 |
| GSM490152 | IPMC 2 | 0.297535914 | 1.53187103 |
| GSM490153 | IPMC 3 | 1.018138192 | 2.680376621 |
| GSM490154 | IPMC 4 | 0.147570147 | 0.82876927 |
| GSM490155 | IPMC 5 | 1.482409207 | 1.482558463 |
| GSM490156 | IPMC 6 | 1.041615261 | 0.328485325 |
| GSM490157 | Invasive cancer originating in IPMN 1 | 0.469541378 | 1.620156432 |
| GSM490158 | Invasive cancer originating in IPMN 2 | 1.675975245 | 2.015452331 |
| GSM490159 | Invasive cancer originating in IPMN 3 | 1.454620024 | 1.504828655 |

| RORA |  |  |  |  |  |  |  |  |  |
| --- | --- | --- | --- | --- | --- | --- | --- | --- | --- |
| Sample | Title | FC(210479_s_at) | FC(235567_at) | FC(236266_at) | FC(239550_at) | FC(241760_x_at) | FC(240951_at) | FC(210426_x_at) | FC(226682_at) |
| GSM490138 | normal main pancreatic duct 1 | 0.861660019 | 1.000662615 | 0.686283293 | 0.370501088 | 0.92 | 0.838660578 | 0.611345103 | 1.116643216 |
| GSM490139 | normal main pancreatic duct 2 | 1.224638177 | 1.758330274 | 1.240952669 | 0.865051428 | 0.218333333 | 0.678843227 | 1.463517268 | 1.081992802 |
| GSM490140 | normal main pancreatic duct 3 | 0.971071129 | 1.289054013 | 1.050210035 | 0.96549839 | 2.228333333 | 1.657534247 | 0.976534134 | 1.159712334 |
| GSM490141 | normal main pancreatic duct 4 | 0.716509701 | 0.706394482 | 1.304133352 | 2.67172451 | 1.98 | 1.385083714 | 0.929622281 | 0.321592843 |
| GSM490142 | normal main pancreatic duct 5 | 0.918076494 | 0.499792158 | 0.789533437 | 0.467380261 | 1.058333333 | 1.347031963 | 0.81359897 | 0.921070227 |
| GSM490143 | normal main pancreatic duct 6 | 1.234231025 | 1.073771371 | 1.453605944 | 0.579353923 | 0.48 | 0.290715373 | 1.184834908 | 1.268230365 |
| GSM490144 | normal main pancreatic duct 7 | 1.07381404 | 0.671996705 | 0.47528082 | 1.08049095 | 0.12 | 0.802130898 | 1.020546784 | 1.13075569 |
| GSM490145 | IPMA 1 | 0.477156446 | 0.171180162 | 0.681180815 | 0.626832951 | 0.115 | 0.200913242 | 0.362538332 | 0.199071672 |
| GSM490146 | IPMA 2 | 0.415621836 | 0.816866341 | 0.657169154 | 0.1097781 | 0.696666667 | 0.217656012 | 0.349671396 | 0.347277882 |
| GSM490147 | IPMA 3 | 0.106135504 | 0.158617964 | 0.203498829 | 0.089194706 | 1.25 | 2.152207002 | 0.118756844 | 0.054809889 |
| GSM490148 | IPMA 4 | 0.40017969 | 0.389859449 | 0.59969124 | 0.209127281 | 0.145 | 0.203957382 | 0.411631489 | 0.364434883 |
| GSM490149 | IPMA 5 | 0.216599487 | 0.178243028 | 0.147071425 | 0.392731153 | 1.74 | 0.143074581 | 0.200569569 | 0.178929016 |
| GSM490150 | IPMA 6 | 0.052117241 | 0.087018829 | 0.040969897 | 0.122128136 | 0.48 | 0.181126332 | 0.077450116 | 0.024322572 |
| GSM490151 | IPMC 1 | 0.010207024 | 0.026148781 | 0.225709616 | 0.311220914 | 1.856666667 | 1.470319635 | 0.043929816 | 0.016736486 |
| GSM490152 | IPMC 2 | 0.043080076 | 0.089714579 | 0.015007288 | 0.391358927 | 0.96 | 0.916286149 | 0.043488033 | 0.019335267 |
| GSM490153 | IPMC 3 | 0.21999208 | 0.386624548 | 0.658069591 | 0.379832226 | 0.675 | 0.228310502 | 0.223349015 | 0.174135148 |
| GSM490154 | IPMC 4 | 0.196126946 | 0.284779091 | 0.497341534 | 0.270054126 | 0.075 | 1.083713851 | 0.246708301 | 0.113724003 |
| GSM490155 | IPMC 5 | 0.075368199 | 0.327425866 | 0.113004881 | 0.352113256 | 0.35 | 1.140030441 | 0.081564222 | 0.045819285 |
| GSM490156 | IPMC 6 | 0.604261681 | 0.623904514 | 1.023046843 | 0.465459144 | 0.146666667 | 0.528158295 | 0.574621875 | 0.301214699 |
| GSM490157 | Invasive cancer originating in IPMN 1 | 0.250408427 | 0.216684432 | 0.729204138 | 0.1097781 | 0.713333333 | 0.133942161 | 0.264352017 | 0.154328905 |
| GSM490158 | Invasive cancer originating in IPMN 2 | 0.112131034 | 0.126915937 | 0.347868943 | 0.112522553 | 0.79 | 0.726027397 | 0.061076526 | 0.07510393 |
| GSM490159 | Invasive cancer originating in IPMN 3 | 0.369178414 | 0.574464449 | 1.089979348 | 0.826903539 | 0.135 | 0.161339422 | 0.402492099 | 0.536484278 |

**Supplementary Table S2. Expression of the clock genes in the GSE32676 dataset**

| BMAL1 |  |  |  |
| --- | --- | --- | --- |
| Sample | Title | FC(209824_s_at) | FC(210971_s_at) |
| GSM811004 | human PDAC 1 (mRNA) | 0.422209575 | 0.8288244 |
| GSM811005 | human PDAC 2 (mRNA) | 1.181132816 | 1.815174669 |
| GSM811006 | human PDAC 3 (mRNA) | 0.216539362 | 0.82746396 |
| GSM811007 | human PDAC 4 (mRNA) | 0.397694138 | 0.828778442 |
| GSM811008 | human PDAC 5 (mRNA) | 0.79859791 | 0.847055486 |
| GSM811009 | human PDAC 6 (mRNA) | 0.358799852 | 0.580645037 |
| GSM811010 | human PDAC 7 (mRNA) | 1.185948368 | 1.027200907 |
| GSM811011 | human PDAC 8 (mRNA) | 0.643990451 | 0.845770638 |
| GSM811012 | human PDAC 9 (mRNA) | 0.663418524 | 0.82746396 |
| GSM811013 | human PDAC 10 (mRNA) | 0.781198563 | 0.837980386 |
| GSM811014 | human PDAC 11 (mRNA) | 0.802343099 | 1.157681489 |
| GSM811015 | human PDAC 12 (mRNA) | 0.422209575 | 0.828175473 |
| GSM811016 | human PDAC 13 (mRNA) | 1.073067776 | 1.376637182 |
| GSM811017 | human PDAC 14 (mRNA) | 0.672828723 | 0.82746396 |
| GSM811018 | human PDAC 15 (mRNA) | 1.24220321 | 0.967825181 |
| GSM811019 | human PDAC 16 (mRNA) | 0.771127435 | 0.8288244 |
| GSM811020 | human PDAC 17 (mRNA) | 0.756892964 | 0.828841635 |
| GSM811021 | human PDAC 18 (mRNA) | 0.422209575 | 0.799754096 |
| GSM811022 | human PDAC 19 (mRNA) | 0.71402733 | 0.851269816 |
| GSM811023 | human PDAC 20 (mRNA) | 1.558146842 | 1.013024019 |
| GSM811024 | human PDAC 21 (mRNA) | 0.866109418 | 0.827515581 |
| GSM811025 | human PDAC 22 (mRNA) | 0.583041791 | 0.8288244 |
| GSM811026 | human PDAC 23 (mRNA) | 0.839749654 | 0.910138735 |
| GSM811027 | human PDAC 24 (mRNA) | 0.365362685 | 0.786690671 |
| GSM811028 | human PDAC 25 (mRNA) | 0.434856469 | 0.827515581 |
| GSM811029 | non-malignant pancreas sample 1 (mRNA) | 0.434856469 | 0.827515581 |
| GSM811030 | non-malignant pancreas sample 2 (mRNA) | 1.584481924 | 1.344396211 |
| GSM811031 | non-malignant pancreas sample 3 (mRNA) | 1.28075205 | 0.892279983 |
| GSM811032 | non-malignant pancreas sample 4 (mRNA) | 0.723954635 | 0.911293942 |
| GSM811033 | non-malignant pancreas sample 5 (mRNA) | 1.162992023 | 1.258467868 |
| GSM811034 | non-malignant pancreas sample 6 (mRNA) | 0.871022083 | 0.951356774 |
| GSM811035 | non-malignant pancreas sample 7 (mRNA) | 0.941942697 | 0.82746396 |

| CLOCK |  |  |  |  |
| --- | --- | --- | --- | --- |
| Sample | Title | FC(204980_at) | FC(225856_at) | FC(227531_at) |
| GSM811004 | human PDAC 1 (mRNA) | 1.594724675 | 0.836713395 | 0.320401944 |
| GSM811005 | human PDAC 2 (mRNA) | 0.853496634 | 0.804997069 | 0.784222429 |
| GSM811006 | human PDAC 3 (mRNA) | 1.180982321 | 1.280471761 | 1.064890293 |
| GSM811007 | human PDAC 4 (mRNA) | 1.660349972 | 1.774712631 | 1.689498194 |
| GSM811008 | human PDAC 5 (mRNA) | 1.068700329 | 0.954697401 | 0.993502022 |
| GSM811009 | human PDAC 6 (mRNA) | 1.115861033 | 0.606763176 | 0.728552483 |
| GSM811010 | human PDAC 7 (mRNA) | 1.664221392 | 1.347799071 | 1.251028632 |
| GSM811011 | human PDAC 8 (mRNA) | 1.281121556 | 1.571350954 | 1.482153438 |
| GSM811012 | human PDAC 9 (mRNA) | 0.760625055 | 0.535105564 | 0.684667202 |
| GSM811013 | human PDAC 10 (mRNA) | 1.170291048 | 1.145839865 | 1.235534083 |
| GSM811014 | human PDAC 11 (mRNA) | 0.965727033 | 0.846343624 | 0.748380985 |
| GSM811015 | human PDAC 12 (mRNA) | 1.115861033 | 0.595802233 | 0.665811091 |
| GSM811016 | human PDAC 13 (mRNA) | 2.088688527 | 1.585684155 | 1.957883345 |
| GSM811017 | human PDAC 14 (mRNA) | 1.115861033 | 0.863630103 | 0.97887382 |
| GSM811018 | human PDAC 15 (mRNA) | 0.940700495 | 1.032624288 | 1.173310464 |
| GSM811019 | human PDAC 16 (mRNA) | 1.060582882 | 0.956744386 | 1.115580644 |
| GSM811020 | human PDAC 17 (mRNA) | 1.955811551 | 1.752962868 | 1.166231995 |
| GSM811021 | human PDAC 18 (mRNA) | 1.074002512 | 0.872305527 | 0.952139462 |
| GSM811022 | human PDAC 19 (mRNA) | 1.99064694 | 2.108556144 | 1.965634124 |
| GSM811023 | human PDAC 20 (mRNA) | 0.871182795 | 1.092797006 | 1.002814532 |
| GSM811024 | human PDAC 21 (mRNA) | 1.143580059 | 1.127428104 | 1.179385047 |
| GSM811025 | human PDAC 22 (mRNA) | 0.88041606 | 0.590150751 | 0.60350382 |
| GSM811026 | human PDAC 23 (mRNA) | 1.437585107 | 1.319805593 | 1.231421624 |
| GSM811027 | human PDAC 24 (mRNA) | 0.987619597 | 0.889135645 | 0.913378546 |
| GSM811028 | human PDAC 25 (mRNA) | 1.40222452 | 1.012004311 | 0.639020805 |
| GSM811029 | non-malignant pancreas sample 1 (mRNA) | 0.867200425 | 0.718427976 | 0.647640364 |
| GSM811030 | non-malignant pancreas sample 2 (mRNA) | 1.689512902 | 1.650594155 | 1.706327778 |
| GSM811031 | non-malignant pancreas sample 3 (mRNA) | 0.659382438 | 0.739091781 | 0.612216914 |
| GSM811032 | non-malignant pancreas sample 4 (mRNA) | 0.609432088 | 0.863139372 | 0.917159661 |
| GSM811033 | non-malignant pancreas sample 5 (mRNA) | 0.975029226 | 0.747189163 | 0.811754177 |
| GSM811034 | non-malignant pancreas sample 6 (mRNA) | 1.115861033 | 1.283519697 | 1.375975525 |
| GSM811035 | non-malignant pancreas sample 7 (mRNA) | 1.083581275 | 0.998037159 | 0.928925504 |

| PER1 |  |  |  |  |
| --- | --- | --- | --- | --- |
| Sample | Title | FC(202861_at) | FC(36829_at) | FC(244677_at) |
| GSM811004 | human PDAC 1 (mRNA) | 0.467642395 | 0.426984655 | 0.395810118 |
| GSM811005 | human PDAC 2 (mRNA) | 0.204811358 | 0.289813384 | 0.185493482 |
| GSM811006 | human PDAC 3 (mRNA) | 0.134205679 | 0.153295381 | 0.096816074 |
| GSM811007 | human PDAC 4 (mRNA) | 0.229195024 | 0.275509912 | 0.226388157 |
| GSM811008 | human PDAC 5 (mRNA) | 0.460124962 | 0.417479172 | 0.330057754 |
| GSM811009 | human PDAC 6 (mRNA) | 0.432300866 | 0.424258694 | 0.749089703 |
| GSM811010 | human PDAC 7 (mRNA) | 0.252516161 | 0.303493207 | 0.245470302 |
| GSM811011 | human PDAC 8 (mRNA) | 0.217447289 | 0.271899637 | 0.171191194 |
| GSM811012 | human PDAC 9 (mRNA) | 0.495304499 | 0.483479334 | 0.519415293 |
| GSM811013 | human PDAC 10 (mRNA) | 0.217409612 | 0.184442896 | 0.096816074 |
| GSM811014 | human PDAC 11 (mRNA) | 0.539325305 | 0.640706765 | 0.407914981 |
| GSM811015 | human PDAC 12 (mRNA) | 0.396812019 | 0.54402548 | 0.453753412 |
| GSM811016 | human PDAC 13 (mRNA) | 0.248291647 | 0.286344962 | 0.47404647 |
| GSM811017 | human PDAC 14 (mRNA) | 0.412006108 | 0.471564209 | 0.211645306 |
| GSM811018 | human PDAC 15 (mRNA) | 0.349667988 | 0.391785559 | 0.354648167 |
| GSM811019 | human PDAC 16 (mRNA) | 0.667670235 | 0.705704507 | 0.538631458 |
| GSM811020 | human PDAC 17 (mRNA) | 0.150173029 | 0.165202352 | 0.281813064 |
| GSM811021 | human PDAC 18 (mRNA) | 0.397029368 | 0.465420328 | 0.557154904 |
| GSM811022 | human PDAC 19 (mRNA) | 0.192571703 | 0.265609035 | 0.238445324 |
| GSM811023 | human PDAC 20 (mRNA) | 0.552251612 | 0.434351862 | 0.394391509 |
| GSM811024 | human PDAC 21 (mRNA) | 0.330888632 | 0.384260378 | 0.333149214 |
| GSM811025 | human PDAC 22 (mRNA) | 0.598564088 | 0.648611329 | 0.603455962 |
| GSM811026 | human PDAC 23 (mRNA) | 0.749295987 | 0.740538234 | 0.697164202 |
| GSM811027 | human PDAC 24 (mRNA) | 1.259531873 | 1.103331354 | 2.358762881 |
| GSM811028 | human PDAC 25 (mRNA) | 0.408774648 | 0.49613895 | 0.399248916 |
| GSM811029 | non-malignant pancreas sample 1 (mRNA) | 0.510905237 | 0.496830666 | 0.289426795 |
| GSM811030 | non-malignant pancreas sample 2 (mRNA) | 0.477498342 | 0.43585378 | 0.186891181 |
| GSM811031 | non-malignant pancreas sample 3 (mRNA) | 0.611052241 | 0.573504268 | 0.330057754 |
| GSM811032 | non-malignant pancreas sample 4 (mRNA) | 1.746961277 | 1.801821541 | 2.122346768 |
| GSM811033 | non-malignant pancreas sample 5 (mRNA) | 1.054403914 | 0.935290299 | 1.283568594 |
| GSM811034 | non-malignant pancreas sample 6 (mRNA) | 0.734460918 | 0.823063776 | 1.456514835 |
| GSM811035 | non-malignant pancreas sample 7 (mRNA) | 1.864718208 | 1.933635853 | 1.331202821 |

| PER2 |  |  |
| --- | --- | --- |
| Sample | Title | FC(205251_at) |
| GSM811004 | human PDAC 1 (mRNA) | 0.839667999 |
| GSM811005 | human PDAC 2 (mRNA) | 0.801786988 |
| GSM811006 | human PDAC 3 (mRNA) | 0.524258739 |
| GSM811007 | human PDAC 4 (mRNA) | 0.658488847 |
| GSM811008 | human PDAC 5 (mRNA) | 0.478168471 |
| GSM811009 | human PDAC 6 (mRNA) | 0.333465846 |
| GSM811010 | human PDAC 7 (mRNA) | 0.449907683 |
| GSM811011 | human PDAC 8 (mRNA) | 0.559636091 |
| GSM811012 | human PDAC 9 (mRNA) | 0.487894427 |
| GSM811013 | human PDAC 10 (mRNA) | 0.555489255 |
| GSM811014 | human PDAC 11 (mRNA) | 0.387039016 |
| GSM811015 | human PDAC 12 (mRNA) | 0.408067837 |
| GSM811016 | human PDAC 13 (mRNA) | 0.21896177 |
| GSM811017 | human PDAC 14 (mRNA) | 1.459486544 |
| GSM811018 | human PDAC 15 (mRNA) | 0.462101254 |
| GSM811019 | human PDAC 16 (mRNA) | 0.577895648 |
| GSM811020 | human PDAC 17 (mRNA) | 0.863052908 |
| GSM811021 | human PDAC 18 (mRNA) | 0.779111065 |
| GSM811022 | human PDAC 19 (mRNA) | 0.694150748 |
| GSM811023 | human PDAC 20 (mRNA) | 0.320167974 |
| GSM811024 | human PDAC 21 (mRNA) | 0.224338776 |
| GSM811025 | human PDAC 22 (mRNA) | 0.565739932 |
| GSM811026 | human PDAC 23 (mRNA) | 0.606887458 |
| GSM811027 | human PDAC 24 (mRNA) | 0.540827685 |
| GSM811028 | human PDAC 25 (mRNA) | 0.442515811 |
| GSM811029 | non-malignant pancreas sample 1 (mRNA) | 1.664479278 |
| GSM811030 | non-malignant pancreas sample 2 (mRNA) | 1.437160222 |
| GSM811031 | non-malignant pancreas sample 3 (mRNA) | 0.489082894 |
| GSM811032 | non-malignant pancreas sample 4 (mRNA) | 0.719796459 |
| GSM811033 | non-malignant pancreas sample 5 (mRNA) | 0.393316035 |
| GSM811034 | non-malignant pancreas sample 6 (mRNA) | 0.60389972 |
| GSM811035 | non-malignant pancreas sample 7 (mRNA) | 1.693327363 |

| CRY1 |  |  |
| --- | --- | --- |
| Sample | Title | FC(209674_at) |
| GSM811004 | human PDAC 1 (mRNA) | 0.505903221 |
| GSM811005 | human PDAC 2 (mRNA) | 0.441306631 |
| GSM811006 | human PDAC 3 (mRNA) | 0.403237951 |
| GSM811007 | human PDAC 4 (mRNA) | 0.287155347 |
| GSM811008 | human PDAC 5 (mRNA) | 0.558501604 |
| GSM811009 | human PDAC 6 (mRNA) | 0.160306333 |
| GSM811010 | human PDAC 7 (mRNA) | 0.678557665 |
| GSM811011 | human PDAC 8 (mRNA) | 0.529643429 |
| GSM811012 | human PDAC 9 (mRNA) | 0.505903221 |
| GSM811013 | human PDAC 10 (mRNA) | 0.69324794 |
| GSM811014 | human PDAC 11 (mRNA) | 0.340062854 |
| GSM811015 | human PDAC 12 (mRNA) | 0.396849345 |
| GSM811016 | human PDAC 13 (mRNA) | 0.451655076 |
| GSM811017 | human PDAC 14 (mRNA) | 0.925203949 |
| GSM811018 | human PDAC 15 (mRNA) | 0.607090427 |
| GSM811019 | human PDAC 16 (mRNA) | 0.571134852 |
| GSM811020 | human PDAC 17 (mRNA) | 0.303231877 |
| GSM811021 | human PDAC 18 (mRNA) | 0.359191055 |
| GSM811022 | human PDAC 19 (mRNA) | 0.617642241 |
| GSM811023 | human PDAC 20 (mRNA) | 0.690657967 |
| GSM811024 | human PDAC 21 (mRNA) | 0.454752454 |
| GSM811025 | human PDAC 22 (mRNA) | 0.576256622 |
| GSM811026 | human PDAC 23 (mRNA) | 0.362967764 |
| GSM811027 | human PDAC 24 (mRNA) | 0.25641869 |
| GSM811028 | human PDAC 25 (mRNA) | 0.550125746 |
| GSM811029 | non-malignant pancreas sample 1 (mRNA) | 1.772079501 |
| GSM811030 | non-malignant pancreas sample 2 (mRNA) | 1.938772238 |
| GSM811031 | non-malignant pancreas sample 3 (mRNA) | 0.461327541 |
| GSM811032 | non-malignant pancreas sample 4 (mRNA) | 0.926230602 |
| GSM811033 | non-malignant pancreas sample 5 (mRNA) | 0.409071045 |
| GSM811034 | non-malignant pancreas sample 6 (mRNA) | 0.521183505 |
| GSM811035 | non-malignant pancreas sample 7 (mRNA) | 0.971336668 |

| CRY2 |  |  |
| --- | --- | --- |
| Sample | Title | FC(212695_at) |
| GSM811004 | human PDAC 1 (mRNA) | 0.629147191 |
| GSM811005 | human PDAC 2 (mRNA) | 0.498231963 |
| GSM811006 | human PDAC 3 (mRNA) | 0.461399864 |
| GSM811007 | human PDAC 4 (mRNA) | 0.646574934 |
| GSM811008 | human PDAC 5 (mRNA) | 0.537150953 |
| GSM811009 | human PDAC 6 (mRNA) | 0.629147191 |
| GSM811010 | human PDAC 7 (mRNA) | 0.665411859 |
| GSM811011 | human PDAC 8 (mRNA) | 0.644909883 |
| GSM811012 | human PDAC 9 (mRNA) | 0.669761561 |
| GSM811013 | human PDAC 10 (mRNA) | 0.629147191 |
| GSM811014 | human PDAC 11 (mRNA) | 0.397192279 |
| GSM811015 | human PDAC 12 (mRNA) | 0.629147191 |
| GSM811016 | human PDAC 13 (mRNA) | 0.629147191 |
| GSM811017 | human PDAC 14 (mRNA) | 1.398547723 |
| GSM811018 | human PDAC 15 (mRNA) | 0.707180051 |
| GSM811019 | human PDAC 16 (mRNA) | 0.633448609 |
| GSM811020 | human PDAC 17 (mRNA) | 0.66107666 |
| GSM811021 | human PDAC 18 (mRNA) | 0.511919719 |
| GSM811022 | human PDAC 19 (mRNA) | 0.488171333 |
| GSM811023 | human PDAC 20 (mRNA) | 0.364388538 |
| GSM811024 | human PDAC 21 (mRNA) | 0.629147191 |
| GSM811025 | human PDAC 22 (mRNA) | 0.629147191 |
| GSM811026 | human PDAC 23 (mRNA) | 0.629147191 |
| GSM811027 | human PDAC 24 (mRNA) | 0.629147191 |
| GSM811028 | human PDAC 25 (mRNA) | 0.629147191 |
| GSM811029 | non-malignant pancreas sample 1 (mRNA) | 2.064281162 |
| GSM811030 | non-malignant pancreas sample 2 (mRNA) | 1.342506882 |
| GSM811031 | non-malignant pancreas sample 3 (mRNA) | 0.629147191 |
| GSM811032 | non-malignant pancreas sample 4 (mRNA) | 0.710063269 |
| GSM811033 | non-malignant pancreas sample 5 (mRNA) | 0.629147191 |
| GSM811034 | non-malignant pancreas sample 6 (mRNA) | 0.801919588 |
| GSM811035 | non-malignant pancreas sample 7 (mRNA) | 0.822934484 |

| DBP |  |  |
| --- | --- | --- |
| Sample | Title | FC(209782_s_at) |
| GSM811004 | human PDAC 1 (mRNA) | 1.50362485 |
| GSM811005 | human PDAC 2 (mRNA) | 0.945306882 |
| GSM811006 | human PDAC 3 (mRNA) | 0.759369871 |
| GSM811007 | human PDAC 4 (mRNA) | 1.690751535 |
| GSM811008 | human PDAC 5 (mRNA) | 0.763470683 |
| GSM811009 | human PDAC 6 (mRNA) | 2.352989143 |
| GSM811010 | human PDAC 7 (mRNA) | 0.62115476 |
| GSM811011 | human PDAC 8 (mRNA) | 0.92211249 |
| GSM811012 | human PDAC 9 (mRNA) | 0.764037134 |
| GSM811013 | human PDAC 10 (mRNA) | 1.32338668 |
| GSM811014 | human PDAC 11 (mRNA) | 2.595090163 |
| GSM811015 | human PDAC 12 (mRNA) | 3.76979132 |
| GSM811016 | human PDAC 13 (mRNA) | 0.818347026 |
| GSM811017 | human PDAC 14 (mRNA) | 0.759369871 |
| GSM811018 | human PDAC 15 (mRNA) | 0.760934756 |
| GSM811019 | human PDAC 16 (mRNA) | 0.760934756 |
| GSM811020 | human PDAC 17 (mRNA) | 2.267424737 |
| GSM811021 | human PDAC 18 (mRNA) | 2.20739477 |
| GSM811022 | human PDAC 19 (mRNA) | 4.838650259 |
| GSM811023 | human PDAC 20 (mRNA) | 0.980809368 |
| GSM811024 | human PDAC 21 (mRNA) | 1.415738356 |
| GSM811025 | human PDAC 22 (mRNA) | 1.600468923 |
| GSM811026 | human PDAC 23 (mRNA) | 3.588409817 |
| GSM811027 | human PDAC 24 (mRNA) | 2.383174619 |
| GSM811028 | human PDAC 25 (mRNA) | 0.92143523 |
| GSM811029 | non-malignant pancreas sample 1 (mRNA) | 0.759369871 |
| GSM811030 | non-malignant pancreas sample 2 (mRNA) | 0.760934756 |
| GSM811031 | non-malignant pancreas sample 3 (mRNA) | 2.367172208 |
| GSM811032 | non-malignant pancreas sample 4 (mRNA) | 0.760934756 |
| GSM811033 | non-malignant pancreas sample 5 (mRNA) | 0.766137192 |
| GSM811034 | non-malignant pancreas sample 6 (mRNA) | 0.872057195 |
| GSM811035 | non-malignant pancreas sample 7 (mRNA) | 0.715813007 |

| RORA |  |  |  |  |  |  |
| --- | --- | --- | --- | --- | --- | --- |
| Sample | Title | FC(226682_at) | FC(235567_at) | FC(210479_s_at) | FC(210426_x_at) | FC(236266_at) |
| GSM811004 | human PDAC 1 (mRNA) | 0.584307541 | 1.290617459 | 1.312412718 | 1.843813621 | 0.894241098 |
| GSM811005 | human PDAC 2 (mRNA) | 0.141491747 | 0.212319892 | 0.162539411 | 0.195650789 | 0.361767762 |
| GSM811006 | human PDAC 3 (mRNA) | 0.325978747 | 0.356277047 | 0.535430594 | 0.626025284 | 0.894241098 |
| GSM811007 | human PDAC 4 (mRNA) | 0.31606207 | 0.197215035 | 0.252041198 | 0.309708489 | 0.27896688 |
| GSM811008 | human PDAC 5 (mRNA) | 0.804324635 | 0.858393732 | 0.959484549 | 0.94438225 | 0.880878995 |
| GSM811009 | human PDAC 6 (mRNA) | 0.587467039 | 0.837424741 | 0.713597012 | 1.11540901 | 1.249079893 |
| GSM811010 | human PDAC 7 (mRNA) | 1.058300853 | 0.759773578 | 1.102020819 | 1.339368395 | 0.945065192 |
| GSM811011 | human PDAC 8 (mRNA) | 0.420410269 | 0.175420766 | 0.288697692 | 0.47604598 | 0.259399168 |
| GSM811012 | human PDAC 9 (mRNA) | 0.835325169 | 0.812320775 | 1.720396951 | 2.251322647 | 1.762344243 |
| GSM811013 | human PDAC 10 (mRNA) | 0.442006599 | 0.290907309 | 0.333974875 | 0.535089762 | 0.345312809 |
| GSM811014 | human PDAC 11 (mRNA) | 0.81845773 | 0.768896569 | 0.848338228 | 0.807521642 | 0.823377401 |
| GSM811015 | human PDAC 12 (mRNA) | 0.784176228 | 1.464655579 | 1.541635266 | 1.820434497 | 1.933461124 |
| GSM811016 | human PDAC 13 (mRNA) | 0.932302353 | 1.217676959 | 1.199922709 | 1.025610424 | 1.355331494 |
| GSM811017 | human PDAC 14 (mRNA) | 1.405058125 | 0.912446229 | 1.54087676 | 1.671395122 | 1.185721964 |
| GSM811018 | human PDAC 15 (mRNA) | 1.071149472 | 0.702004142 | 1.085531416 | 1.104790509 | 0.988118352 |
| GSM811019 | human PDAC 16 (mRNA) | 1.425363428 | 1.099670608 | 1.458152367 | 1.444262349 | 1.368585679 |
| GSM811020 | human PDAC 17 (mRNA) | 0.057777829 | 0.151318622 | 0.10294042 | 0.107341721 | 0.246507895 |
| GSM811021 | human PDAC 18 (mRNA) | 0.651849047 | 0.762184116 | 0.785945663 | 0.851030552 | 1.011913349 |
| GSM811022 | human PDAC 19 (mRNA) | 0.465302168 | 0.427128316 | 0.464716083 | 0.562896987 | 0.488085486 |
| GSM811023 | human PDAC 20 (mRNA) | 0.972277347 | 0.690355534 | 0.883897803 | 0.828637393 | 0.640675513 |
| GSM811024 | human PDAC 21 (mRNA) | 0.36835783 | 0.3738811 | 0.589311155 | 0.644254597 | 0.868787804 |
| GSM811025 | human PDAC 22 (mRNA) | 0.518235522 | 0.978233955 | 0.985413731 | 1.011203127 | 2.73065255 |
| GSM811026 | human PDAC 23 (mRNA) | 0.543800662 | 0.825489316 | 0.697585269 | 0.838843063 | 1.030679482 |
| GSM811027 | human PDAC 24 (mRNA) | 1.03444706 | 1.278694344 | 1.136636918 | 1.176085406 | 1.294141848 |
| GSM811028 | human PDAC 25 (mRNA) | 0.405094335 | 0.617388899 | 1.185989699 | 1.376221636 | 1.013169643 |
| GSM811029 | non-malignant pancreas sample 1 (mRNA) | 0.508299182 | 0.34830219 | 0.628215515 | 0.744174484 | 0.976015359 |
| GSM811030 | non-malignant pancreas sample 2 (mRNA) | 1.290947037 | 1.247155578 | 0.996126921 | 1.00424608 | 0.996689308 |
| GSM811031 | non-malignant pancreas sample 3 (mRNA) | 1.463405731 | 1.278304421 | 1.428450932 | 1.134426257 | 0.9857309 |
| GSM811032 | non-malignant pancreas sample 4 (mRNA) | 0.897715253 | 1.301713043 | 0.964592488 | 0.901920908 | 1.641341861 |
| GSM811033 | non-malignant pancreas sample 5 (mRNA) | 0.999785018 | 1.181018405 | 1.200222166 | 1.18239562 | 0.894241098 |
| GSM811034 | non-malignant pancreas sample 6 (mRNA) | 1.088272474 | 1.266723324 | 0.936526342 | 0.921734292 | 1.020527993 |
| GSM811035 | non-malignant pancreas sample 7 (mRNA) | 0.751575432 | 0.37678448 | 0.849238381 | 1.111103207 | 0.485453749 |

**Supplementary Table S3. Expression of the clock genes in the GSE16515 dataset**

| BMAL1 |  |  |  |
| --- | --- | --- | --- |
| Sample | Title | FC(209824_s_at) | FC(210971_s_at) |
| GSM414924 | Pancreatic Sample 6-Tumor | 0.491930209 | 0.370974515 |
| GSM414925 | Pancreatic Sample 7-Tumor | 1.047938165 | 0.663439648 |
| GSM414926 | Pancreatic Sample 1-Tumor | 0.567188188 | 0.500762549 |
| GSM414927 | Pancreatic Sample 16-Tumor | 1.269874114 | 1.228919391 |
| GSM414929 | Pancreatic Sample 53-Tumor | 1.019570412 | 1.085515991 |
| GSM414931 | Pancreatic Sample 9-Tumor | 0.816323806 | 0.835842009 |
| GSM414933 | Pancreatic Sample 11-Tumor | 0.35568156 | 0.322701854 |
| GSM414935 | Pancreatic Sample 12-Tumor | 0.586878746 | 0.506951352 |
| GSM414936 | Pancreatic Sample 13-Tumor | 0.876062956 | 0.787922994 |
| GSM414937 | Pancreatic Sample 54-Tumor | 1.11051409 | 1.290807418 |
| GSM414939 | Pancreatic Sample 15-Tumor | 0.734224192 | 0.672634441 |
| GSM414941 | Pancreatic Sample 14-Tumor | 0.976184437 | 0.821342528 |
| GSM414943 | Pancreatic Sample 17-Tumor | 0.911105475 | 0.630373759 |
| GSM414944 | Pancreatic Sample 20-Tumor | 0.526388685 | 0.816921955 |
| GSM414945 | Pancreatic Sample 5-Tumor | 1.251518509 | 1.315562628 |
| GSM414946 | Pancreatic Sample 4-Tumor | 0.94865103 | 0.716486528 |
| GSM414948 | Pancreatic Sample 19-Tumor | 0.41942557 | 0.429679729 |
| GSM414949 | Pancreatic Sample 21-Tumor | 1.225653793 | 0.760515439 |
| GSM414950 | Pancreatic Sample 22-Tumor | 1.409209841 | 1.104612868 |
| GSM414951 | Pancreatic Sample 23-Tumor | 0.825334739 | 0.895961806 |
| GSM414952 | Pancreatic Sample 24-Tumor | 1.01039261 | 1.264814446 |
| GSM414954 | Pancreatic Sample 25-Tumor | 1.182267818 | 1.28674049 |
| GSM414956 | Pancreatic Sample 30-Tumor | 1.17225567 | 1.173927458 |
| GSM414958 | Pancreatic Sample 26-Tumor | 0.701184103 | 0.940874831 |
| GSM414959 | Pancreatic Sample 28-Tumor | 0.572361132 | 0.463983379 |
| GSM414960 | Pancreatic Sample 32-Tumor | 0.522634129 | 0.40740004 |
| GSM414961 | Pancreatic Sample 29-Tumor | 0.878565993 | 1.113277192 |
| GSM414962 | Pancreatic Sample 31-Tumor | 0.708526345 | 0.494396923 |
| GSM414964 | Pancreatic Sample -Tumor | 0.587462788 | 0.933094622 |
| GSM414965 | Pancreatic Sample 33-Tumor | 1.033754289 | 1.047852706 |
| GSM414967 | Pancreatic Sample 27-Tumor | 0.360771069 | 0.451252127 |
| GSM414968 | Pancreatic Sample 34-Tumor | 0.749743022 | 0.608270893 |
| GSM414969 | Pancreatic Sample 35-Tumor | 0.583624798 | 0.741772208 |
| GSM414971 | Pancreatic Sample 36-Tumor | 0.677238383 | 0.898437327 |
| GSM414973 | Pancreatic Sample 37-Tumor | 0.671064225 | 1.205401941 |
| GSM414974 | Pancreatic Sample 38-Tumor | 0.439867039 | 0.322701854 |
| GSM414928 | Pancreatic Sample 16-Normal | 1.255690237 | 1.152885529 |
| GSM414930 | Pancreatic Sample 53-Normal | 0.602314141 | 0.322701854 |
| GSM414932 | Pancreatic Sample 9-Normal | 1.119691893 | 1.220078244 |
| GSM414934 | Pancreatic Sample 11-Normal | 1.335787422 | 1.221846473 |
| GSM414938 | Pancreatic Sample 54-Normal | 1.706236901 | 2.11480229 |
| GSM414940 | Pancreatic Sample 15-Normal | 1.034588634 | 0.759808147 |
| GSM414942 | Pancreatic Sample 14-Normal | 0.771769747 | 0.941582123 |
| GSM414947 | Pancreatic Sample 4-Normal | 1.048772511 | 0.629843291 |
| GSM414953 | Pancreatic Sample 24-Normal | 1.415050261 | 1.419357691 |
| GSM414955 | Pancreatic Sample 25-Normal | 1.195617349 | 2.047609575 |
| GSM414957 | Pancreatic Sample 30-Normal | 0.668561188 | 0.92389983 |
| GSM414963 | Pancreatic Sample 31-Normal | 1.134710115 | 0.853701125 |
| GSM414966 | Pancreatic Sample 33-Normal | 0.931964116 | 0.948124572 |
| GSM414970 | Pancreatic Sample 35-Normal | 1.095495868 | 1.145458966 |
| GSM414972 | Pancreatic Sample 36-Normal | 0.396481064 | 0.184779966 |
| GSM414975 | Pancreatic Sample 38-Normal | 0.287265215 | 0.113520324 |

| CLOCK |  |  |  |  |  |
| --- | --- | --- | --- | --- | --- |
| Sample | Title | FC(204980_at) | FC(217563_at) | FC(225856_at) | FC(227531_at) |
| GSM414924 | Pancreatic Sample 6-Tumor | 1.558458108 | 0.761253701 | 1.415212528 | 1.024382763 |
| GSM414925 | Pancreatic Sample 7-Tumor | 2.494424976 | 0.771880407 | 1.785682327 | 1.59362061 |
| GSM414926 | Pancreatic Sample 1-Tumor | 1.413188914 | 0.77724926 | 1.04787472 | 1.060941573 |
| GSM414927 | Pancreatic Sample 16-Tumor | 1.204205161 | 0.92431035 | 1.148545861 | 1.042294127 |
| GSM414929 | Pancreatic Sample 53-Tumor | 1.302325581 | 0.815891515 | 1.139597315 | 1.055052906 |
| GSM414931 | Pancreatic Sample 9-Tumor | 1.020070086 | 0.771880407 | 0.729306488 | 0.812390738 |
| GSM414933 | Pancreatic Sample 11-Tumor | 1.626632686 | 0.788099255 | 0.834451902 | 0.682840055 |
| GSM414935 | Pancreatic Sample 12-Tumor | 1.376871615 | 0.720189885 | 1.296196868 | 1.244471707 |
| GSM414936 | Pancreatic Sample 13-Tumor | 0.827652119 | 0.750773296 | 0.719463087 | 0.607023463 |
| GSM414937 | Pancreatic Sample 54-Tumor | 1.319528512 | 0.766548639 | 1.223713647 | 1.200552063 |
| GSM414939 | Pancreatic Sample 15-Tumor | 1.049378783 | 0.761253701 | 0.77360179 | 0.721116393 |
| GSM414941 | Pancreatic Sample 14-Tumor | 1.987894234 | 0.750773296 | 1.674720358 | 1.604907223 |
| GSM414943 | Pancreatic Sample 17-Tumor | 1.524689392 | 0.844663867 | 1.126621924 | 1.127434443 |
| GSM414944 | Pancreatic Sample 20-Tumor | 0.784326219 | 0.862412051 | 0.794630872 | 0.793743291 |
| GSM414945 | Pancreatic Sample 5-Tumor | 0.906658171 | 0.793580919 | 0.817449664 | 0.656095691 |
| GSM414946 | Pancreatic Sample 4-Tumor | 1.747690347 | 0.705368592 | 1.36196868 | 0.975801257 |
| GSM414948 | Pancreatic Sample 19-Tumor | 1.341191462 | 0.815891515 | 1.067114094 | 1.074927158 |
| GSM414949 | Pancreatic Sample 21-Tumor | 1.134756292 | 1.362682296 | 1.153020134 | 1.205950008 |
| GSM414950 | Pancreatic Sample 22-Tumor | 1.136667729 | 0.745587327 | 1.096196868 | 0.950038338 |
| GSM414951 | Pancreatic Sample 23-Tumor | 1.303599873 | 0.700496255 | 1.041610738 | 0.9591167 |
| GSM414952 | Pancreatic Sample 24-Tumor | 0.776680471 | 0.690852316 | 1.053691275 | 0.856310382 |
| GSM414954 | Pancreatic Sample 25-Tumor | 1.287034087 | 0.844663867 | 1.630425056 | 1.230976844 |
| GSM414956 | Pancreatic Sample 30-Tumor | 1.146224912 | 0.804658896 | 1.004026846 | 0.987823953 |
| GSM414958 | Pancreatic Sample 26-Tumor | 1.260273973 | 0.782655456 | 1.124384787 | 1.065358074 |
| GSM414959 | Pancreatic Sample 28-Tumor | 0.812360624 | 0.750773296 | 0.598210291 | 0.618064714 |
| GSM414960 | Pancreatic Sample 32-Tumor | 1.016884358 | 0.911585096 | 0.609395973 | 0.436742831 |
| GSM414961 | Pancreatic Sample 29-Tumor | 1.37623447 | 0.983808376 | 1.465771812 | 1.484189541 |
| GSM414962 | Pancreatic Sample 31-Tumor | 1.11627907 | 0.963561892 | 1.453691275 | 0.799141236 |
| GSM414964 | Pancreatic Sample -Tumor | 1.431666136 | 0.740437179 | 1.300671141 | 1.142401472 |
| GSM414965 | Pancreatic Sample 33-Tumor | 1.841987894 | 0.956906084 | 1.383892617 | 1.151725196 |
| GSM414967 | Pancreatic Sample 27-Tumor | 0.435998726 | 0.821566489 | 0.402774049 | 0.303021009 |
| GSM414968 | Pancreatic Sample 34-Tumor | 1.02389296 | 0.750773296 | 1.179418345 | 0.927219752 |
| GSM414969 | Pancreatic Sample 35-Tumor | 1.13794202 | 0.771880407 | 0.820134228 | 1.644165005 |
| GSM414971 | Pancreatic Sample 36-Tumor | 1.216310927 | 0.930739437 | 0.878299776 | 1.034933292 |
| GSM414973 | Pancreatic Sample 37-Tumor | 1.118190507 | 0.730243361 | 0.760178971 | 1.277104739 |
| GSM414974 | Pancreatic Sample 38-Tumor | 0.911755336 | 0.821566489 | 0.763310962 | 1.21944487 |
| GSM414928 | Pancreatic Sample 16-Normal | 1.039821599 | 0.827280936 | 1.032662192 | 1.183376783 |
| GSM414930 | Pancreatic Sample 53-Normal | 1.207390889 | 0.943732075 | 0.732885906 | 0.67106272 |
| GSM414932 | Pancreatic Sample 9-Normal | 1.161516406 | 0.788099255 | 1.102908277 | 1.097009661 |
| GSM414934 | Pancreatic Sample 11-Normal | 1.220770946 | 0.850538969 | 1.030425056 | 0.877902162 |
| GSM414938 | Pancreatic Sample 54-Normal | 1.427206117 | 0.761253701 | 1.412080537 | 1.250115013 |
| GSM414940 | Pancreatic Sample 15-Normal | 1.225868111 | 0.750773296 | 1.054138702 | 0.743198896 |
| GSM414942 | Pancreatic Sample 14-Normal | 0.895826696 | 0.990651304 | 0.853243848 | 0.715963809 |
| GSM414947 | Pancreatic Sample 4-Normal | 1.216948073 | 1.011466994 | 0.944519016 | 0.778776261 |
| GSM414953 | Pancreatic Sample 24-Normal | 1.075501752 | 0.710274818 | 1.153467562 | 1.045729183 |
| GSM414955 | Pancreatic Sample 25-Normal | 1.144950621 | 0.740437179 | 1.431319911 | 1.222879926 |
| GSM414957 | Pancreatic Sample 30-Normal | 0.572793883 | 0.997541829 | 1.079642058 | 0.827603128 |
| GSM414963 | Pancreatic Sample 31-Normal | 1.325899968 | 0.804658896 | 1.434451902 | 1.094065327 |
| GSM414966 | Pancreatic Sample 33-Normal | 0.647977063 | 2.008960562 | 0.708724832 | 1.199325257 |
| GSM414970 | Pancreatic Sample 35-Normal | 0.937878305 | 0.862412051 | 0.558836689 | 1.202760313 |
| GSM414972 | Pancreatic Sample 36-Normal | 0.364765849 | 1.153844846 | 0.812527964 | 0.817543322 |
| GSM414975 | Pancreatic Sample 38-Normal | 0.534947435 | 1.798070069 | 0.658612975 | 1.272688238 |

| PER1 |  |  |  |  |  |
| --- | --- | --- | --- | --- | --- |
| Sample | Title | FC(242832_at) | FC(244677_at) | FC(36829_at) | FC(202861_at) |
| GSM414924 | Pancreatic Sample 6-Tumor | 0.902774037 | 0.54852293 | 0.582431939 | 0.545642568 |
| GSM414925 | Pancreatic Sample 7-Tumor | 0.876918933 | 0.635450116 | 0.717957568 | 0.696436965 |
| GSM414926 | Pancreatic Sample 1-Tumor | 0.932938325 | 0.446877143 | 0.562736173 | 0.652439598 |
| GSM414927 | Pancreatic Sample 16-Tumor | 0.902774037 | 0.572276713 | 0.743280696 | 0.808498637 |
| GSM414929 | Pancreatic Sample 53-Tumor | 0.902774037 | 0.259251406 | 0.504586769 | 0.439221585 |
| GSM414931 | Pancreatic Sample 9-Tumor | 1.303528144 | 1.117010696 | 0.716550727 | 0.640782175 |
| GSM414933 | Pancreatic Sample 11-Tumor | 2.01885268 | 0.540872172 | 0.535068311 | 0.462912475 |
| GSM414935 | Pancreatic Sample 12-Tumor | 0.907083221 | 0.780377335 | 0.966030431 | 1.089028862 |
| GSM414936 | Pancreatic Sample 13-Tumor | 0.883382709 | 0.393467564 | 0.436448797 | 0.438093447 |
| GSM414937 | Pancreatic Sample 54-Tumor | 0.909237813 | 0.186897093 | 0.550543556 | 0.602425496 |
| GSM414939 | Pancreatic Sample 15-Tumor | 0.935092917 | 0.551000318 | 0.799085366 | 0.731409232 |
| GSM414941 | Pancreatic Sample 14-Tumor | 0.838136278 | 0.616869703 | 0.838945845 | 0.864529473 |
| GSM414943 | Pancreatic Sample 17-Tumor | 1.441422031 | 0.896960317 | 0.752190685 | 0.817147692 |
| GSM414944 | Pancreatic Sample 20-Tumor | 1.083759763 | 0.423269089 | 0.531785684 | 0.597160854 |
| GSM414945 | Pancreatic Sample 5-Tumor | 0.920010773 | 0.166786529 | 0.359353941 | 0.270565009 |
| GSM414946 | Pancreatic Sample 4-Tumor | 0.883382709 | 0.387565551 | 0.677628142 | 0.55918022 |
| GSM414948 | Pancreatic Sample 19-Tumor | 0.900619445 | 0.45518368 | 0.472698386 | 0.494876375 |
| GSM414949 | Pancreatic Sample 21-Tumor | 0.863991382 | 0.51245507 | 0.805650621 | 0.810378866 |
| GSM414950 | Pancreatic Sample 22-Tumor | 0.825208726 | 0.544296797 | 0.618540844 | 0.66409702 |
| GSM414951 | Pancreatic Sample 23-Tumor | 0.868300566 | 0.29772379 | 0.484422056 | 0.468929209 |
| GSM414952 | Pancreatic Sample 24-Tumor | 1.105305683 | 0.809523081 | 0.943052037 | 1.448152675 |
| GSM414954 | Pancreatic Sample 25-Tumor | 1.038513332 | 0.378020319 | 0.707171791 | 0.889724546 |
| GSM414956 | Pancreatic Sample 30-Tumor | 0.848909238 | 0.912990477 | 0.718895461 | 0.762244994 |
| GSM414958 | Pancreatic Sample 26-Tumor | 0.904928629 | 0.208756402 | 0.446812522 | 0.52909655 |
| GSM414959 | Pancreatic Sample 28-Tumor | 1.068677619 | 1.275855009 | 1.197690156 | 1.33458682 |
| GSM414960 | Pancreatic Sample 32-Tumor | 0.952329653 | 0.212763942 | 0.379002813 | 0.299370123 |
| GSM414961 | Pancreatic Sample 29-Tumor | 0.900619445 | 0.424362054 | 0.801899047 | 0.986368337 |
| GSM414962 | Pancreatic Sample 31-Tumor | 0.973875572 | 0.873643721 | 0.664028685 | 0.854376234 |
| GSM414964 | Pancreatic Sample -Tumor | 0.913546997 | 0.315356966 | 0.770479611 | 0.771646141 |
| GSM414965 | Pancreatic Sample 33-Tumor | 1.118233235 | 0.73520143 | 0.421114236 | 0.561812541 |
| GSM414967 | Pancreatic Sample 27-Tumor | 1.133315378 | 0.509176174 | 0.800492207 | 0.985992291 |
| GSM414968 | Pancreatic Sample 34-Tumor | 0.907083221 | 0.286648407 | 0.730150185 | 0.677258626 |
| GSM414969 | Pancreatic Sample 35-Tumor | 0.920010773 | 0.413359535 | 0.516779386 | 0.518191219 |
| GSM414971 | Pancreatic Sample 36-Tumor | 0.935092917 | 0.2668293 | 0.283056295 | 0.278048322 |
| GSM414973 | Pancreatic Sample 37-Tumor | 0.87260975 | 0.403158524 | 0.818312185 | 0.803233995 |
| GSM414974 | Pancreatic Sample 38-Tumor | 0.950175061 | 1.102437823 | 1.035903506 | 0.950643979 |
| GSM414928 | Pancreatic Sample 16-Normal | 0.855373014 | 0.430409796 | 0.739529121 | 0.673122121 |
| GSM414930 | Pancreatic Sample 53-Normal | 1.163479666 | 1.731985926 | 1.131568655 | 1.027733383 |
| GSM414932 | Pancreatic Sample 9-Normal | 0.922165365 | 0.375397202 | 0.716081781 | 0.765253361 |
| GSM414934 | Pancreatic Sample 11-Normal | 0.926474549 | 0.310329325 | 0.550074609 | 0.505405659 |
| GSM414938 | Pancreatic Sample 54-Normal | 0.917856181 | 0.208537809 | 0.638705557 | 0.659208423 |
| GSM414940 | Pancreatic Sample 15-Normal | 0.920010773 | 0.561274194 | 0.88818526 | 0.808874683 |
| GSM414942 | Pancreatic Sample 14-Normal | 1.029894964 | 2.211433439 | 1.857029372 | 1.829087149 |
| GSM414947 | Pancreatic Sample 4-Normal | 1.006194452 | 1.068920216 | 1.071074517 | 0.980727649 |
| GSM414953 | Pancreatic Sample 24-Normal | 0.922165365 | 0.540872172 | 0.953368867 | 0.969070227 |
| GSM414955 | Pancreatic Sample 25-Normal | 0.997576084 | 0.635814438 | 0.928514686 | 0.969822318 |
| GSM414957 | Pancreatic Sample 30-Normal | 1.001885268 | 1.346533441 | 1.041999814 | 1.353389113 |
| GSM414963 | Pancreatic Sample 31-Normal | 0.935092917 | 0.678585819 | 1.070136623 | 1.230046066 |
| GSM414966 | Pancreatic Sample 33-Normal | 1.00403986 | 1.973166969 | 1.270845858 | 1.492526088 |
| GSM414970 | Pancreatic Sample 35-Normal | 1.038513332 | 0.388439923 | 0.7793896 | 0.513678669 |
| GSM414972 | Pancreatic Sample 36-Normal | 0.943711285 | 1.207362507 | 0.674345514 | 0.789696343 |
| GSM414975 | Pancreatic Sample 38-Normal | 1.415566927 | 2.330930995 | 1.689146414 | 1.432358748 |

| PER2 |  |  |  |
| --- | --- | --- | --- |
| Sample | Title | FC(208518_s_at) | FC(205251_at) |
| GSM414924 | Pancreatic Sample 6-Tumor | 0.930805557 | 0.601694738 |
| GSM414925 | Pancreatic Sample 7-Tumor | 0.888098008 | 1.135917674 |
| GSM414926 | Pancreatic Sample 1-Tumor | 1.049072617 | 0.966811133 |
| GSM414927 | Pancreatic Sample 16-Tumor | 1.052357813 | 0.653793218 |
| GSM414929 | Pancreatic Sample 53-Tumor | 1.656833892 | 1.903729696 |
| GSM414931 | Pancreatic Sample 9-Tumor | 0.999794675 | 0.487248897 |
| GSM414933 | Pancreatic Sample 11-Tumor | 0.948326603 | 0.845959742 |
| GSM414935 | Pancreatic Sample 12-Tumor | 0.867291766 | 0.845105668 |
| GSM414936 | Pancreatic Sample 13-Tumor | 1.046882486 | 0.880549716 |
| GSM414937 | Pancreatic Sample 54-Tumor | 0.922045035 | 0.85919788 |
| GSM414939 | Pancreatic Sample 15-Tumor | 0.915474642 | 0.866884541 |
| GSM414941 | Pancreatic Sample 14-Tumor | 1.911984122 | 1.202962439 |
| GSM414943 | Pancreatic Sample 17-Tumor | 0.673465197 | 0.899766368 |
| GSM414944 | Pancreatic Sample 20-Tumor | 0.893573335 | 0.330142088 |
| GSM414945 | Pancreatic Sample 5-Tumor | 0.90890425 | 1.128231013 |
| GSM414946 | Pancreatic Sample 4-Tumor | 0.914379577 | 0.77592572 |
| GSM414948 | Pancreatic Sample 19-Tumor | 1.306412977 | 0.917274874 |
| GSM414949 | Pancreatic Sample 21-Tumor | 0.934090754 | 1.035564045 |
| GSM414950 | Pancreatic Sample 22-Tumor | 0.879337485 | 0.651658034 |
| GSM414951 | Pancreatic Sample 23-Tumor | 0.890288139 | 1.077413644 |
| GSM414952 | Pancreatic Sample 24-Tumor | 1.534186572 | 1.15470729 |
| GSM414954 | Pancreatic Sample 25-Tumor | 0.922045035 | 0.394624633 |
| GSM414956 | Pancreatic Sample 30-Tumor | 1.219902813 | 0.845532705 |
| GSM414958 | Pancreatic Sample 26-Tumor | 1.054547943 | 0.946313371 |
| GSM414959 | Pancreatic Sample 28-Tumor | 1.976592978 | 1.876399346 |
| GSM414960 | Pancreatic Sample 32-Tumor | 0.942851276 | 0.480843346 |
| GSM414961 | Pancreatic Sample 29-Tumor | 1.049072617 | 0.75628203 |
| GSM414962 | Pancreatic Sample 31-Tumor | 0.920949969 | 0.881403789 |
| GSM414964 | Pancreatic Sample -Tumor | 0.917664773 | 1.081684011 |
| GSM414965 | Pancreatic Sample 33-Tumor | 0.905619054 | 0.45436707 |
| GSM414967 | Pancreatic Sample 27-Tumor | 1.004174937 | 0.334754084 |
| GSM414968 | Pancreatic Sample 34-Tumor | 0.929710492 | 1.309294582 |
| GSM414969 | Pancreatic Sample 35-Tumor | 1.429060297 | 1.243530927 |
| GSM414971 | Pancreatic Sample 36-Tumor | 0.909999316 | 0.561980323 |
| GSM414973 | Pancreatic Sample 37-Tumor | 0.922045035 | 0.837419007 |
| GSM414974 | Pancreatic Sample 38-Tumor | 0.960372322 | 1.443811149 |
| GSM414928 | Pancreatic Sample 16-Normal | 0.951611799 | 0.642690263 |
| GSM414930 | Pancreatic Sample 53-Normal | 1.276846212 | 1.085527341 |
| GSM414932 | Pancreatic Sample 9-Normal | 0.924235165 | 0.728097607 |
| GSM414934 | Pancreatic Sample 11-Normal | 0.93956608 | 1.520677759 |
| GSM414938 | Pancreatic Sample 54-Normal | 0.932995688 | 1.192713558 |
| GSM414940 | Pancreatic Sample 15-Normal | 1.026076244 | 1.668005427 |
| GSM414942 | Pancreatic Sample 14-Normal | 1.04359729 | 0.914285617 |
| GSM414947 | Pancreatic Sample 4-Normal | 0.942851276 | 0.866884541 |
| GSM414953 | Pancreatic Sample 24-Normal | 0.913284512 | 1.752985734 |
| GSM414955 | Pancreatic Sample 25-Normal | 0.884812812 | 0.655928401 |
| GSM414957 | Pancreatic Sample 30-Normal | 0.952706865 | 0.777633866 |
| GSM414963 | Pancreatic Sample 31-Normal | 0.949421669 | 0.840408264 |
| GSM414966 | Pancreatic Sample 33-Normal | 0.734788858 | 0.560699213 |
| GSM414970 | Pancreatic Sample 35-Normal | 0.913284512 | 0.96595706 |
| GSM414972 | Pancreatic Sample 36-Normal | 1.005270002 | 0.38796286 |
| GSM414975 | Pancreatic Sample 38-Normal | 1.608651016 | 1.439540782 |

| CRY1 |  |  |
| --- | --- | --- |
| Sample | Title | FC(209674_at) |
| GSM414924 | Pancreatic Sample 6-Tumor | 0.852890462 |
| GSM414925 | Pancreatic Sample 7-Tumor | 0.922588257 |
| GSM414926 | Pancreatic Sample 1-Tumor | 0.520918414 |
| GSM414927 | Pancreatic Sample 16-Tumor | 0.70950177 |
| GSM414929 | Pancreatic Sample 53-Tumor | 1.211362193 |
| GSM414931 | Pancreatic Sample 9-Tumor | 0.751973863 |
| GSM414933 | Pancreatic Sample 11-Tumor | 1.117524276 |
| GSM414935 | Pancreatic Sample 12-Tumor | 1.104818949 |
| GSM414936 | Pancreatic Sample 13-Tumor | 1.175061258 |
| GSM414937 | Pancreatic Sample 54-Tumor | 1.119339323 |
| GSM414939 | Pancreatic Sample 15-Tumor | 0.902622743 |
| GSM414941 | Pancreatic Sample 14-Tumor | 1.218985389 |
| GSM414943 | Pancreatic Sample 17-Tumor | 1.251474725 |
| GSM414944 | Pancreatic Sample 20-Tumor | 0.830020873 |
| GSM414945 | Pancreatic Sample 5-Tumor | 1.146202015 |
| GSM414946 | Pancreatic Sample 4-Tumor | 1.432979399 |
| GSM414948 | Pancreatic Sample 19-Tumor | 0.776113985 |
| GSM414949 | Pancreatic Sample 21-Tumor | 0.886105817 |
| GSM414950 | Pancreatic Sample 22-Tumor | 0.982303294 |
| GSM414951 | Pancreatic Sample 23-Tumor | 0.664307106 |
| GSM414952 | Pancreatic Sample 24-Tumor | 0.840185135 |
| GSM414954 | Pancreatic Sample 25-Tumor | 0.628913695 |
| GSM414956 | Pancreatic Sample 30-Tumor | 0.797350032 |
| GSM414958 | Pancreatic Sample 26-Tumor | 0.988655958 |
| GSM414959 | Pancreatic Sample 28-Tumor | 0.90407478 |
| GSM414960 | Pancreatic Sample 32-Tumor | 0.90407478 |
| GSM414961 | Pancreatic Sample 29-Tumor | 0.821671658 |
| GSM414962 | Pancreatic Sample 31-Tumor | 0.957074145 |
| GSM414964 | Pancreatic Sample -Tumor | 0.732552863 |
| GSM414965 | Pancreatic Sample 33-Tumor | 0.663218078 |
| GSM414967 | Pancreatic Sample 27-Tumor | 0.748706779 |
| GSM414968 | Pancreatic Sample 34-Tumor | 0.970686995 |
| GSM414969 | Pancreatic Sample 35-Tumor | 0.828205826 |
| GSM414971 | Pancreatic Sample 36-Tumor | 1.024412379 |
| GSM414973 | Pancreatic Sample 37-Tumor | 0.727289228 |
| GSM414974 | Pancreatic Sample 38-Tumor | 0.646701153 |
| GSM414928 | Pancreatic Sample 16-Normal | 0.862873219 |
| GSM414930 | Pancreatic Sample 53-Normal | 1.205009529 |
| GSM414932 | Pancreatic Sample 9-Normal | 1.3825211 |
| GSM414934 | Pancreatic Sample 11-Normal | 1.876758327 |
| GSM414938 | Pancreatic Sample 54-Normal | 1.313912333 |
| GSM414940 | Pancreatic Sample 15-Normal | 1.233687267 |
| GSM414942 | Pancreatic Sample 14-Normal | 0.950176967 |
| GSM414947 | Pancreatic Sample 4-Normal | 0.91768763 |
| GSM414953 | Pancreatic Sample 24-Normal | 1.542971232 |
| GSM414955 | Pancreatic Sample 25-Normal | 0.817678555 |
| GSM414957 | Pancreatic Sample 30-Normal | 0.628187676 |
| GSM414963 | Pancreatic Sample 31-Normal | 1.089028042 |
| GSM414966 | Pancreatic Sample 33-Normal | 0.455032217 |
| GSM414970 | Pancreatic Sample 35-Normal | 1.191941192 |
| GSM414972 | Pancreatic Sample 36-Normal | 0.274979581 |
| GSM414975 | Pancreatic Sample 38-Normal | 0.257555132 |

| CRY2 |  |  |
| --- | --- | --- |
| Sample | Title | FC(212695_at) |
| GSM414924 | Pancreatic Sample 6-Tumor | 0.996592998 |
| GSM414925 | Pancreatic Sample 7-Tumor | 0.882899371 |
| GSM414926 | Pancreatic Sample 1-Tumor | 0.886879513 |
| GSM414927 | Pancreatic Sample 16-Tumor | 0.90868377 |
| GSM414929 | Pancreatic Sample 53-Tumor | 0.531781609 |
| GSM414931 | Pancreatic Sample 9-Tumor | 1.172757553 |
| GSM414933 | Pancreatic Sample 11-Tumor | 0.888783059 |
| GSM414935 | Pancreatic Sample 12-Tumor | 1.168258262 |
| GSM414936 | Pancreatic Sample 13-Tumor | 0.757092267 |
| GSM414937 | Pancreatic Sample 54-Tumor | 0.863517809 |
| GSM414939 | Pancreatic Sample 15-Tumor | 0.8680171 |
| GSM414941 | Pancreatic Sample 14-Tumor | 0.860749014 |
| GSM414943 | Pancreatic Sample 17-Tumor | 0.79152915 |
| GSM414944 | Pancreatic Sample 20-Tumor | 0.793778795 |
| GSM414945 | Pancreatic Sample 5-Tumor | 0.822505039 |
| GSM414946 | Pancreatic Sample 4-Tumor | 1.032760377 |
| GSM414948 | Pancreatic Sample 19-Tumor | 0.887398662 |
| GSM414949 | Pancreatic Sample 21-Tumor | 0.802604328 |
| GSM414950 | Pancreatic Sample 22-Tumor | 0.83738731 |
| GSM414951 | Pancreatic Sample 23-Tumor | 0.728019924 |
| GSM414952 | Pancreatic Sample 24-Tumor | 1.311370332 |
| GSM414954 | Pancreatic Sample 25-Tumor | 0.780627021 |
| GSM414956 | Pancreatic Sample 30-Tumor | 0.870958944 |
| GSM414958 | Pancreatic Sample 26-Tumor | 0.790490852 |
| GSM414959 | Pancreatic Sample 28-Tumor | 1.022031298 |
| GSM414960 | Pancreatic Sample 32-Tumor | 0.932910723 |
| GSM414961 | Pancreatic Sample 29-Tumor | 0.983960373 |
| GSM414962 | Pancreatic Sample 31-Tumor | 1.018916405 |
| GSM414964 | Pancreatic Sample -Tumor | 0.78651071 |
| GSM414965 | Pancreatic Sample 33-Tumor | 0.702235525 |
| GSM414967 | Pancreatic Sample 27-Tumor | 0.80173908 |
| GSM414968 | Pancreatic Sample 34-Tumor | 0.876496533 |
| GSM414969 | Pancreatic Sample 35-Tumor | 0.956099378 |
| GSM414971 | Pancreatic Sample 36-Tumor | 0.920624197 |
| GSM414973 | Pancreatic Sample 37-Tumor | 1.11841996 |
| GSM414974 | Pancreatic Sample 38-Tumor | 1.238170325 |
| GSM414928 | Pancreatic Sample 16-Normal | 0.886879513 |
| GSM414930 | Pancreatic Sample 53-Normal | 0.850366035 |
| GSM414932 | Pancreatic Sample 9-Normal | 0.913529161 |
| GSM414934 | Pancreatic Sample 11-Normal | 0.966828457 |
| GSM414938 | Pancreatic Sample 54-Normal | 0.747055387 |
| GSM414940 | Pancreatic Sample 15-Normal | 1.227960395 |
| GSM414942 | Pancreatic Sample 14-Normal | 0.958522073 |
| GSM414947 | Pancreatic Sample 4-Normal | 0.945543348 |
| GSM414953 | Pancreatic Sample 24-Normal | 1.281259691 |
| GSM414955 | Pancreatic Sample 25-Normal | 1.042970307 |
| GSM414957 | Pancreatic Sample 30-Normal | 0.958522073 |
| GSM414963 | Pancreatic Sample 31-Normal | 0.983441224 |
| GSM414966 | Pancreatic Sample 33-Normal | 0.940178809 |
| GSM414970 | Pancreatic Sample 35-Normal | 1.078272438 |
| GSM414972 | Pancreatic Sample 36-Normal | 1.184178831 |
| GSM414975 | Pancreatic Sample 38-Normal | 1.034490874 |

| DBP |  |  |  |
| --- | --- | --- | --- |
| Sample | Title | FC(209782_s_at) | FC(209783_at) |
| GSM414924 | Pancreatic Sample 6-Tumor | 1.295008012 | 0.894072969 |
| GSM414925 | Pancreatic Sample 7-Tumor | 1.604551895 | 1.172928252 |
| GSM414926 | Pancreatic Sample 1-Tumor | 0.706819456 | 0.929879289 |
| GSM414927 | Pancreatic Sample 16-Tumor | 0.833451045 | 0.923369049 |
| GSM414929 | Pancreatic Sample 53-Tumor | 1.070988731 | 0.897328089 |
| GSM414931 | Pancreatic Sample 9-Tumor | 1.188516087 | 0.919028889 |
| GSM414933 | Pancreatic Sample 11-Tumor | 0.530804306 | 1.087210091 |
| GSM414935 | Pancreatic Sample 12-Tumor | 0.904077813 | 0.97328089 |
| GSM414936 | Pancreatic Sample 13-Tumor | 0.978291097 | 0.96134545 |
| GSM414937 | Pancreatic Sample 54-Tumor | 1.118441091 | 0.853926489 |
| GSM414939 | Pancreatic Sample 15-Tumor | 0.967807436 | 0.871287129 |
| GSM414941 | Pancreatic Sample 14-Tumor | 0.691093965 | 0.704190967 |
| GSM414943 | Pancreatic Sample 17-Tumor | 0.609983535 | 1.03729825 |
| GSM414944 | Pancreatic Sample 20-Tumor | 0.61494948 | 0.868032009 |
| GSM414945 | Pancreatic Sample 5-Tumor | 1.063815699 | 0.932049369 |
| GSM414946 | Pancreatic Sample 4-Tumor | 0.639779203 | 0.886477689 |
| GSM414948 | Pancreatic Sample 19-Tumor | 2.553599096 | 0.908178489 |
| GSM414949 | Pancreatic Sample 21-Tumor | 0.887524664 | 0.656449207 |
| GSM414950 | Pancreatic Sample 22-Tumor | 1.48426568 | 0.859351689 |
| GSM414951 | Pancreatic Sample 23-Tumor | 0.68281739 | 0.904923369 |
| GSM414952 | Pancreatic Sample 24-Tumor | 2.150529921 | 0.878882409 |
| GSM414954 | Pancreatic Sample 25-Tumor | 0.597568673 | 0.889732809 |
| GSM414956 | Pancreatic Sample 30-Tumor | 1.215828783 | 1.117591211 |
| GSM414958 | Pancreatic Sample 26-Tumor | 1.082575935 | 1.252136173 |
| GSM414959 | Pancreatic Sample 28-Tumor | 1.408672967 | 1.00474705 |
| GSM414960 | Pancreatic Sample 32-Tumor | 0.829036872 | 1.01776753 |
| GSM414961 | Pancreatic Sample 29-Tumor | 1.023260485 | 0.99932185 |
| GSM414962 | Pancreatic Sample 31-Tumor | 0.839244647 | 1.943306659 |
| GSM414964 | Pancreatic Sample -Tumor | 0.633157944 | 0.885392649 |
| GSM414965 | Pancreatic Sample 33-Tumor | 1.476264992 | 0.99606673 |
| GSM414967 | Pancreatic Sample 27-Tumor | 1.705526103 | 0.939644649 |
| GSM414968 | Pancreatic Sample 34-Tumor | 0.778273882 | 0.926624169 |
| GSM414969 | Pancreatic Sample 35-Tumor | 1.247555652 | 0.919028889 |
| GSM414971 | Pancreatic Sample 36-Tumor | 0.661574182 | 0.95375017 |
| GSM414973 | Pancreatic Sample 37-Tumor | 1.106302115 | 1.00583209 |
| GSM414974 | Pancreatic Sample 38-Tumor | 2.068591835 | 0.945069849 |
| GSM414928 | Pancreatic Sample 16-Normal | 0.726131463 | 0.96026041 |
| GSM414930 | Pancreatic Sample 53-Normal | 0.269816326 | 1.086125051 |
| GSM414932 | Pancreatic Sample 9-Normal | 0.709026542 | 0.903838329 |
| GSM414934 | Pancreatic Sample 11-Normal | 0.60639702 | 0.926624169 |
| GSM414938 | Pancreatic Sample 54-Normal | 1.674902778 | 1.125186491 |
| GSM414940 | Pancreatic Sample 15-Normal | 0.749305871 | 0.876712329 |
| GSM414942 | Pancreatic Sample 14-Normal | 0.663505383 | 1.050318731 |
| GSM414947 | Pancreatic Sample 4-Normal | 0.568324777 | 0.835480808 |
| GSM414953 | Pancreatic Sample 24-Normal | 1.109612745 | 0.844161128 |
| GSM414955 | Pancreatic Sample 25-Normal | 1.133063039 | 0.889732809 |
| GSM414957 | Pancreatic Sample 30-Normal | 0.472040628 | 1.231520412 |
| GSM414963 | Pancreatic Sample 31-Normal | 0.873730373 | 0.932049369 |
| GSM414966 | Pancreatic Sample 33-Normal | 1.092231938 | 1.093720331 |
| GSM414970 | Pancreatic Sample 35-Normal | 0.780756854 | 0.96134545 |
| GSM414972 | Pancreatic Sample 36-Normal | 0.700474082 | 1.207649532 |
| GSM414975 | Pancreatic Sample 38-Normal | 3.870677973 | 1.075274651 |

| RORA |  |  |  |  |  |  |  |  |  |
| --- | --- | --- | --- | --- | --- | --- | --- | --- | --- |
| Sample | Title | FC(210426_x_at) | FC(210479_s_at) | FC(235567_at) | FC(236266_at) | FC(239550_at) | FC(241760_x_at) | FC(240951_at) | FC(226682_at) |
| GSM414924 | Pancreatic Sample 6-Tumor | 0.830434783 | 0.536065574 | 0.685484267 | 0.605363985 | 0.749215208 | 0.78654759 | 0.892665474 | 0.483189527 |
| GSM414925 | Pancreatic Sample 7-Tumor | 1.471118012 | 1.521311475 | 1.455795003 | 0.714412025 | 0.728287408 | 0.71960737 | 0.921288014 | 0.730972925 |
| GSM414926 | Pancreatic Sample 1-Tumor | 1.164440994 | 1.090163934 | 1.111580188 | 0.931918656 | 0.775723753 | 0.789122214 | 0.892665474 | 0.615293068 |
| GSM414927 | Pancreatic Sample 16-Tumor | 0.841925466 | 1.036885246 | 1.208384468 | 0.70409667 | 0.831531217 | 0.913991472 | 1.268336315 | 1.091579887 |
| GSM414929 | Pancreatic Sample 53-Tumor | 0.560403727 | 0.599016393 | 0.661332286 | 0.604774536 | 0.726892222 | 0.769812535 | 0.894454383 | 0.623147873 |
| GSM414931 | Pancreatic Sample 9-Tumor | 0.828881988 | 0.52852459 | 0.681557116 | 0.75596817 | 0.825950471 | 0.773674471 | 0.908765653 | 0.433918477 |
| GSM414933 | Pancreatic Sample 11-Tumor | 1.138198758 | 1.07147541 | 0.797015365 | 1.044798114 | 0.823160098 | 0.778823719 | 0.923076923 | 0.387979768 |
| GSM414935 | Pancreatic Sample 12-Tumor | 1.02189441 | 0.91147541 | 0.869667665 | 0.947833775 | 0.726892222 | 0.760801352 | 0.876565295 | 0.825230586 |
| GSM414936 | Pancreatic Sample 13-Tumor | 0.732608696 | 0.638360656 | 0.604977664 | 0.586206897 | 0.657132892 | 0.697723067 | 0.876565295 | 0.622909848 |
| GSM414937 | Pancreatic Sample 54-Tumor | 0.916925466 | 0.833770492 | 0.841981248 | 0.782493369 | 0.602720614 | 0.778823719 | 0.8980322 | 0.586492115 |
| GSM414939 | Pancreatic Sample 15-Tumor | 0.845496894 | 0.829180328 | 0.876932895 | 0.730916593 | 0.738053715 | 0.755652104 | 0.876565295 | 0.692174948 |
| GSM414941 | Pancreatic Sample 14-Tumor | 0.738198758 | 0.944590164 | 0.844926611 | 0.878278809 | 0.60830136 | 0.720894682 | 0.862254025 | 0.836893782 |
| GSM414943 | Pancreatic Sample 17-Tumor | 0.887267081 | 0.724262295 | 0.755976633 | 0.730621869 | 0.726892222 | 0.756939416 | 0.894454383 | 0.4048795 |
| GSM414944 | Pancreatic Sample 20-Tumor | 0.465993789 | 0.24557377 | 0.454764125 | 0.473916888 | 0.739448901 | 0.760801352 | 0.894454383 | 0.155953585 |
| GSM414945 | Pancreatic Sample 5-Tumor | 3.880434783 | 3.331147541 | 3.21633695 | 3.619216033 | 0.818974538 | 0.795558774 | 1.194991055 | 2.52067837 |
| GSM414946 | Pancreatic Sample 4-Tumor | 1.763975155 | 1.426557377 | 2.334691473 | 1.095490716 | 0.728287408 | 0.758226728 | 0.885509839 | 1.218208866 |
| GSM414948 | Pancreatic Sample 19-Tumor | 0.781832298 | 0.566065574 | 0.756172991 | 0.518420277 | 0.739448901 | 0.755652104 | 0.905187835 | 0.560547456 |
| GSM414949 | Pancreatic Sample 21-Tumor | 1.060248447 | 0.941639344 | 1.074468607 | 1.101385205 | 0.807813045 | 0.778823719 | 1.012522361 | 1.063493008 |
| GSM414950 | Pancreatic Sample 22-Tumor | 1.320962733 | 1.224754098 | 1.053458348 | 1.253463012 | 0.717125916 | 0.765950599 | 0.914132379 | 0.983040762 |
| GSM414951 | Pancreatic Sample 23-Tumor | 0.751863354 | 0.595081967 | 0.754798488 | 0.771883289 | 0.754795954 | 0.74664092 | 0.892665474 | 0.785242487 |
| GSM414952 | Pancreatic Sample 24-Tumor | 1.218478261 | 1.262622951 | 0.767758087 | 1.2726201 | 0.717125916 | 0.711883498 | 0.881932021 | 1.348646236 |
| GSM414954 | Pancreatic Sample 25-Tumor | 0.562888199 | 0.481803279 | 0.665259437 | 0.919245505 | 0.739448901 | 0.769812535 | 0.828264758 | 0.545551919 |
| GSM414956 | Pancreatic Sample 30-Tumor | 0.523291925 | 0.567868852 | 0.428255854 | 0.687886826 | 0.657132892 | 0.763375976 | 0.88372093 | 0.486283844 |
| GSM414958 | Pancreatic Sample 26-Tumor | 0.45931677 | 0.558196721 | 0.642285602 | 0.737695255 | 0.753400767 | 0.75307748 | 0.896243292 | 0.465337697 |
| GSM414959 | Pancreatic Sample 28-Tumor | 1.036956522 | 0.82557377 | 0.669775661 | 0.848216917 | 0.745029648 | 0.774961783 | 0.908765653 | 0.46224338 |
| GSM414960 | Pancreatic Sample 32-Tumor | 0.328571429 | 0.233278689 | 0.245643316 | 0.552608311 | 0.887338682 | 1.000241371 | 0.973166369 | 0.144790241 |
| GSM414961 | Pancreatic Sample 29-Tumor | 0.597515528 | 0.499836066 | 0.480290609 | 0.594164456 | 0.92500872 | 0.79298415 | 0.923076923 | 0.648378459 |
| GSM414962 | Pancreatic Sample 31-Tumor | 0.294565217 | 0.261803279 | 0.377006529 | 0.564397289 | 1.411928845 | 0.957760077 | 1.055456172 | 0.430586135 |
| GSM414964 | Pancreatic Sample -Tumor | 0.42173913 | 0.450655738 | 0.52054391 | 0.674918951 | 0.733868155 | 0.773674471 | 0.892665474 | 0.570544481 |
| GSM414965 | Pancreatic Sample 33-Tumor | 0.830745342 | 0.718196721 | 0.662706789 | 0.837017389 | 0.6250436 | 0.744066297 | 0.8980322 | 0.608628384 |
| GSM414967 | Pancreatic Sample 27-Tumor | 1.877329193 | 2.006557377 | 0.639340239 | 1.642793988 | 0.858039763 | 0.79942071 | 0.928443649 | 0.612674799 |
| GSM414968 | Pancreatic Sample 34-Tumor | 0.894099379 | 0.916229508 | 1.289676501 | 1.531682877 | 0.742239275 | 0.745353608 | 0.8980322 | 1.189883963 |
| GSM414969 | Pancreatic Sample 35-Tumor | 0.49052795 | 0.31442623 | 0.901281233 | 0.634541704 | 0.782699686 | 0.773674471 | 0.908765653 | 0.512466528 |
| GSM414971 | Pancreatic Sample 36-Tumor | 0.285714286 | 0.423278689 | 0.312601247 | 0.470380195 | 0.870596442 | 0.803282645 | 0.928443649 | 0.435346623 |
| GSM414973 | Pancreatic Sample 37-Tumor | 0.300621118 | 0.306065574 | 0.662706789 | 0.666371942 | 0.77711894 | 0.762088664 | 0.880143113 | 0.398452841 |
| GSM414974 | Pancreatic Sample 38-Tumor | 0.309937888 | 0.337213115 | 0.626576997 | 0.856763926 | 0.969654691 | 0.883095985 | 0.942754919 | 0.585063969 |
| GSM414928 | Pancreatic Sample 16-Normal | 0.873602484 | 1.02295082 | 1.038142457 | 1.143236074 | 0.841297524 | 0.876659426 | 0.98568873 | 0.980660518 |
| GSM414930 | Pancreatic Sample 53-Normal | 1.872670807 | 1.952459016 | 1.282803986 | 2.155025052 | 0.885943495 | 1.034998793 | 0.980322004 | 0.970187444 |
| GSM414932 | Pancreatic Sample 9-Normal | 1.000621118 | 1.25 | 2.079426636 | 1.030356617 | 1.096616672 | 0.774961783 | 0.914132379 | 1.206069622 |
| GSM414934 | Pancreatic Sample 11-Normal | 0.823757764 | 0.62704918 | 0.854940847 | 0.528735632 | 0.76874782 | 0.771099847 | 0.908765653 | 0.930675394 |
| GSM414938 | Pancreatic Sample 54-Normal | 0.509161491 | 0.471967213 | 0.606352167 | 0.365458296 | 0.650156958 | 0.771099847 | 0.906976744 | 0.492472478 |
| GSM414940 | Pancreatic Sample 15-Normal | 2.013975155 | 1.783606557 | 1.967502823 | 0.893899204 | 0.726892222 | 0.741491673 | 0.8980322 | 1.300803332 |
| GSM414942 | Pancreatic Sample 14-Normal | 1.281521739 | 1.013442623 | 1.104314958 | 1.840848806 | 1.400767353 | 1.23968139 | 1.264758497 | 1.087771497 |
| GSM414947 | Pancreatic Sample 4-Normal | 1.168944099 | 1.215081967 | 0.92739679 | 0.803418803 | 1.118939658 | 0.978357068 | 1.008944544 | 1.262005356 |
| GSM414953 | Pancreatic Sample 24-Normal | 1.560559006 | 1.570983607 | 1.14849541 | 1.076628352 | 0.77711894 | 0.723469306 | 0.960644007 | 1.366498066 |
| GSM414955 | Pancreatic Sample 25-Normal | 0.754813665 | 0.929180328 | 0.804084237 | 0.822575892 | 0.613882107 | 0.738917049 | 0.991055456 | 1.223207379 |
| GSM414957 | Pancreatic Sample 30-Normal | 0.896583851 | 0.868360656 | 0.545874037 | 0.56380784 | 0.926403907 | 1.436640116 | 1.039355993 | 0.586492115 |
| GSM414963 | Pancreatic Sample 31-Normal | 0.843944099 | 0.94147541 | 0.411761818 | 1.050987327 | 0.969654691 | 0.834178132 | 0.926654741 | 1.549062779 |
| GSM414966 | Pancreatic Sample 33-Normal | 0.77810559 | 0.841803279 | 0.561975357 | 0.864132037 | 2.281130101 | 1.207498592 | 1.04293381 | 0.643141922 |
| GSM414970 | Pancreatic Sample 35-Normal | 0.805279503 | 0.892786885 | 1.64488734 | 1.259062776 | 0.792465992 | 0.884383297 | 0.935599284 | 1.172984231 |
| GSM414972 | Pancreatic Sample 36-Normal | 0.312888199 | 0.226721311 | 0.223454911 | 0.580312408 | 1.127310778 | 1.600128731 | 1.121645796 | 0.556739066 |
| GSM414975 | Pancreatic Sample 38-Normal | 0.503571429 | 0.398032787 | 0.798586226 | 1.021809608 | 1.022671782 | 1.386434951 | 1.11627907 | 0.671228801 |

**Supplementary Table S4. Predicted miRNAs with potential binding sites in the 3**′**-UTR of human BMAL1**

| **MicroRNA** | **Position** | **Programs** | **Seed match** | **Context^++^ score** |
| --- | --- | --- | --- | --- |
| hsa-miR-142 | 352-359 | TargetScan, miRanda, MicroCosm, PicTar, miRDB | 8mer | -0.73 |
| hsa-miR-135a | 118-124 | TargetScan, miRanda, MicroCosm, miRDB | 7mer-m8 | -0.32 |
| hsa-miR-135b | 118-124 | TargetScan, miRanda, MicroCosm, miRDB | 7mer-m8 | -0.30 |
| hsa-miR-27a | 65-71 | TargetScan, miRanda, PicTar | 7mer-1A | -0.13 |
| hsa-miR-27b | 65-71 | TargetScan, miRanda, PicTar | 7mer-1A | -0.13 |
| hsa-miR-448 | 316-323 | TargetScan, miRanda, MicroCosm, miRDB | 8mer | -0.33 |
| hsa-miR-141 | 193-199 | TargetScan, miRanda, PicTar, miRDB | 7mer-m8 | -0.12 |
| hsa-miR-153 | 317-323 | TargetScan, miRanda, PicTar | 7mer-1A | -0.22 |
| hsa-miR-203a | 329-335 | TargetScan, miRanda, PicTar, miRDB | 7mer-1A | -0.06 |
| hsa-miR-302b | 449-455 | TargetScan, PicTar, MicroCosm, miRDB | 7mer-m8 | -0.14 |
| hsa-miR-494 | 490-496 | TargetScan, miRanda, miRDB | 7mer-m8 | -0.15 |
| hsa-miR-200a | 193-199 | TargetScan, miRanda, PicTar, miRDB | 7mer-m8 | -0.12 |

**Supplementary Table S5. Expression of miR-135b-BMAL1-YY1 in TCGA dataset**

| **Case_ID** | **hsa-miR-135b** | **BMAL1** | **YY1** |
| --- | --- | --- | --- |
| **TCGA-2J-AAB1** | **5.8031** | **6.9477** | **10.0312** |
| **TCGA-2J-AAB4** | **6.2256** | **7.7469** | **9.9949** |
| **TCGA-2J-AAB6** | **6.8506** | **8.6971** | **10.3995** |
| **TCGA-2J-AAB8** | **6.3232** | **6.6122** | **10.1659** |
| **TCGA-2J-AAB9** | **3.5618** | **7.8** | **10.0858** |
| **TCGA-2J-AABA** | **5.1688** | **8.5112** | **9.6452** |
| **TCGA-2J-AABE** | **6.7986** | **7.3932** | **10.2752** |
| **TCGA-2J-AABF** | **6.1834** | **8.0009** | **10.0967** |
| **TCGA-2J-AABH** | **7.4509** | **7.5467** | **9.4629** |
| **TCGA-2J-AABI** | **6.1247** | **8.0207** | **9.6491** |
| **TCGA-2J-AABK** | **5.2895** | **7.9554** | **10.1832** |
| **TCGA-2J-AABO** | **5.7653** | **8.116** | **10.2246** |
| **TCGA-2J-AABP** | **1.1851** | **7.5567** | **9.818** |
| **TCGA-2J-AABR** | **4.2389** | **7.581** | **10.1177** |
| **TCGA-2J-AABT** | **4.0734** | **7.541** | **9.9261** |
| **TCGA-2J-AABU** | **6.3692** | **7.5437** | **10.4585** |
| **TCGA-2J-AABV** | **4.1342** | **6.7134** | **9.4122** |
| **TCGA-2L-AAQA** | **7.4501** | **6.8928** | **10.2607** |
| **TCGA-2L-AAQE** | **5.7782** | **7.6424** | **10.2386** |
| **TCGA-2L-AAQI** | **6.1126** | **6.8956** | **10.4036** |
| **TCGA-2L-AAQJ** | **6.7745** | **7.3031** | **10.2745** |
| **TCGA-2L-AAQL** | **6.4181** | **6.3204** | **9.1587** |
| **TCGA-2L-AAQM** | **0.8298** | **9.182** | **9.8708** |
| **TCGA-3A-A9I5** | **5.7416** | **7.4167** | **9.6219** |
| **TCGA-3A-A9I7** | **5.6342** | **7.4799** | **10.1065** |
| **TCGA-3A-A9I9** | **6.4535** | **7.1677** | **10.1116** |
| **TCGA-3A-A9IB** | **4.845** | **7.6506** | **10.4071** |
| **TCGA-3A-A9IC** | **7.8877** | **7.3441** | **9.7927** |
| **TCGA-3A-A9IH** | **6.0159** | **7.222** | **9.6294** |
| **TCGA-3A-A9IJ** | **4.6294** | **8.6206** | **9.3631** |
| **TCGA-3A-A9IL** | **0.544** | **8.1719** | **9.6389** |
| **TCGA-3A-A9IN** | **0** | **7.0701** | **9.5703** |
| **TCGA-3A-A9IO** | **0.9294** | **7.4916** | **9.6645** |
| **TCGA-3A-A9IR** | **0.5222** | **7.5777** | **9.8426** |
| **TCGA-3A-A9IS** | **0** | **8.2631** | **9.7553** |
| **TCGA-3A-A9IU** | **7.9731** | **6.9182** | **10.3792** |
| **TCGA-3A-A9IV** | **0.7049** | **7.3751** | **9.794** |
| **TCGA-3A-A9IX** | **5.2225** | **8.0665** | **10.1656** |
| **TCGA-3A-A9IZ** | **6.8925** | **7.4075** | **10.5506** |
| **TCGA-3A-A9J0** | **4.2657** | **7.9613** | **10.7867** |
| **TCGA-3E-AAAY** | **5.12** | **7.2722** | **9.887** |
| **TCGA-3E-AAAZ** | **6.1224** | **8.2998** | **10.1886** |
| **TCGA-F2-6879** | **6.5547** | **8.2933** | **10.6083** |
| **TCGA-F2-6880** | **2.3227** | **5.2872** | **10.0418** |
| **TCGA-F2-7273** | **3.4096** | **8.8574** | **10.2738** |
| **TCGA-F2-7276** | **2.5696** | **8.8407** | **10.0946** |
| **TCGA-F2-A44G** | **6.1086** | **7.3607** | **10.3129** |
| **TCGA-F2-A44H** | **6.3205** | **7.3103** | **10.1142** |
| **TCGA-F2-A7TX** | **4.7422** | **7.6422** | **10.6374** |
| **TCGA-F2-A8YN** | **7.0443** | **8.0924** | **10.5608** |
| **TCGA-FB-A4P5** | **3.9915** | **8.1138** | **9.9506** |
| **TCGA-FB-A4P6** | **4.7858** | **8.2994** | **10.0913** |
| **TCGA-FB-A545** | **6.2969** | **7.0189** | **10.4995** |
| **TCGA-FB-A5VM** | **4.7581** | **6.7541** | **9.3267** |
| **TCGA-FB-A78T** | **5.9761** | **8.3077** | **9.9363** |
| **TCGA-FB-A7DR** | **4.0025** | **7.9841** | **9.7362** |
| **TCGA-FB-AAPP** | **6.6422** | **7.6026** | **10.0986** |
| **TCGA-FB-AAPQ** | **6.6814** | **8.3796** | **10.4365** |
| **TCGA-FB-AAPS** | **5.1563** | **7.9334** | **9.783** |
| **TCGA-FB-AAPU** | **6.9797** | **7.7633** | **9.8309** |
| **TCGA-FB-AAPY** | **6.0911** | **8.0838** | **9.9492** |
| **TCGA-FB-AAPZ** | **6.6662** | **7.6961** | **10.3548** |
| **TCGA-FB-AAQ0** | **6.8002** | **7.4022** | **10.1595** |
| **TCGA-FB-AAQ1** | **6.1849** | **8.5782** | **10.2889** |
| **TCGA-FB-AAQ2** | **6.1595** | **7.9921** | **10.5644** |
| **TCGA-FB-AAQ3** | **6.0484** | **7.5194** | **10.7188** |
| **TCGA-FB-AAQ6** | **7.2509** | **6.3859** | **10.5026** |
| **TCGA-H6-8124** | **5.9043** | **8.0745** | **10.4293** |
| **TCGA-H6-A45N** | **4.8271** | **8.335** | **9.8331** |
| **TCGA-H8-A6C1** | **6.1169** | **7.2363** | **9.9326** |
| **TCGA-HV-A5A3** | **7.2319** | **7.3825** | **10.2149** |
| **TCGA-HV-A5A4** | **7.055** | **7.1255** | **9.9031** |
| **TCGA-HV-A5A5** | **5.5094** | **7.9856** | **10.022** |
| **TCGA-HV-A5A6** | **7.3025** | **7.5938** | **10.2148** |
| **TCGA-HV-A7OL** | **7.1407** | **7.1127** | **9.5417** |
| **TCGA-HV-A7OP** | **8.412** | **5.7305** | **8.8507** |
| **TCGA-HV-AA8V** | **5.5287** | **7.6909** | **9.9616** |
| **TCGA-HV-AA8X** | **7.2427** | **7.1921** | **10.6719** |
| **TCGA-HZ-7289** | **4.9888** | **7.5109** | **10.2196** |
| **TCGA-HZ-7918** | **4.5478** | **8.8516** | **10.3479** |
| **TCGA-HZ-7919** | **5.8926** | **7.2013** | **10.3861** |
| **TCGA-HZ-7920** | **2.0797** | **9.1607** | **9.9955** |
| **TCGA-HZ-7922** | **4.4199** | **8.0348** | **10.6933** |
| **TCGA-HZ-7923** | **3.4945** | **8.217** | **9.971** |
| **TCGA-HZ-7924** | **4.8969** | **8.518** | **10.2916** |
| **TCGA-HZ-7925** | **5.195** | **7.297** | **10.0613** |
| **TCGA-HZ-7926** | **4.6728** | **9.4648** | **10.3992** |
| **TCGA-HZ-8001** | **3.3421** | **8.7728** | **9.707** |
| **TCGA-HZ-8002** | **3.0639** | **9.0057** | **10.2813** |
| **TCGA-HZ-8003** | **3.9326** | **7.7861** | **10.2114** |
| **TCGA-HZ-8005** | **3.9876** | **9.1575** | **10.227** |
| **TCGA-HZ-8315** | **5.4081** | **7.8196** | **10.1743** |
| **TCGA-HZ-8317** | **4.5966** | **8.0193** | **10.1232** |
| **TCGA-HZ-8519** | **3.535** | **8.7409** | **9.9337** |
| **TCGA-HZ-8636** | **5.6416** | **7.9096** | **10.5882** |
| **TCGA-HZ-8637** | **4.6737** | **8.0784** | **10.2213** |
| **TCGA-HZ-8638** | **4.3879** | **8.3744** | **10.1412** |
| **TCGA-HZ-A49G** | **4.9539** | **8.3485** | **9.8694** |
| **TCGA-HZ-A49H** | **5.1486** | **7.9225** | **9.6441** |
| **TCGA-HZ-A49I** | **5.9851** | **8.4159** | **9.8012** |
| **TCGA-HZ-A4BH** | **5.1181** | **7.8867** | **10.1931** |
| **TCGA-HZ-A4BK** | **6.0627** | **9.0885** | **10.2797** |
| **TCGA-HZ-A77O** | **5.2772** | **8.2756** | **10.2208** |
| **TCGA-HZ-A77P** | **2.9552** | **8.0383** | **9.8238** |
| **TCGA-HZ-A77Q** | **6.1629** | **7.6494** | **10.0551** |
| **TCGA-HZ-A8P0** | **7.1741** | **8.4867** | **10.1285** |
| **TCGA-HZ-A8P1** | **8.22** | **7.5486** | **10.1022** |
| **TCGA-HZ-A9TJ** | **5.6877** | **7.5699** | **10.2673** |
| **TCGA-IB-7644** | **5.4155** | **7.5695** | **10.5745** |
| **TCGA-IB-7645** | **5.0138** | **8.1963** | **10.0465** |
| **TCGA-IB-7646** | **4.1599** | **7.5015** | **10.5845** |
| **TCGA-IB-7647** | **4.7823** | **8.2476** | **10.3411** |
| **TCGA-IB-7649** | **4.9339** | **8.4577** | **10.374** |
| **TCGA-IB-7651** | **5.2196** | **7.8414** | **10.5609** |
| **TCGA-IB-7652** | **5.8146** | **7.3645** | **10.4199** |
| **TCGA-IB-7654** | **4.9486** | **7.9541** | **10.2724** |
| **TCGA-IB-7885** | **5.2737** | **7.4306** | **10.0301** |
| **TCGA-IB-7886** | **5.6641** | **7.8738** | **10.4567** |
| **TCGA-IB-7887** | **5.7989** | **8.3951** | **10.3256** |
| **TCGA-IB-7888** | **3.3718** | **7.6215** | **9.8361** |
| **TCGA-IB-7889** | **4.9415** | **7.6617** | **9.9601** |
| **TCGA-IB-7890** | **4.3277** | **7.8714** | **10.4007** |
| **TCGA-IB-7891** | **4.1291** | **7.1851** | **10.1966** |
| **TCGA-IB-7893** | **4.06** | **8.3862** | **10.576** |
| **TCGA-IB-7897** | **1.3976** | **7.5672** | **10.1709** |
| **TCGA-IB-8126** | **3.3479** | **7.794** | **10.1099** |
| **TCGA-IB-8127** | **5.8771** | **7.7703** | **10.7275** |
| **TCGA-IB-A5SO** | **4.4479** | **7.5645** | **9.9345** |
| **TCGA-IB-A5SP** | **6.1843** | **7.8515** | **10.0913** |
| **TCGA-IB-A5SQ** | **4.8277** | **7.5564** | **10.07** |
| **TCGA-IB-A5SS** | **6.0602** | **7.4021** | **10.0799** |
| **TCGA-IB-A5ST** | **4.0247** | **7.8641** | **9.843** |
| **TCGA-IB-A6UF** | **6.213** | **7.5726** | **10.4579** |
| **TCGA-IB-A6UG** | **5.4964** | **7.4812** | **10.0868** |
| **TCGA-IB-A7LX** | **6.8583** | **7.6159** | **10.7251** |
| **TCGA-IB-A7M4** | **6.007** | **7.2685** | **10.9381** |
| **TCGA-IB-AAUM** | **5.6847** | **7.3296** | **10.018** |
| **TCGA-IB-AAUN** | **7.1313** | **7.4533** | **9.7127** |
| **TCGA-IB-AAUO** | **5.4782** | **7.9219** | **10.1858** |
| **TCGA-IB-AAUP** | **4.6081** | **8.4472** | **10.0796** |
| **TCGA-IB-AAUQ** | **3.9779** | **7.8087** | **9.9062** |
| **TCGA-IB-AAUR** | **5.5554** | **8.0777** | **9.931** |
| **TCGA-IB-AAUS** | **3.8025** | **7.4667** | **9.8052** |
| **TCGA-IB-AAUT** | **4.5675** | **8.6479** | **9.8165** |
| **TCGA-IB-AAUU** | **7.0471** | **7.4286** | **10.2949** |
| **TCGA-IB-AAUV** | **1.9374** | **7.9981** | **10.0669** |
| **TCGA-IB-AAUW** | **4.0887** | **7.4944** | **9.934** |
| **TCGA-L1-A7W4** | **7.0993** | **6.8138** | **10.5853** |
| **TCGA-LB-A7SX** | **6.6065** | **7.5963** | **9.7359** |
| **TCGA-LB-A8F3** | **9.2784** | **7.8703** | **9.3661** |
| **TCGA-LB-A9Q5** | **5.2729** | **8.4205** | **9.961** |
| **TCGA-M8-A5N4** | **7.1742** | **7.6949** | **9.9959** |
| **TCGA-OE-A75W** | **6.7362** | **7.7648** | **9.622** |
| **TCGA-PZ-A5RE** | **6.8276** | **8.0105** | **9.8743** |
| **TCGA-Q3-A5QY** | **5.1867** | **8.0915** | **10.0655** |
| **TCGA-Q3-AA2A** | **6.9386** | **7.58** | **9.9672** |
| **TCGA-RB-A7B8** | **6.2813** | **8.1043** | **9.8821** |
| **TCGA-RB-AA9M** | **4.3936** | **8.6162** | **10.229** |
| **TCGA-RL-AAAS** | **3.4675** | **7.5131** | **9.5593** |
| **TCGA-S4-A8RM** | **6.4076** | **7.6511** | **10.2058** |
| **TCGA-S4-A8RO** | **5.7878** | **6.3697** | **10.3884** |
| **TCGA-S4-A8RP** | **7.5846** | **7.5937** | **9.843** |
| **TCGA-US-A774** | **4.8147** | **7.4081** | **9.8484** |
| **TCGA-US-A776** | **4.6875** | **7.7489** | **10.0179** |
| **TCGA-US-A779** | **5.7667** | **6.9056** | **9.7183** |
| **TCGA-US-A77E** | **6.3529** | **8.4598** | **10.036** |
| **TCGA-US-A77G** | **6.5668** | **7.8347** | **10.4445** |
| **TCGA-US-A77J** | **3.4334** | **7.726** | **9.3834** |
| **TCGA-XD-AAUG** | **3.9712** | **8.0764** | **9.8778** |
| **TCGA-XD-AAUH** | **3.8549** | **8.4503** | **9.6524** |
| **TCGA-XD-AAUI** | **4.5103** | **8.5937** | **10.1934** |
| **TCGA-XD-AAUL** | **6.0693** | **8.649** | **9.991** |
| **TCGA-XN-A8T3** | **6.075** | **7.9159** | **10.2494** |
| **TCGA-XN-A8T5** | **4.9208** | **8.2094** | **9.6181** |
| **TCGA-YB-A89D** | **5.7656** | **6.958** | **10.0818** |
| **TCGA-YH-A8SY** | **6.4024** | **8.3708** | **10.0937** |
| **TCGA-YY-A8LH** | **8.1893** | **7.5442** | **9.9737** |
| **TCGA-Z5-AAPL** | **5.824** | **7.9459** | **10.1954** |

**Supplementary Table S6. Circadian parameters of clock genes in normal and malignant pancreatic epithelial cells**

| **Cell line** | **Gene** | **BH. Q** | **Adj. *P*** | **Period (h)** | **Phase (h)** | **Amplitude** |
| --- | --- | --- | --- | --- | --- | --- |
| HPDE6c7 | *BMAL1* | <0.001 | <0.001 | 24 | 8 | 0.707 |
|  | *CLOCK* | <0.001 | <0.001 | 16 | 8 | 0.396 |
|  | *PER1* | <0.001 | <0.001 | 28 | 20 | 0.856 |
|  | *PER2* | <0.001 | <0.001 | 28 | 20 | 1.195 |
|  | *CRY1* | <0.001 | <0.001 | 20 | 6 | 0.410 |
|  | *CRY2* | <0.001 | <0.001 | 28 | 22 | 0.849 |
|  | *NR1D1* | <0.001 | <0.001 | 20 | 12 | 0.557 |
|  | *RORA* | <0.001 | <0.001 | 12 | 6 | 0.785 |
|  | *SIRT1* | 0.002 | <0.001 | 16 | 6 | 0.400 |
|  |  |  |  |  |  |  |
| HPDE6c7 + | *BMAL1* | - | - | - | - | - |
| miR-135b | *CLOCK* | - | - | - | - | - |
|  | *PER1* | 0.015 | 0.007 | 28 | 20 | 0.205 |
|  | *PER2* | 0.022 | 0.011 | 28 | 22 | 0.290 |
|  | *CRY1* | - | - | - | - | - |
|  | *CRY2* | - | - | - | - | - |
|  | *NR1D1* | 0.913 | 0.609 | - | - | - |
|  | *RORA* | 0.143 | 0.110 | - | - | - |
|  | *SIRT1* | - | - | - | - | - |
|  |  |  |  |  |  |  |
| MIA PaCa-2 | *BMAL1* | 0.016 | 0.009 | 12 | 7 | 0.430 |
|  | *CLOCK* | 0.513 | 0.375 | - | - | - |
|  | *PER1* | 0.075 | 0.040 | 22 | 1 | 0.151 |
|  | *PER2* | 0.041 | 0.023 | 24 | 23 | 0.223 |
|  | *CRY1* | 0.867 | 0.582 | - | - | - |
|  | *CRY2* | 0.028 | 0.016 | 22 | 19 | 0.092 |
|  | *NR1D1* | 0.079 | 0.048 | 12 | 10 | 0.269 |
|  | *RORA* | 0.365 | 0.283 | - | - | - |
|  | *SIRT1* | 0.242 | 0.167 | - | - | - |
|  |  |  |  |  |  |  |
| AsPC-1 | *BMAL1* | 0.030 | 0.017 | 16 | 6 | 0.244 |
|  | *CLOCK* | - | - | - | - | - |
|  | *PER1* | - | - | - | - | - |
|  | *PER2* | 0.071 | 0.045 | 28 | 13 | 0.115 |
|  | *CRY1* | 0.543 | 0.385 | - | - | - |
|  | *CRY2* | 0.351 | 0.207 | - | - | - |
|  | *NR1D1* | 0.043 | 0.024 | 20 | 11 | 0.218 |
|  | *RORA* | - | - | - | - | - |
|  | *SIRT1* | - | - | - | - | - |
|  |  |  |  |  |  |  |
| SW1990 | *BMAL1* | 0.406 | 0.290 | - | - | - |
|  | *CLOCK* | - | - | - | - | - |
|  | *PER1* | 0.258 | 0.162 | - | - | - |
|  | *PER2* | 0.841 | 0.516 | - | - | - |
|  | *CRY1* | - | - | - | - | - |
|  | *CRY2* | - | - | - | - | - |
|  | *NR1D1* | - | - | - | - | - |
|  | *RORA* | 0.877 | 0.696 | - | - | - |
|  | *SIRT1* | - | - | - | - | - |
|  |  |  |  |  |  |  |
| Panc-1 | *BMAL1* | 0.563 | 0.360 | - | - | - |
|  | *CLOCK* | - | - | - | - | - |
|  | *PER1* | - | - | - | - | - |
|  | *PER2* | - | - | - | - | - |
|  | *CRY1* | - | - | - | - | - |
|  | *CRY2* | 0.233 | 0.136 | - | - | - |
|  | *NR1D1* | - | - | - | - | - |
|  | *RORA* | - | - | - | - | - |
|  | *SIRT1* | - | - | - | - | - |
|  |  |  |  |  |  |  |
| Panc-1 + | *BMAL1* | <0.001 | <0.001 | 20 | 14 | 0.806 |
| Anti-miR-135b | *CLOCK* | 0.103 | 0.063 | - | - | - |
|  | *PER1* | <0.001 | <0.001 | 24 | 4 | 0.481 |
|  | *PER2* | <0.001 | <0.001 | 12 | 10 | 0.559 |
|  | *CRY1* | <0.001 | <0.001 | 24 | 0 | 0.389 |
|  | *CRY2* | <0.001 | <0.001 | 20 | 2 | 0.350 |
|  | *NR1D1* | 0.425 | 0.360 | - | - | - |
|  | *RORA* | 0.014 | 0.007 | 24 | 20 | 0.636 |
|  | *SIRT1* | 0.405 | 0.248 | - | - | - |

*P* ≥ 0.05 was considered arrhythmic.

**Supplementary Table S7. Correlation between expression of the miR-135b-BMAL1-YY1 regulatory loop and clinicopathological characteristics in 55 patients (Cohort 1) with pancreatic cancer**

| **Characteristics** | **Total**  **n=55 (%)** | **miR-135b expression** | | ****P*** | **BMAL1 expression** | | ****P*** | **YY1 expression** | | ****P*** |
| --- | --- | --- | --- | --- | --- | --- | --- | --- | --- | --- |
|  |  | **Low**  **n=24 (%)** | **High**  **n=31 (%)** |  | **Low**  **n=29 (%)** | **High**  **n=26 (%)** |  | **Low**  **n=27 (%)** | **High**  **n=28 (%)** |  |
| Age |  |  |  |  |  |  |  |  |  |  |
| <60 | 24 (43.6) | 14 (58.3) | 10 (32.3) | 0.053 | 13 (44.8) | 11 (42.3) | 0.851 | 14 (51.9) | 10 (35.7) | 0.228 |
| ≥60 | 31 (56.4) | 10 (41.7) | 21 (67.7) |  | 16 (55.2) | 15 (57.7) |  | 13 (48.1) | 18 (64.3) |  |
| Gender |  |  |  |  |  |  |  |  |  |  |
| Male | 32 (58.2) | 15 (62.5) | 17 (54.8) | 0.568 | 16 (55.2) | 16 (61.5) | 0.633 | 18 (66.7) | 14 (50.0) | 0.210 |
| Female | 23 (41.8) | 9 (37.5) | 14 (45.2) |  | 13 (44.8) | 10 (38.5) |  | 9 (33.3) | 14 (50.0) |  |
| Tumor location |  |  |  |  |  |  |  |  |  |  |
| Head | 38 (69.1) | 13 (54.2) | 25 (80.6) | 0.035* | 20 (69.0) | 18 (69.2) | 0.983 | 19 (70.4) | 19 (67.9) | 0.840 |
| Body/tail | 17 (30.9) | 11 (45.8) | 6 (19.4) |  | 9 (31.0) | 8 (30.8) |  | 8 (29.6) | 9 (32.1) |  |
| T classification |  |  |  |  |  |  |  |  |  |  |
| T1-T2 | 16 (29.1) | 11 (45.8) | 5 (16.1) | 0.016* | 2 (6.9) | 14 (53.8) | <0.001* | 12 (44.4) | 4 (14.3) | 0.014* |
| T3-T4 | 39 (70.9) | 13 (54.2) | 26 (83.9) |  | 27 (93.1) | 12 (46.2) |  | 15 (55.6) | 24 (85.7) |  |
| N classification |  |  |  |  |  |  |  |  |  |  |
| Absent | 20 (36.4) | 12 (50.0) | 8 (25.8) | 0.064 | 7 (24.1) | 13 (50.0) | 0.047* | 12 (44.4) | 8 (28.6) | 0.221 |
| Present | 35 (63.6) | 12 (50.0) | 23 (74.2) |  | 22 (75.9) | 13 (50.0) |  | 15 (55.6) | 20 (71.4) |  |
| AJCC stage |  |  |  |  |  |  |  |  |  |  |
| I-IIA | 17 (30.9) | 11 (45.8) | 6 (19.4) | 0.035* | 4 (13.8) | 13 (50.0) | 0.004* | 10 (37.0) | 7 (25.0) | 0.334 |
| IIB-IV | 38 (69.1) | 13 (54.2) | 25 (80.6) |  | 25 (86.2) | 13 (50.0) |  | 17 (63.0) | 21 (75.0) |  |
| Histological grade |  |  |  |  |  |  |  |  |  |  |
| Well/Moderate | 39 (70.9) | 23 (95.8) | 16 (51.6) | <0.001* | 17 (58.6) | 22 (84.6) | 0.034* | 23 (85.2) | 16 (57.1) | 0.022* |
| Poor | 16 (29.1) | 1 (4.2) | 15 (48.4) |  | 12 (41.4) | 4 (15.4) |  | 4 (14.8) | 12 (42.9) |  |
| CA19-9 (U/mL) |  |  |  |  |  |  |  |  |  |  |
| <37 | 10 (18.2) | 9 (37.5) | 1 (3.2) | 0.001* | 4 (13.8) | 6 (23.1) | 0.525 | 6 (22.2) | 4 (14.3) | 0.439 |
| ≥37 | 41 (74.5) | 14 (58.3) | 27 (87.1) |  | 21 (72.4) | 20 (76.9) |  | 19 (70.4) | 22 (78.6) |  |
| Missing | 4 (7.3) |  |  |  |  |  |  |  |  |  |
| BMAL1 expression |  |  |  |  |  |  |  |  |  |  |
| Low | 29 (52.7) | 7 (29.2) | 22 (71.0) | 0.002* |  |  |  |  |  |  |
| High | 26 (47.3) | 17 (70.8) | 9 (29.0) |  |  |  |  |  |  |  |
| YY1 expression |  |  |  |  |  |  |  |  |  |  |
| Low | 27 (49.1) | 17 (70.8) | 10 (32.3) | 0.005* | 11 (37.9) | 16 (61.5) | 0.080 |  |  |  |
| High | 28 (50.9) | 7 (29.2) | 21 (67.7) |  | 18 (62.1) | 10 (38.5) |  |  |  |  |

* *P* < 0.05 indicates a significant relationship among the variables.

**Supplementary Table S8. Clinical data of 141 pancreatic cancer patients from TCGA cohort**

| case_id | X_EVENT | OS (year) | age | gender | anatomic_neoplasm_subdivision | neoplasm_histologic_grade | pathologic_T | pathologic_N | pathologic_stage |
| --- | --- | --- | --- | --- | --- | --- | --- | --- | --- |
| TCGA-2J-AAB1 | 1 | 0.180821918 | 65 | MALE | Head of Pancreas | G3 | T3 | N1 | Stage IIB |
| TCGA-2J-AAB4 | 0 | 1.997260274 | 48 | MALE | Body & Tail of Pancreas | G2 | T2 | N1 | Stage IIB |
| TCGA-2J-AAB6 | 1 | 0.802739726 | 75 | MALE | Body of Pancreas | G2 | T3 | N0 | Stage IIA |
| TCGA-2J-AAB8 | 0 | 0.219178082 | 71 | MALE | Head of Pancreas | G3 | T3 | N1 | Stage IIB |
| TCGA-2J-AAB9 | 1 | 1.717808219 | 70 | FEMALE | Head of Pancreas | G1 | T3 | N1 | Stage IIB |
| TCGA-2J-AABA | 1 | 1.663013699 | 55 | MALE | Head of Pancreas | G2 | T3 | N1 | Stage IIB |
| TCGA-2J-AABE | 0 | 1.852054795 | 73 | MALE | Body of Pancreas | G2 | T3 | N0 | Stage IIA |
| TCGA-2J-AABF | 1 | 1.893150685 | 73 | MALE | Head of Pancreas | G3 | T3 | N1 | Stage IIB |
| TCGA-2J-AABH | 0 | 1.854794521 | 61 | MALE | Body of Pancreas | G3 | T3 | N0 | Stage IIA |
| TCGA-2J-AABK | 0 | 1.326027397 | 71 | MALE | Uncinate process | G2 | T3 | N1 | Stage IIB |
| TCGA-2J-AABO | 0 | 1.205479452 | 43 | MALE | Head of Pancreas | G2 | T3 | N1 | Stage IIB |
| TCGA-2J-AABP | 0 | 0.98630137 | 58 | FEMALE | Tail of Pancreas | G4 | T3 | N1 | Stage IIB |
| TCGA-2J-AABR | 0 | 1.2 | 60 | FEMALE | Head & body of pancreas | G3 | T3 | N0 | Stage IIA |
| TCGA-2J-AABT | 0 | 0.873972603 | 72 | FEMALE | Head of Pancreas | G2 | T3 | N1 | Stage IIB |
| TCGA-2J-AABU | 1 | 0.75890411 | 56 | MALE | Head of Pancreas | G3 | T3 | N1 | Stage IIB |
| TCGA-2J-AABV | 1 | 1.78630137 | 74 | MALE | Head of Pancreas | G4 | T3 | N1 | Stage IIB |
| TCGA-2L-AAQA | 1 | 0.391780822 | 76 | MALE | Head of Pancreas | G2 | T3 | N1 | Stage IIB |
| TCGA-2L-AAQE | 1 | 1.873972603 | 56 | MALE | Head of Pancreas | G2 | T3 | N1 | Stage IIB |
| TCGA-2L-AAQI | 1 | 0.282191781 | 66 | MALE | Head of Pancreas | G3 | T3 | N1 | Stage IIB |
| TCGA-2L-AAQJ | 1 | 1.079452055 | 49 | FEMALE | Head of Pancreas | G2 | T4 | N0 | Stage III |
| TCGA-2L-AAQL | 1 | 0.8 | 82 | MALE | Head of Pancreas | G3 | T3 | N1 | Stage IIB |
| TCGA-2L-AAQM | 0 | 3.789041096 | 52 | MALE | Panreatic body and tail, spread to stomach | G1 | T3 | N1 | Stage IIB |
| TCGA-3A-A9I9 | 1 | 1.736986301 | 67 | MALE | Head of Pancreas | G2 | T3 | N0 | Stage IIA |
| TCGA-3A-A9IB | 1 | 0.61369863 | 69 | FEMALE | Head of Pancreas | G3 | T3 | N1 | Stage IIB |
| TCGA-3A-A9IC | 1 | 2.021917808 | 61 | FEMALE | Head of Pancreas | G2 | T3 | N1 | Stage IIB |
| TCGA-3A-A9IJ | 0 | 5.079452055 | 65 | MALE | Head of Pancreas | G1 | T2 | N0 | Stage IB |
| TCGA-3A-A9IL | 0 | 7.008219178 | 39 | FEMALE | Head of Pancreas | G1 | T1 |  | Stage I |
| TCGA-3A-A9IS | 0 | 2.553424658 | 67 | MALE | Body of Pancreas | G1 | T2 | N0 | Stage IB |
| TCGA-3A-A9IU | 1 | 1.254794521 | 65 | MALE | Head of Pancreas | G3 | T3 | N1 | Stage IIB |
| TCGA-3A-A9IV | 0 | 2.649315068 | 59 | FEMALE | Tail of Pancreas | G1 | T2 | N0 | Stage IB |
| TCGA-3A-A9IZ | 1 | 0.843835616 | 47 | MALE | Head of Pancreas | G2 | T3 | N1 | Stage IIB |
| TCGA-3A-A9J0 | 0 | 1.032876712 | 75 | MALE | Head of Pancreas | G2 | T3 | N1 | Stage IIB |
| TCGA-3E-AAAZ | 1 | 5.978082192 | 71 | MALE | Head of Pancreas | G2 | T3 | N0 | Stage IIA |
| TCGA-F2-6879 | 1 | 0.915068493 | 57 | MALE | Head of Pancreas | G2 | T3 | N1 | Stage IIB |
| TCGA-F2-6880 | 0 | 0.808219178 | 70 | MALE | Head of Pancreas | G1 | T3 | N1 | Stage IIB |
| TCGA-F2-7273 | 0 | 0.98630137 | 54 | MALE | Head of Pancreas | G3 | T3 | N1 | Stage IIB |
| TCGA-F2-7276 | 1 | 0.591780822 | 64 | MALE | Head of Pancreas | G1 | T3 | N1 | Stage IIB |
| TCGA-F2-A44G | 1 | 0.638356164 | 79 | FEMALE | Head of Pancreas | G2 | T3 | N1 | Stage IIB |
| TCGA-F2-A44H | 0 | 1.605479452 | 65 | MALE | Head of Pancreas | G2 | T3 | N0 | Stage IIA |
| TCGA-F2-A7TX | 1 | 0.260273973 | 77 | MALE | Head of Pancreas | G3 | T3 | N1 | Stage IIB |
| TCGA-F2-A8YN | 0 | 1.416438356 | 76 | MALE | Head of Pancreas | G2 | T3 | N0 | Stage IIA |
| TCGA-FB-A4P6 | 0 | 0.994520548 | 54 | MALE | Head of Pancreas | G1 | T3 | N1 | Stage IIB |
| TCGA-FB-A545 | 0 | 1.054794521 | 72 | FEMALE | Body of Pancreas | G2 | T3 | N1 | Stage IIB |
| TCGA-FB-A5VM | 0 | 1.230136986 | 74 | MALE | Head of Pancreas | G3 | T2 | N0 | Stage IB |
| TCGA-FB-A78T | 0 | 1.035616438 | 71 | FEMALE | Head of Pancreas | G2 | T3 | N1 | Stage IIB |
| TCGA-FB-A7DR | 1 | 0.967123288 | 48 | MALE | Head of Pancreas | G2 | T3 | NX | Stage IIA |
| TCGA-FB-AAPP | 1 | 1.328767123 | 71 | MALE | Head of Pancreas | G3 | T3 | N1 | Stage IIB |
| TCGA-FB-AAPQ | 1 | 3.095890411 | 65 | MALE | Head of Pancreas | G2 | T3 | N1 | Stage IIB |
| TCGA-FB-AAPU | 1 | 1.043835616 | 41 | FEMALE | Head of Pancreas | G2 | T3 | N0 | Stage IIA |
| TCGA-FB-AAPY | 1 | 2.901369863 | 71 | MALE | Head of Pancreas | G2 | T2 | N1 | Stage IIB |
| TCGA-FB-AAQ0 | 1 | 1.295890411 | 68 | MALE | Head of Pancreas | G3 | T3 | N0 | Stage IIA |
| TCGA-FB-AAQ2 | 1 | 0.419178082 | 81 | FEMALE | Head of Pancreas | G3 | T3 | N1 | Stage IIB |
| TCGA-FB-AAQ3 | 1 | 0.084931507 | 65 | FEMALE | Head of Pancreas | G2 | T3 | N1 | Stage IIB |
| TCGA-FB-AAQ6 | 1 | 0.668493151 | 85 | MALE | Tail of Pancreas | G2 | T1 | N0 | Stage IA |
| TCGA-H6-8124 | 0 | 1.073972603 | 56 | FEMALE | Head of Pancreas | G3 | T3 | N1 | Stage IIB |
| TCGA-H6-A45N | 1 | 1.153424658 | 88 | FEMALE | Tail of Pancreas | G3 | T3 | N1 | Stage IIB |
| TCGA-H8-A6C1 | 0 | 1.084931507 | 53 | MALE | Head of Pancreas | G2 | T3 | N0 | Stage IIA |
| TCGA-HZ-7289 | 1 | 1.810958904 | 77 | MALE | Head of Pancreas | G1 | T3 | N1 | Stage IIB |
| TCGA-HZ-7918 | 0 | 0.076712329 | 72 | MALE | Head of Pancreas | G3 | T3 | N1 | Stage IIB |
| TCGA-HZ-7919 | 1 | 1.624657534 | 52 | FEMALE | Head of Pancreas | G2 | T3 | N1 | Stage IIB |
| TCGA-HZ-7920 | 1 | 0.646575342 | 71 | MALE | Head of Pancreas | G2 | T2 | N0 | Stage IB |
| TCGA-HZ-7922 | 0 | 0.010958904 | 61 | FEMALE | Head of Pancreas | G1 | T3 | N1 | Stage IIB |
| TCGA-HZ-7923 | 0 | 0.021917808 | 65 | MALE | Head of Pancreas | G2 | T3 | N0 | Stage IIA |
| TCGA-HZ-7924 | 0 | 2.301369863 | 60 | FEMALE | Head of Pancreas | G2 | T3 | N0 | Stage IIA |
| TCGA-HZ-7925 | 1 | 1.682191781 | 66 | MALE | Head of Pancreas | G2 | T3 | N1 | Stage IIB |
| TCGA-HZ-7926 | 1 | 1.419178082 | 57 | MALE | Head of Pancreas | G1 | T3 | N1 | Stage IIB |
| TCGA-HZ-8001 | 0 | 1.934246575 | 69 | MALE | Head of Pancreas | G2 | T4 | N0 | Stage III |
| TCGA-HZ-8002 | 1 | 1.002739726 | 61 | MALE | Head of Pancreas | G2 | T3 | N1 | Stage IIB |
| TCGA-HZ-8003 | 0 | 0.057534247 | 65 | FEMALE | Head of Pancreas | G2 | T3 | N1 | Stage IIB |
| TCGA-HZ-8315 | 1 | 0.819178082 | 54 | FEMALE | Head of Pancreas | G2 | T3 | N0 | Stage IIA |
| TCGA-HZ-8317 | 1 | 1.035616438 | 69 | FEMALE | Head of Pancreas | G1 | T3 | N1 | Stage IIB |
| TCGA-HZ-8519 | 0 | 1.243835616 | 73 | MALE | Head of Pancreas | G3 | T1 | N0 | Stage IA |
| TCGA-HZ-8636 | 1 | 1.493150685 | 58 | FEMALE | Tail of Pancreas | G3 | T3 | N0 | Stage IV |
| TCGA-HZ-A49G | 0 | 1.808219178 | 58 | FEMALE | Head of Pancreas | G2 | T2 | N1 | Stage IIB |
| TCGA-HZ-A49H | 0 | 1.345205479 | 68 | FEMALE | Head of Pancreas | G2 | T3 | N1 | Stage IIB |
| TCGA-HZ-A49I | 1 | 0.843835616 | 77 | MALE | Head of Pancreas | G2 | T3 | N1 | Stage IIB |
| TCGA-HZ-A4BH | 0 | 0.531506849 | 75 | MALE | Head of Pancreas | G3 | T3 | N1b | Stage IIB |
| TCGA-HZ-A4BK | 0 | 1.8 | 72 | MALE | Head of Pancreas | G3 | T3 | N1 | Stage IIB |
| TCGA-HZ-A77O | 1 | 0.438356164 | 77 | FEMALE | Head of Pancreas | G2 | T2 | N1 | Stage IIB |
| TCGA-HZ-A77P | 0 | 0.904109589 | 77 | MALE | Head of Pancreas | G1 | T3 | N1 | Stage IIB |
| TCGA-HZ-A77Q | 0 | 0.090410959 | 55 | FEMALE | Head of Pancreas | G2 | T3 | N1 | Stage IIB |
| TCGA-HZ-A8P1 | 0 | 0.019178082 | 81 | MALE | Tail of Pancreas | G1 | T2 | N0 | Stage IB |
| TCGA-IB-7644 | 1 | 1.079452055 | 65 | FEMALE | Head of Pancreas | G2 | T3 | N1 | Stage IV |
| TCGA-IB-7645 | 1 | 4.115068493 | 44 | FEMALE | Head of Pancreas | G1 | T3 | N1 | Stage IIB |
| TCGA-IB-7646 | 1 | 0.397260274 | 60 | MALE | Head of Pancreas | G2 | T3 | N1 | Stage IIB |
| TCGA-IB-7647 | 1 | 1.824657534 | 41 | MALE | Head of Pancreas | G2 | T3 | N1 | Stage IIB |
| TCGA-IB-7649 | 1 | 1.279452055 | 73 | FEMALE | Head of Pancreas | G2 | T3 | N1 | Stage IIB |
| TCGA-IB-7651 | 1 | 1.652054795 | 64 | FEMALE | Head of Pancreas | G2 | T3 | N1 | Stage IIB |
| TCGA-IB-7654 | 1 | 1.304109589 | 80 | MALE | Head of Pancreas | G2 | T2 | N1 | Stage IIB |
| TCGA-IB-7889 | 1 | 1.317808219 | 85 | FEMALE | Head of Pancreas | G1 | T3 | N1 | Stage IIB |
| TCGA-IB-7890 | 1 | 1.638356164 | 73 | MALE | Tail of Pancreas | G3 | T2 | N0 | Stage IB |
| TCGA-IB-7891 | 0 | 1.775342466 | 49 | FEMALE | Head of Pancreas | G1 | T3 | N1 | Stage IIB |
| TCGA-IB-7897 | 1 | 1.331506849 | 53 | FEMALE | Head of Pancreas | G2 | T3 | N1 | Stage IIB |
| TCGA-IB-8126 | 0 | 0.205479452 | 79 | FEMALE | Head of Pancreas | G1 | T4 | N1 | Stage III |
| TCGA-IB-8127 | 0 | 1.430136986 | 59 | MALE | Head of Pancreas | G2 | T3 | N1 | Stage IIB |
| TCGA-IB-A5SO | 1 | 1 | 71 | MALE | Head of Pancreas | G2 | T3 | N1 | Stage IIB |
| TCGA-IB-A5SP | 0 | 1.320547945 | 77 | MALE | Head of Pancreas | G2 | T3 | N0 | Stage IIA |
| TCGA-IB-A5SQ | 1 | 0.6 | 56 | FEMALE | Head of Pancreas | G2 | T2 | N0 | Stage IB |
| TCGA-IB-A5SS | 1 | 1.260273973 | 64 | FEMALE | Tail of Pancreas | G3 | T3 | N1 | Stage IIB |
| TCGA-IB-A5ST | 0 | 1.739726027 | 64 | FEMALE | Head of Pancreas | G2 | T3 | N1 | Stage IIB |
| TCGA-IB-A6UG | 1 | 0.112328767 | 65 | MALE | Head of Pancreas | G3 | T3 | N1 | Stage IIB |
| TCGA-IB-A7LX | 1 | 0.684931507 | 57 | MALE | Head of Pancreas | G2 | T3 | N1 | Stage IIB |
| TCGA-IB-A7M4 | 0 | 1.323287671 | 81 | MALE | Body of Pancreas | G3 | T3 | N1 | Stage IIB |
| TCGA-IB-AAUM | 0 | 0.021917808 | 76 | MALE | Head of Pancreas | G3 | T2 | N1 | Stage IIB |
| TCGA-IB-AAUN | 1 | 0.394520548 | 74 | FEMALE | Head of Pancreas | G2 | T2 | N0 | Stage IB |
| TCGA-IB-AAUO | 1 | 0.654794521 | 64 | FEMALE | Head of Pancreas | G3 | T3 | N1 | Stage IIB |
| TCGA-IB-AAUP | 0 | 1.180821918 | 68 | MALE | Head of Pancreas | G2 | T3 | N1 | Stage IIB |
| TCGA-IB-AAUQ | 1 | 0.501369863 | 50 | MALE | Tail of Pancreas | G2 | T3 | N1 | Stage IIB |
| TCGA-IB-AAUR | 0 | 0.926027397 | 67 | MALE | Head of Pancreas | G1 | T3 | N1 | Stage IIB |
| TCGA-IB-AAUS | 0 | 0.616438356 | 84 | FEMALE | Pancreas Head and Body | G2 | T3 | N1 | Stage IIB |
| TCGA-IB-AAUT | 0 | 0.78630137 | 65 | MALE | Head of Pancreas | G1 | T2 | N1 | Stage IIB |
| TCGA-IB-AAUU | 0 | 0.671232877 | 35 | MALE | Head of Pancreas | G3 | T3 | N1 | Stage IIB |
| TCGA-IB-AAUV | 0 | 1.106849315 | 49 | MALE | Head of Pancreas | G2 | T3 | N1 | Stage IIB |
| TCGA-IB-AAUW | 1 | 0.630136986 | 63 | FEMALE | Head of Pancreas | G3 | T3 | N1 | Stage IIB |
| TCGA-L1-A7W4 | 1 | 0.761643836 | 48 | FEMALE | Head of Pancreas | G3 | T3 | N1 | Stage IIB |
| TCGA-LB-A7SX | 1 | 1.076712329 | 74 | FEMALE | Head of Pancreas | G2 | T3 | N1 | Stage IIB |
| TCGA-LB-A8F3 | 0 | 1.038356164 | 64 | FEMALE | Head of Pancreas | G1 | T3 | N0 | Stage IIA |
| TCGA-LB-A9Q5 | 1 | 0.857534247 | 63 | FEMALE | Head of Pancreas | G3 | T3 | N1 | Stage IIB |
| TCGA-M8-A5N4 | 0 | 1.6 | 48 | FEMALE | Head of Pancreas | G2 | T3 | N0 | Stage IIA |
| TCGA-OE-A75W | 1 | 0.731506849 | 75 | MALE | Tail of Pancreas | G1 | T3 | N0 | Stage IIA |
| TCGA-PZ-A5RE | 1 | 1.287671233 | 44 | FEMALE | Head of Pancreas | G3 | T3 | N1 | Stage IIB |
| TCGA-Q3-A5QY | 0 | 1.139726027 | 58 | MALE | Head of Pancreas | G2 | T3 | N1 | Stage IIB |
| TCGA-Q3-AA2A | 0 | 0.260273973 | 64 | FEMALE | Head of Pancreas | G1 | T3 | N1 | Stage IB |
| TCGA-RB-A7B8 | 1 | 1.276712329 | 81 | FEMALE | Head of Pancreas | G2 | T3 | N1 | Stage IIB |
| TCGA-RB-AA9M | 0 | 0.783561644 | 43 | MALE | Tail of Pancreas | G3 | T1 | N1 | Stage IIB |
| TCGA-RL-AAAS | 0 | 0.024657534 | 60 | FEMALE | Head of Pancreas | G2 | T2 | N0 | Stage IB |
| TCGA-S4-A8RM | 0 | 1.769863014 | 67 | MALE | overlapping parts of pancreas | G3 | T3 | N1 | Stage IIB |
| TCGA-S4-A8RO | 0 | 1.438356164 | 75 | FEMALE | Head of Pancreas | G2 | T3 | N1 | Stage IIB |
| TCGA-S4-A8RP | 1 | 1.923287671 | 77 | FEMALE | Head of Pancreas | G3 | T3 | N1 | Stage IIB |
| TCGA-US-A774 | 1 | 1.904109589 | 76 | FEMALE | Head of Pancreas | G3 | T3 | N1 | Stage IIB |
| TCGA-US-A776 | 0 | 2.312328767 | 61 | MALE | Head of Pancreas | G2 | T3 | N0 | Stage IIA |
| TCGA-US-A779 | 1 | 1.4 | 54 | FEMALE | Head of Pancreas | G1 | T3 | N1b | Stage IIB |
| TCGA-US-A77E | 1 | 1.178082192 | 73 | MALE | Head of Pancreas | G3 | T3 | N1 | Stage IIB |
| TCGA-US-A77J | 1 | 1.556164384 | 81 | FEMALE | Head of Pancreas | G2 | T3 | N1b | Stage IIB |
| TCGA-XD-AAUG | 0 | 0.44109589 | 66 | FEMALE | distal pancreas | G2 | T3 | N1 | Stage IV |
| TCGA-XD-AAUH | 0 | 0.449315068 | 57 | FEMALE | Head of Pancreas | G2 | T3 | N1 | Stage IIB |
| TCGA-XD-AAUI | 1 | 1.002739726 | 50 | FEMALE | Head of Pancreas | G2 | T3 | N1 | Stage IIB |
| TCGA-XD-AAUL | 0 | 1.109589041 | 56 | MALE | Head of Pancreas | G2 | T3 | N0 | Stage IIA |
| TCGA-YB-A89D | 0 | 0.95890411 | 59 | MALE | Head of Pancreas | G2 | T3 | N1 | Stage IIB |
| TCGA-YH-A8SY | 0 | 1.063013699 | 73 | FEMALE | Head of Pancreas | G2 | T3 | N1 | Stage III |
| TCGA-Z5-AAPL | 0 | 0.057534247 | 74 | FEMALE | Body and Tail | G1 | T3 | N0 | Stage IIA |

| **Regimens** | **Number**  **of patients** | **Administration** | **Interval cycles** |
| --- | --- | --- | --- |
| GEM monotherapy | 13 | GEM was given at a dose of 1,000 m/m^2^; infused intravenously in 30 minutes; once a week for three weeks (d1/d8/d15); | Every 4 weeks |
| GEM combinations | 24 | GEM was given at a dose of 1,000 m/m^2^; infused intravenously in 30 minutes; once a week for two weeks (d1/d8); | Every 3 weeks |
| GEM+FU | 5 | FU was given intravenously at a dose of 425-600 mg/m^2^ for day 1- 5 |  |
| GEM+DDP | 5 | DDP was given intravenously at a dose of 65-80 mg/m^2^ for day1-4 |  |
| GEM+CAP | 7 | CAP was given orally at a dose of 1,000 mg /m^2^; twice a day for day 1-14 |  |
| GEM+OXA | 4 | CAP was given orally at a dose of 1,000 mg /m^2^; twice a day for day 1-14 |  |
| GEM+Nab-paclitaxel | 3 | Nab-paclitaxel was given intravenously at a dose of 125 mg/ m^2^ on day 1 and day 8 |  |

**Supplementary Table S9. Gemcitabine-based first-line chemotherapy for 37 patients (Cohort 2) with advanced pancreatic cancer**

**Supplementary Table S10. Correlation between expression of the miR-135b-BMAL1-YY1 loop and chemoresponse in 37 patients (Cohort 2) with pancreatic cancer**

| **Response** | **Total** | **miR-135b expression** | | ****P*** | **BMAL1 expression** | | ****P*** | **YY1 expression** | | ****P*** |
| --- | --- | --- | --- | --- | --- | --- | --- | --- | --- | --- |
|  |  | **Low** | **High** |  | **Low** | **High** |  | **Low** | **High** |  |
| Evaluation of target lesions | 37 | 16 | 21 |  | 20 | 17 |  | 17 | 20 |  |
| Complete response (CR) | – | – | – |  | – | – |  | – | – |  |
| Partial response (PR) | 6 | 4 | 2 | 0.020* | 1 | 5 | 0.006* | 3 | 3 | 0.054 |
| Stable disease (SD) | 17 | 10 | 7 |  | 7 | 10 |  | 11 | 6 |  |
| Progressive disease (PD) | 14 | 2 | 12 |  | 12 | 2 |  | 3 | 11 |  |
| Objective response rate  (ORR = 16.2%) | 20 | 6 | 14 |  | 13 | 7 |  | 6 | 14 |  |
| CR+PR | 6 | 4 | 2 | 0.019* | 1 | 5 | 0.003* | 3 | 3 | 0.201 |
| PD | 14 | 2 | 12 |  | 12 | 2 |  | 3 | 11 |  |
| Disease control rate  (DCR = 62.2%) | 37 | 16 | 21 |  | 20 | 17 |  | 17 | 20 |  |
| CR+PR+SD | 23 | 14 | 9 | 0.006* | 8 | 15 | 0.003* | 14 | 9 | 0.020* |
| PD | 14 | 2 | 12 |  | 12 | 2 |  | 3 | 11 |  |

* *P* < 0.05 indicates a significant relationship among the variables.

| **Oligonucleotide** | **Sequence from 5’ to 3’** | **Oligonucleotide** | **Sequence from 5’ to 3’** |
| --- | --- | --- | --- |
| hsa-miR-135b-F | CCGCTCTATGGCTTTTCATTC | U6 snRNA-F | ATTGGAACGATACAGAGAAGATT |
| hsa-miR-135b-R | TATGGTTTTGACGACTGTGTGAT | U6 snRNA-R | GGAACGCTTCACGAATTTG |
| Site 1-F | TATGAGGAGGGGTGGCTTCC | p21-F | GGGGAAGGGACACACAAGAAGA |
| Site 1-R | ATCCTGGGTACTGGCAGCAG | p21-R | AATGAACTGGGGAGGGATGG |
| Site 2-F | GACTGGGCCCCCCAGGTTAAATTCT | c-Myc-F | CAGCGACTCTGAGGAGGAACA |
| Site 2-R | CCATTGTGTGAGGCCTTTGT | c-Myc-R | GCTGCGTAGTTGTGCTGATGTG |
| BMAL1-F | TGCCACCAATCCATACACAG | WEE1-F | AGGGAATTTGATGTGCGACAG |
| BMAL1-R | TTCCCTCGGTCACATCCTAC | WEE1-R | TTCAAGCTCATAATCACTGGCT |
| CLOCK-F | AAAATACTCTCTACTCATCTGCTGG | Cyclin D1-F | CTCCTGTGCTGCGAAGTGGA |
| CLOCK-R | ATGGCTCCTTTGGGTCTATTG | Cyclin D1-R | AGACCTCCAGCATCCAGGTG |
| PER1-F | TCTGTAAGGATGTGCATCTGGT | XPA-F | AGCTAGGTCCTCGGAGTGG |
| PER1-R | CAGGCAGTTGATCTGCTGGT | XPA-R | TTAGCCATGCCTCCAGTAGC |
| PER2-F | GCCAGAGTCCAGATACCTTTAG | ERCC1-F | GCAGTCTTTCCCTTGAGGCT |
| PER2-R | TGTGTCCACTTTCGAAGACTG | ERCC1-R | GGAATAAGGGCTTGGCCACT |
| CRY1-F | TTACACTATGCTCATGGCGAC | ATM-F | TGGCCATTGGTGGACATGG |
| CRY1-R | GTGCTCTGTCTCTGGACTTTAG | ATM-R | ATCACTGTCACTGCACTCGG |
| CRY2-F | CGTGTTCCCAAGGCTGTTCA | ATR-F | TTCCGGGAGGAGTTTTGGC |
| CRY2-R | CTCCGTCACTACTTCCACACC | ATR-R | TTTACAAGTTCTACAGCAACAACA |
| DBP-F | TAGAAGGAGCGCCTTGAGTC | Caspase 3-F | GGCATTGAGACAGACAGTGGTG |
| DBP-R | GCAACCCTCCAGTATCCAGA | Caspase 3-R | GGCACAAAGCGACTGGATGA |
| NR1D1-F | GACATGACGACCCTGGACTC | FAS-F | TGAAGGACATGGCTTAGAAGT |
| NR1D1-R | GCTGCCATTGGAGTTGTCAC | FAS-R | GGTGCAAGGGTCACAGTGTT |
| RORA-F | CTAGCTCTTCAACACGTCCTAC | BAX-F | TTTGCTTCAGGGTTTCATCC |
| RORA-R | TCGCACAATGTCTGGGTATATT | BAX-R | CAGTTGAAGTTGCCGTCAGA |
| SIRT1-F | CTCCTCTCTTGACTCGCCAT | BCL2-F | AATCCTGTGCTGCTATCCTGC |
| SIRT1-R | CGGTGTAACCCCTCCAAGTC | BCL2-R | GCGTCCACGTTCTTCATTGTT |
| YY1-F | ATGGCCTCGGGGGACACC | GAPDH-F | GCTCTCTGCTCCTCCTGTTC |
| YY1-R | TCA CTGGTTGTTTTTGGC | GAPDH-R | ACGACCAAATCCGTTGACTC |

**Supplementary Table S11. Primers used in the study**

**Supplementary Figure S1. Evaluation of miRNA levels in human pancreatic cancer (PC) tissues**

**
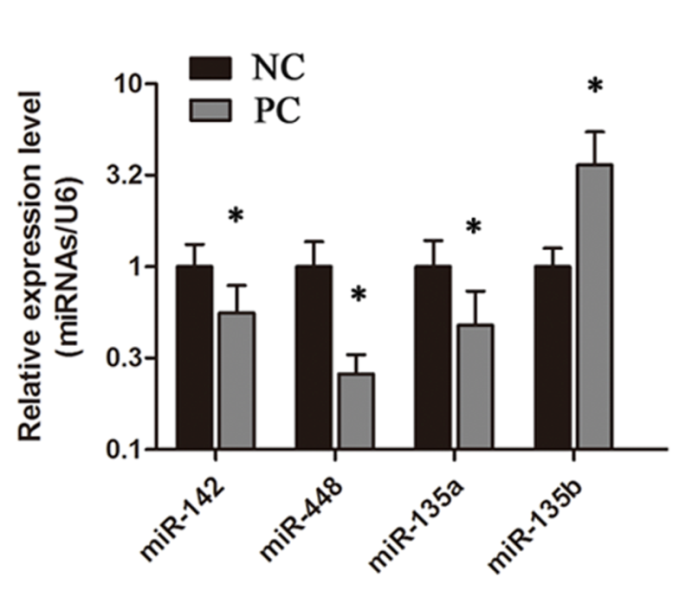
**

Relative expression levels of four selected miRNAs in human PC tissues and normal pancreas (NC) were measured by RT-PCR. U6 snRNA was used as an endogenous control. The results were normalized to the NCs, and the means of the NCs were set to 1. **P* < 0.05.

**Supplementary Figure S2. miR-135b expression in normal and malignant human pancreatic duct epithelial cell lines.**

**
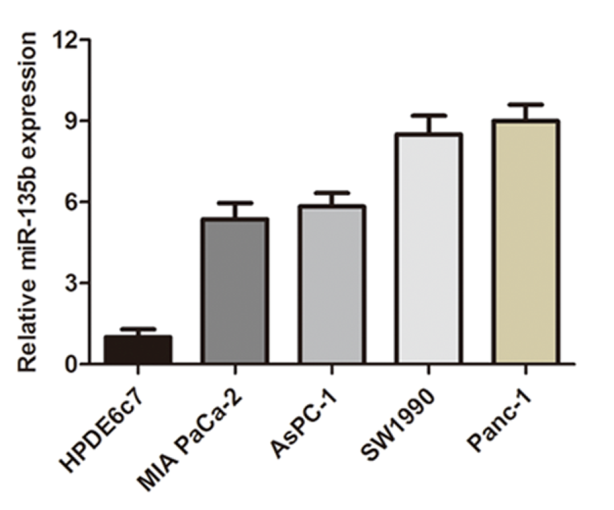
**

The expression level of miR-135b was evaluated by RT-PCR in HPDE6c7 and four human PC cell lines including MIA PaCa-2, AsPC-1, SW1990 and Panc-1. **P* < 0.05. Data are shown as the means ± SEM for three independent experiments.

**Supplementary Figure S3. The role of the miR-135b-BMAL1 axis in circadian gating control of tumor suppression.**

**
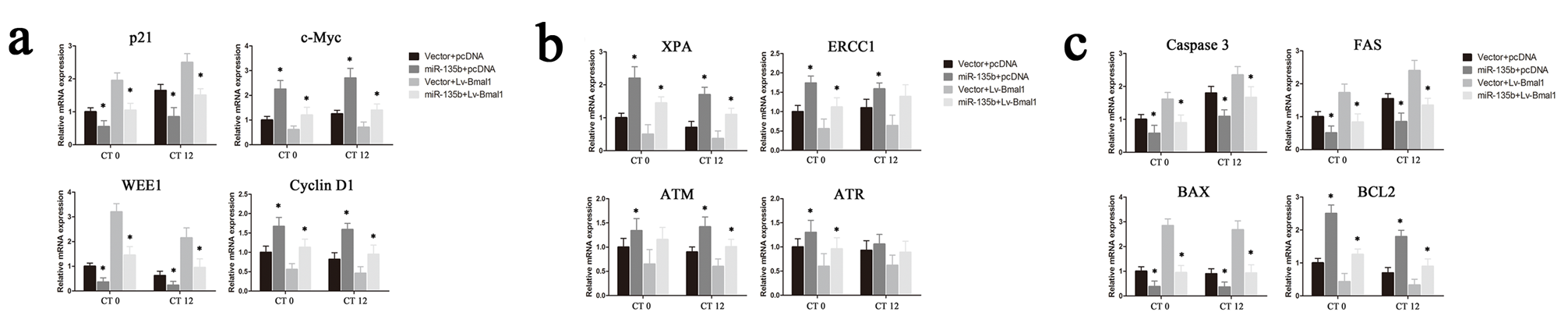
**

MIA PaCa-2 cells were transfected with different combinations of vectors as indicated. The expressions of clock-controlled gene collections of cell cycle (a), DNA damage response (b), and apoptosis (c) were measured by qRT-PCR at circadian time (CT) 0 and CT 12. Data are means ± SEM. **P* < 0.05, miR-135b + pcDNA group *vs*. Vector + pcDNA group; miR-135b + Lv-Bmal1 group *vs*. miR-135b + pcDNA group, at corresponding time points.

**Supplementary Figure S4. BMAL1 expression in stably transfected pancreatic cancer cells**


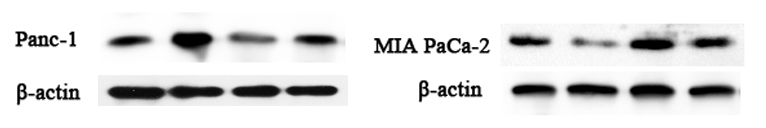


Panc-1 and MIA PaCa-2 cells were stably transfected by different vector combinations and the BMAL1 expressions after transfection were examined by western blot analysis. β-actin was used as an internal control.

The vectors used were as follows:

Panc-1, lane from left to right: Vector+Scramble; Anti-miR-135b+Scramble; Vector+shBmal1; Anti-miR-135b+shBmal1.

MIA PaCa-2, lane from left to right: Vector+pcDNA; miR-135b+pcDNA; Vecotr+Lv-Bmal1; miR-135b+Lv-Bmal1.

**Supplementary Figure S5. Colony formation assay for the proliferation of human pancreatic cancer cells**


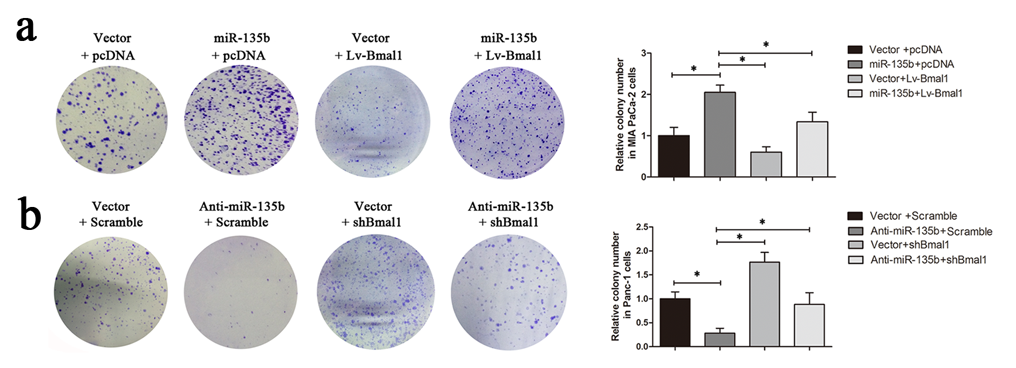


Colony formation assays were used to determine the long-term cell proliferation *in vitro*. The cell colonies in MIA PaCa-2 cells (a) and Panc-1 cells (b) were fixed, stained, and counted after a two-week incubation. Data are representative of at least 3 similar experiments. **P* < 0.05.

**Supplementary Figure S6. Correlation analysis between BMAL1 expression and the miRNA levels in cohort 1 patients**


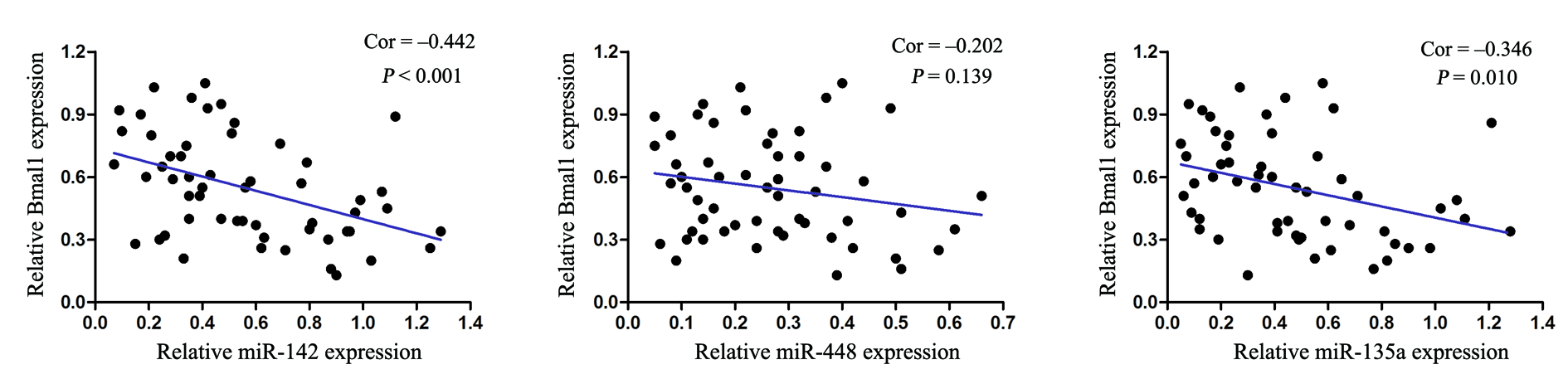


Correlations between the putative BMAL1-targeting miRNAs and BMAL1 mRNA expression were evaluated in pancreatic cancer (PC) tissues from the Cohort 1 patients. The *BMAL1* gene showed a negative correlation with miR-142 (55 cases, Pearson r = –0.442, *P* < 0.001; the left panel), a weak correlation with miR-135a (Pearson r = –0.346, *P* = 0.010; the right panel), and no correlation with miR-448 (Pearson r = –0.202, *P* = 0.139; the middle panel), respectively.
